# Self-complementary double-stranded porphyrin arrays assembled from an alternating pyridyl–porphyrin sequence†
†Electronic supplementary information (ESI) available: Synthesis and full characterization of new compounds, electronic absorption spectra from titration experiments, and full characterization of (**1_1_**)_2_ and (**1_2_**)_2_. See DOI: 10.1039/c5sc01101a


**DOI:** 10.1039/c5sc01101a

**Published:** 2015-08-05

**Authors:** Mitsuhiko Morisue, Yuki Hoshino, Kohei Shimizu, Masaki Shimizu, Yasuhisa Kuroda

**Affiliations:** a Faculty of Molecular Chemistry and Engineering , Kyoto Institute of Technology , Matsugasaki, Sakyo-ku , Kyoto 606-8585 , Japan . Email: morisue@kit.ac.jp ; Fax: +81-75-724-7806

## Abstract

Oligomeric porphyrin arrays with an alternating pyridyl–porphyrin sequence were synthesized to explore double-strand formation through self-complementary pyridyl-to-zinc axial coordination bonds. Competitive titration experiments revealed the thermodynamic aspects involved in the zipper effect within double-strand formation. Multiple axial coordination bonds defined the stacked conformation, despite a marginal contribution to the stability of the double-strands. Thus, the zipper cooperativity was the dominant factor for the remarkable stability. Moreover, the dimeric and trimeric porphyrin arrays were independently assembled into double-strands by self-sorting from a binary mixture. Double-strand formation engineered discretely stacked π-systems. Successive slipped-cofacial stacks of the porphyrin rings progressively extended the π-system *via* exciton coupling over the double-strand while keeping a relatively high fluorescence quantum yield.

## Abstract 2

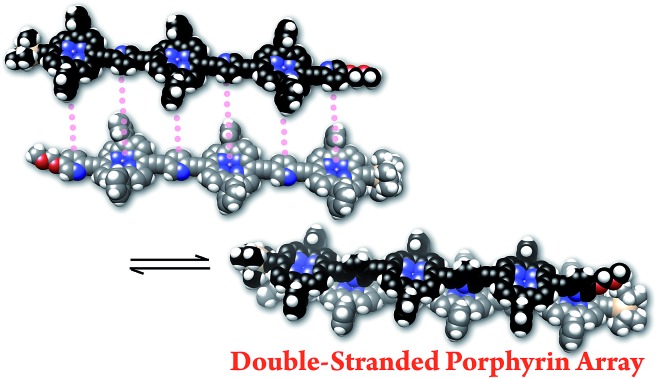
Oligomeric porphyrin arrays with an alternating pyridyl–porphyrin sequence were synthesized to explore double-strand formation through self-complementary pyridyl-to-zinc axial coordination bonds.

## Introduction

Naturally occurring double-stranded polymers, such as DNA and proteins, display an exquisite molecular organization in biological systems. Double-stranded DNA is currently emerging as a passive scaffold and provides state-of-the-art bottom-up nanotechnologies with a molecular scale precision, as exemplified by DNA-origami.^1^ This shape-programmable nanotechnology is enabled by both the sequence-specific double-strand formation and shape-persistent double-stranded building units. Therefore, the development of an intelligent double-strand as a building material for sophisticated hierarchical architectures in which functionalities could be integrated should be of significant interest. The present study examines novel double-strand-forming oligomeric porphyrin arrays which may, in a straightforward manner, be used to incorporate photoelectronic functions into structures for artificial photosynthesis.

The high fidelity of Watson–Crick base pairing *via* complementary hydrogen bonds plays a crucial role in the formation of double-stranded DNA.^2^ In a similar way, artificial double-strands require the molecular design of complementary pairs through either multiple hydrogen bonds^3^ or charge-transfer interactions.^4^ While the majority of research in the field has been devoted to the formation of helical structures,^5^ almost no synthetic effort has been aimed at the applications of these structures in nanomaterial science, though they represent a promising new paradigm in bottom-up molecular assembly. The molecular design of supramolecular building materials could include not only sequence selectivity but also the shape-persistence of the functional double-stranded structures.

For decades, porphyrin frameworks have been part of technological advancements at the forefront of material studies because of their large π-systems and outstanding molar extinction coefficients. Their structural relevance in natural photosynthetic systems has been the source of considerable interest and has driven research in supramolecular multiporphyrin architectures.^6^,^7^ The rigid porphyrin plane offers an ideal platform for versatile multiporphyrin architectures which can be built using both covalent and supramolecular approaches.^8^,^9^ Biomimetic supramolecular porphyrin architectures with slipped-cofacially stacked conformations have been a useful motif for the design of materials with excellent photoelectronic functionalities, as described by Kobuke and coworkers.^10^,^11^ A coordination-directed approach is particularly effective in the assembly of ladder complexes that are composed of fully π-conjugated multiporphyrin arrays *via* the use of bidentate ligands, as demonstrated by Anderson and collaborators.^12^ A double-strand is an intriguing motif to use to create novel artificial photosynthetic systems and engineer discrete stacked π-systems.

**Scheme 1:**
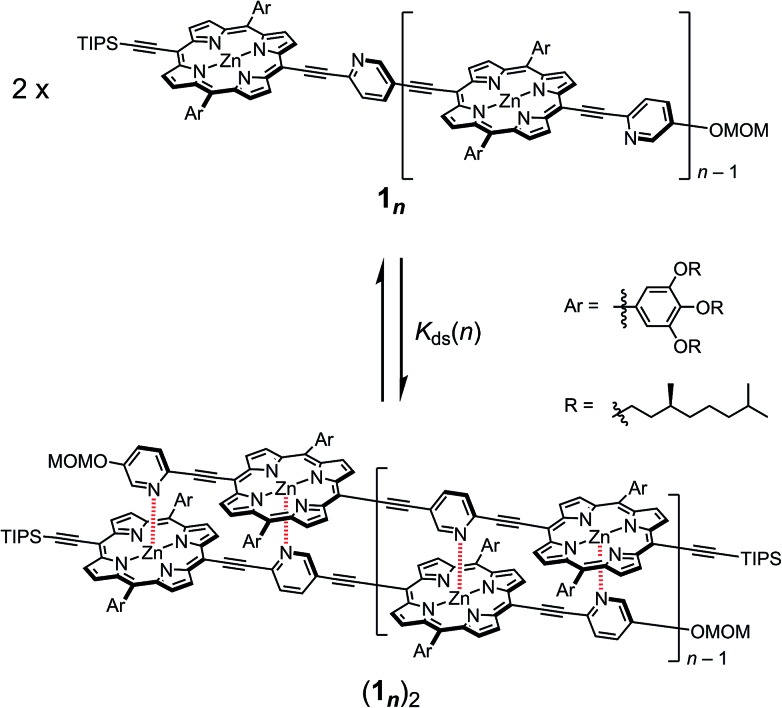
Formation of the double-strands (**1_*n*_**)_2_ (*n* = 1–3).

In our previous report, the formation of self-coordinated zinc(2-pyridylethynyl)porphyrin dimers was controlled by the choice of nucleation conditions, where the formation of the initial coordination bond governed how the second coordination bond would form.^13^ In a non-coordinating solvent, the formation of the second intra-dimer coordination bond was more energetically favorable than the initial binding was, which led to a self-complementary pattern of multiple coordination bonds. According to the same principle, we envisioned that oligomeric zinc(2-pyridylethynyl)porphyrin arrays **1_*n*_** with an alternating pyridyl–porphyrin sequence could be assembled into a double-strand (**1_*n*_**)_2_ (Scheme 1). The formation of the initial interstrand coordination bond would induce the spontaneous formation of the second and the subsequent coordination bonds in a self-complementary fashion, because of the increasingly favorable thermodynamics of binding. This is the so-called zipper effect. Here, we present the molecular organization of novel double-stranded porphyrin arrays based on a self-complementary ligand–metalloporphyrin sequence, which provides successively stacked porphyrin arrays. The photophysical properties of these systems are studied.

## Results and discussion

### Synthesis

**Scheme 2:**
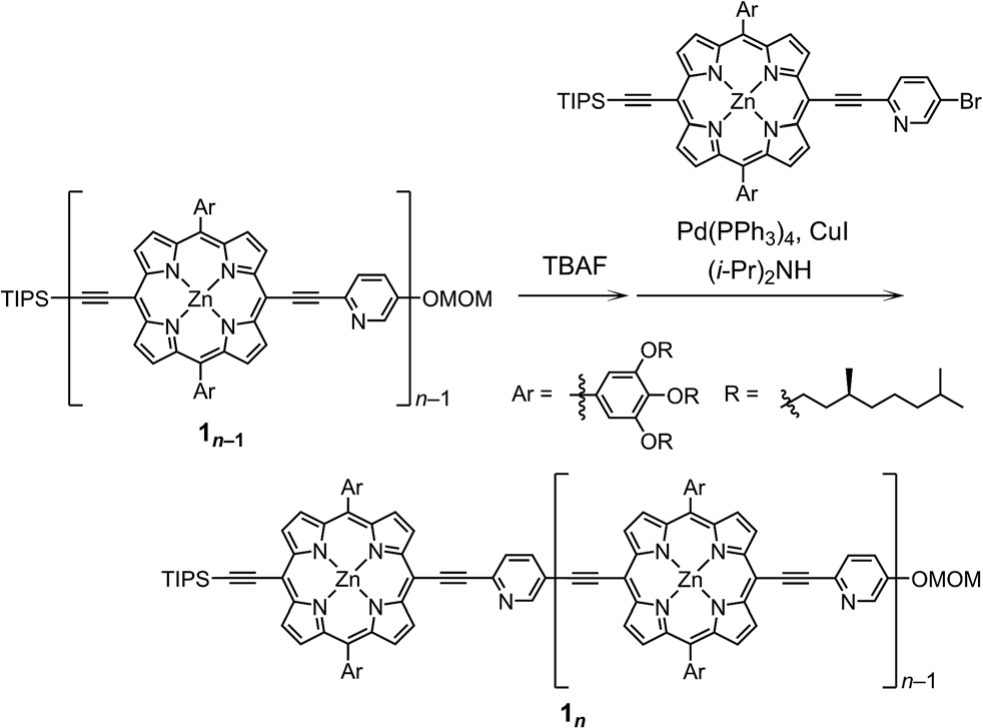
Synthesis of the porphyrin arrays **1_*n*_** (*n* = 2–3).

The synthetic route to the monomeric zinc(2-pyridylethynyl)porphyrin has already been established by our group.^13^ According to the reported procedure, we prepared the monomeric **1_1_** as the precursor for the oligomeric **1_*n*_**. A systematic series of new oligomers **1_*n*_** (*n* = 2–3) were then prepared from **1_1_***via* repetitive Sonogashira–Hagihara coupling reactions (Scheme 2).^14^ The details of the synthetic procedures are described in the ESI,† together with the essential thermodynamic and photophysical properties of (**1_1_**)_2_ and (**1_2_**)_2_. The following sections mainly demonstrate the results representative of (**1_3_**)_2_, which is an assembly composed of six porphyrin rings and six pyridyl groups.

### Structural elucidation of double-strands

**Fig. 1:**
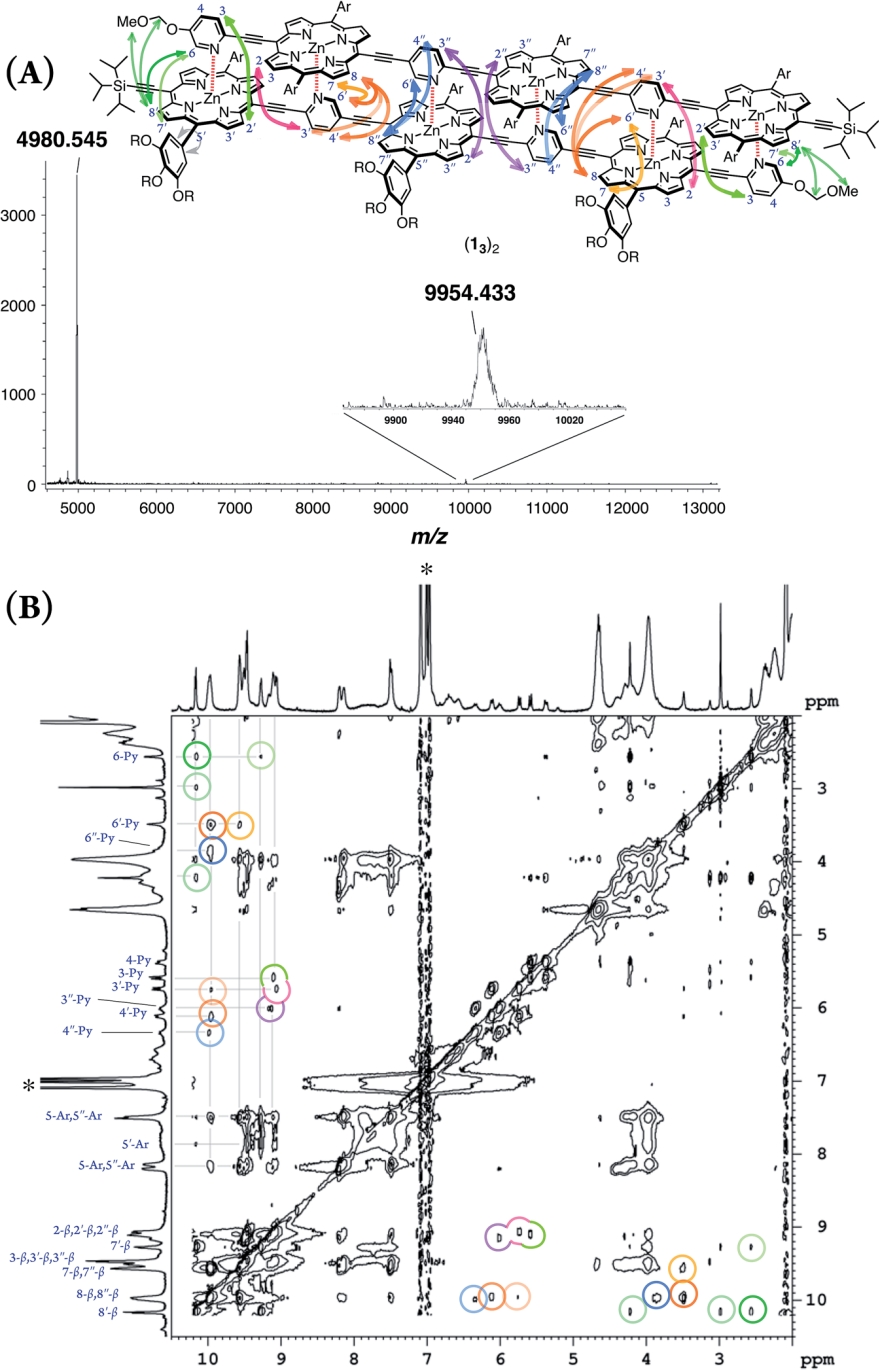
(A) MALDI-TOF MS spectrum of (**1_3_**)_2_. (B) ^1^H–^1^H NOE spectrum of (**1_3_**)_2_ in toluene-*d*_8_. The asterisk indicates residual toluene. One alternative assignment is shown.

double-strand (**1_3_**)_2_ was observed using MALDI-TOF MS measurements (Fig. 1A). (**1_3_**)_2_ spontaneously formed in the lone stationary state in toluene, as identified by NMR. Only three sets of protons in the pyridyl and porphyrin-β positions showed unambiguous diagonal correlations *via* the nuclear Overhauser effect (NOE) (Fig. 1B), which is indicative of two trimeric porphyrin arrays that are cofacially assembled in an antiparallel arrangement (Fig. 1A). NOE correlation with the protons of the MOM group determined one of three porphyrin rings. Subsequently, TOCSY correlations identified two sets of signals corresponding to the 2-, 3- and 6-pyridyl and 2′′-, 3′′- and 6′′-pyridyl protons. An alternative assignment for these two sets was also possible for these pyridyl–porphyrin pairs. However, these pyridyl protons individually showed NOE correlations with the porphyrin rings, which indicated the pairing of the porphyrin rings with the complementary pyridyl groups. This finding is consistent with a symmetrically assembled structure, as indicated by a lack of multiplied signals for the porphyrin array. All of the aromatic resonances of the pyridyl groups were found in the non-aromatic region (6.34–2.56 ppm), which suggested that the axially coordinated pyridyl groups were strongly shielded in the vicinity of the porphyrin ring. The assignment is consistent with all of the observed NMR resonances.

In pyridine-*d*_5_, which can act as a competitive coordinating ligand, the non-shielded resonances of the pyridyl protons of the disassembled species were observed in the aromatic region with the disappearance of the upfield-signals observed for the double-strand. The comparison of the spectra in the two solvents suggests the assembly of double-strand (**1_3_**)_2_ through pyridyl-to-zinc coordination bonds.^14^ All of the NMR data firmly established that the structural picture of the discrete double-strand (**1_3_**)_2_ assembled from an alternating pyridyl–porphyrin sequence *via* self-complementary coordination bonds.

### Thermodynamic behaviors

**Fig. 2:**
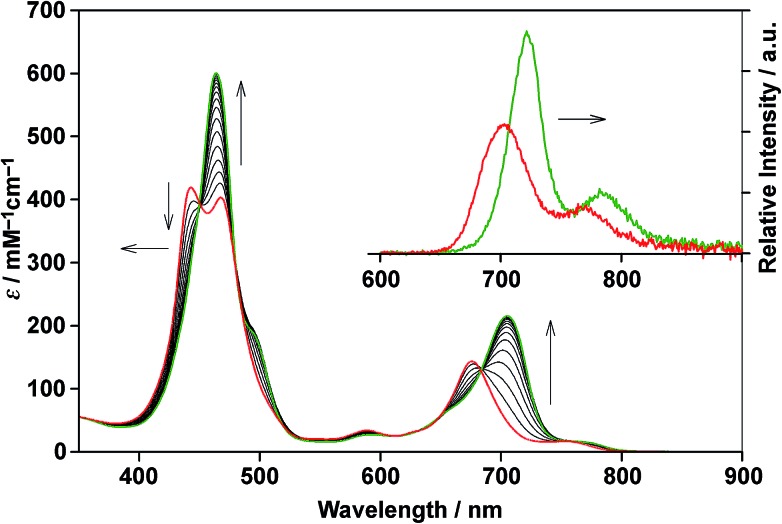
Spectrometric titration of (**1_3_**)_2_ ([**1_3_**]_0_ = 2.9 × 10^–6^ M) with pyridine (up to 480 equiv., red to green) at 25 °C in toluene. The inset shows the fluorescence spectra of (**1_3_**)_2_ and **1_3_** in the presence of excess pyridine (10^4^ equiv.) in toluene (*λ*_ex_ = 450 nm, a pseudo-isosbestic point).

**Scheme 3:**
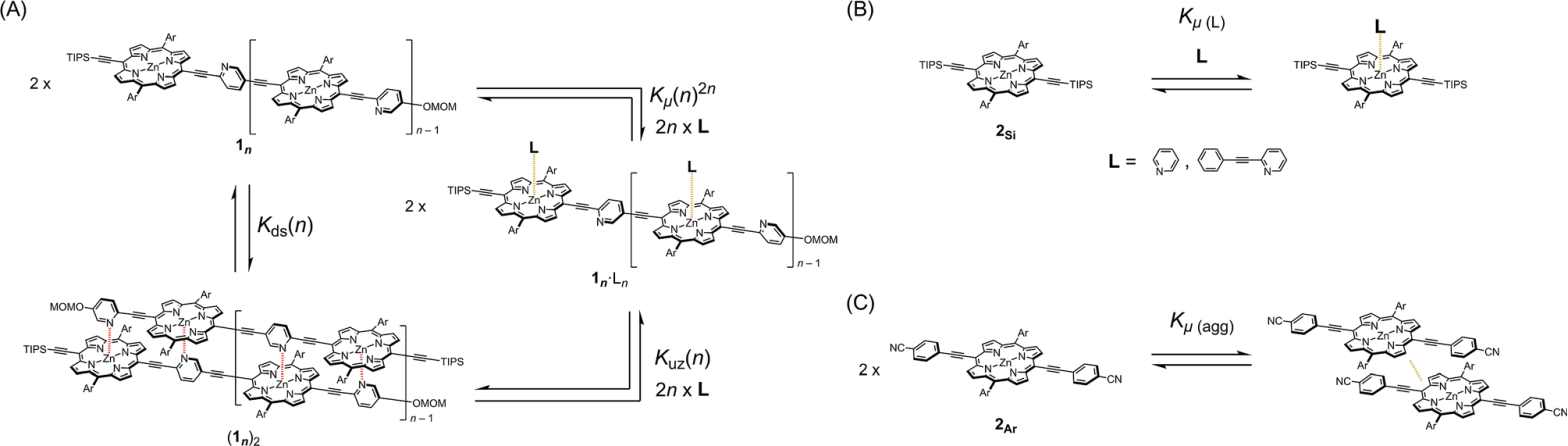
(A) Generic closed thermodynamic cycle (*n* = 2–3). (B) Microscopic binding equilibrium of model porphyrin **2_Si_** with axial ligands; L = pyridine and 2-(phenylethynyl)pyridine. (C) Aggregation equilibrium of model porphyrin **2_Ar_**.

Double-strand (**1_3_**)_2_ was sufficiently durable to obey Beer's law over a wide concentration range (10^–7^ to 10^–4^ M) in toluene. In contrast, the spectral shape of the electronic absorption spectra of **1_1_** depended on the concentration, which suggested a small association constant for the formation of (**1_1_**)_2_ (*K*_ds_(1) = 1.3 ± 0.2 × 10^4^ M^–1^).^14^ The association constant of (**1_3_**)_2_, *K*_ds_(3), was found to be too high to directly evaluate the thermodynamic stability of double-strand (**1_3_**)_2_. Over the course of competitive titration experiments with pyridine, the spectral changes showed several pseudo-isosbestic points, which suggested that the equilibria involved essentially two stationary states, *i.e.*, the double-strand and the unzipped single-strand (Fig. 2). Competitive titration experiments allowed us to analyze the thermodynamic stability of the double-strands according to the thermodynamic cycle (Scheme 3). We analyzed the unzipping equilibria by employing a tentative one-step unzipping model, which is useful for the description of the simplified overall equilibria. Nonlinear least-square fittings gave reliable binding properties for the overall unzipping equilibria.^14^ The overall unzipping constant (*K*_uz_) is then described as follows:1
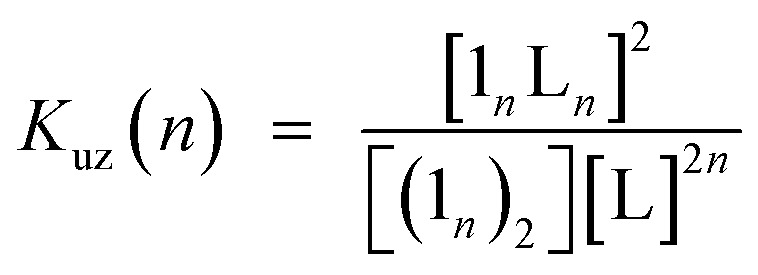



Assuming that the microscopic binding constant (*K*_μ_) is identical for each ligand-to-zinc axial coordination bond of the single-strand, the microscopic binding constant can be approximated by eqn (2).2
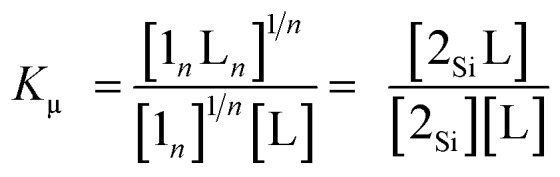



In practice, the titration of model zinc porphyrin **2_Si_** with pyridine to yield the axially coordinated zinc porphyrin gave the experimental *K*_μ_ values (*K*_μ_ = (3.2 ± 0.1) × 10^4^ M^–1^) (Scheme 3B). The values, in turn, gave the binding constant for the double-strand formation (*K*_ds_(*n*)) according to eqn (3).3
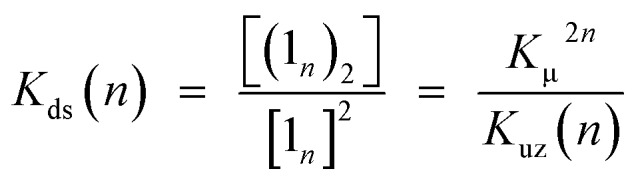



These estimated thermodynamic parameters are useful to the discussion on the durability of double-strand (**1_*n*_**)_2_ (Table 1). The values of *K*_ds_(*n*), *K*_ds_(2) = (2.5 ± 0.3) × 10^9^ M^–1^ and *K*_ds_(3) = (6.5 ± 1.2) × 10^11^ M^–1^, were remarkable, considering the small magnitude of the microscopic binding constant as described below.

**Table 1:** Thermodynamic parameters of **1_*n*_** at 25 °C in toluene

	*n*	*K*_uz_(*n*)^*a*^/M^1–2*n*^	*K*_ds_(*n*)/M^–1^ (Δ*G*°ds(*n*)/kJ mol^–1^)	ΔΔ*G*°(*n*)^*d*^/kJ mol^–1^ (EM^*e*^/M)
**1_1_**	1	—	(1.3 ± 0.2) × 10^4^ (–23 ± 1)^*b*^	–12 ± 0.4 (147 ± 9)
**1_2_**	2	(4.1 ± 0.2) × 10^8^	(2.5 ± 0.3) × 10^9^ (–53 ± 1)^*c*^	–31 ± 0.2 (68 ± 8)
**1_3_**	3	(6.5 ± 1.2) × 10^15^	(1.7 ± 0.3) × 10^11^ (–64 ± 1)^*c*^	–31 ± 0.2 (12 ± 1)

^*a*^Estimated from the competitive titration experiments with pyridine.

^*b*^Directly estimated from variations in concentration.

^*c*^Estimated using eqn (3), wherein *K*_μ_ = (3.2 ± 0.1) × 10^4^ M^–1^.

^*d*^Estimated by employing Δ*G*°μ = –5.5 ± 0.3 kJ mol^–1^, wherein *K*_μ_ = *K*_μ(L)_^1/2^*K*_μ(agg)_^1/2^ = 9.4 ± 1 M^–1^.

^*e*^Effective molarity (EM) was evaluated from eqn (6).

The zipper effect was quantified by the synergetic free energy change (ΔΔ*G*(*n*)), the excess energy beyond the sum of the independent free energy changes induced by pyridyl-to-zinc axial coordinating and π-stacked microscopic binding.^15^

4 ΔΔ*G*(*n*) = Δ*G*_ds_(*n*) – 2*n*Δ*G*_μ_

The ΔΔ*G*(*n*) values substantially dominated the stability of the double-strands, with multiple axial coordination bonds defining the discrete conformation of the double-strand despite their minimal thermodynamic contribution. To quantify a reliable microscopic binding constant, we employed model compounds. The binding constant for the axial coordination of 2-(phenylethynyl)pyridine to **2_Si_** as the model for each ligand-to-zinc axial coordination bond is *K*_μ(L)_ = 7.8 ± 1.1 M^–1^ (Scheme 3B) and that for the self-aggregation of **2_Ar_** as the model for π-stacked interactions is *K*_μ(agg)_ = 11 ± 1 M^–1^(Scheme 3C). The microscopic binding constant for single coordination together with π-stacked interactions was, then, determined to be 9.4 ± 1 M^–1^ (*K*_μ_ = *K*_μ(L)_^1/2^*K*_μ(agg)_^1/2^, Δ*G*°μ = –5.5 ± 0.3 kJ mol^–1^). The dramatic change from ΔΔ*G*°(1) to ΔΔ*G*°(2) suggested that the zipper effect, as a consequence of chelate cooperativity including interactions such as π-stacking, van der Waals interactions, and desolvation entropy, became greater with the addition of the repeating units. In contrast, the ΔΔ*G*°(3) value was similar to that of ΔΔ*G*°(2), which indicated that the compensatory effects gave rise to structural strain, such as the distortion of the porphyrin planes, and a loss of structural entropy. The analyses elucidated that the increasing number of repeat units increased the durability of (**1_*n*_**)_2_, although the cooperativity per interaction decreased. The nucleation step of multiple coordination bonds is dominant when **1_*n*_** self-assembles, and synergetic effects govern the durability of (**1_*n*_**)_2_ for *n* ≤ 3. The self-complementary coordination bonds were significantly stabilized by the zipper effect, thereby thermodynamically funnelling the self-assembled structures into the most stable form without kinetic entrapment by metastable states. This allowed for the realization of the specific double-strand formation.

The situations are alternatively examined in terms of “effective molarity (EM)”, an empirical parameter used to describe the effectiveness of intramolecular interactions, to index the upper limit of concentration for the double-strand formation. The EM value is defined in eqn (5) and (6).^16^,^17^

5 EM = (*K*_ds_(*n*)/*K*_μ_^2*n*^)^1/(2*n*–1)^

6 = exp{–ΔΔ*G*(*n*)/(2*n* – 1)*RT*}

The EM values of (**1_1_**)_2_ and (**1_2_**)_2_ (Table 1) were in the high end range of the typical values for supramolecular systems,^17^ inferring that the model systems were, in the most precise sense, imperfect for the formation of discrete double-strands. However, the EM values predicted a significant selectivity of the double-strand formation even at very concentrated conditions.

### Self-sorting behaviors

**Scheme 4:**
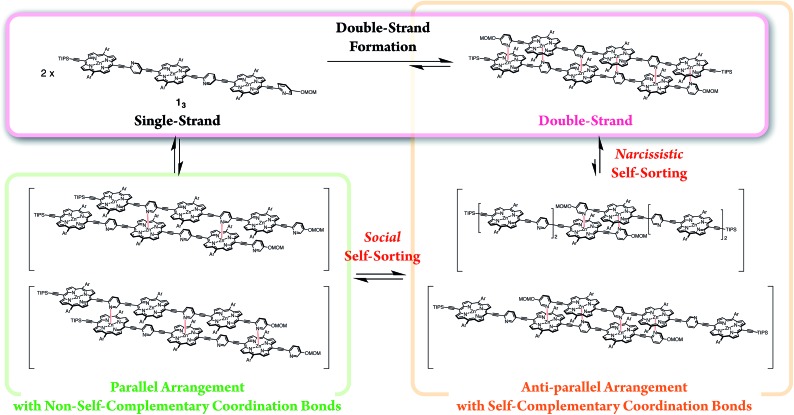
Double-strand formation of **1_3_** self-sorted from possible self-assembled patterns.

The exclusive formation of the self-complementary double-strand (**1_*n*_**)_2_ intrinsically involves self-sorting behaviors due to the possibility of several binding patterns for the self-association of **1_*n*_** (Scheme 4). Briefly, *social* self-sorting favors self-complementary binding patterns, whereas *narcissistic* self-sorting distinguishes differences in the numbers of the repeating units of the self-complementary binding patterns.12b,^18^ Based on the lack of hysteresis, the self-sorting assembly of the double-strands occurred during the heating/cooling processes (25–70 °C) of a binary mixture of (**1_2_**)_2_ and (**1_3_**)_2_.^14^ The electronic absorption spectra appeared as a superimposition of the spectra of (**1_2_**)_2_ and (**1_3_**)_2_ at 25 °C before and after heating, although thermally dissociated **1_2_** and **1_3_** mutually interacted to a degree at 70 °C. The mutually orthogonal self-assembly from a thermally unzipped mixture perfectly restored the initial fluorescence properties, as indicated by the superimposition of (**1_2_**)_2_ and (**1_3_**)_2_ at 25 °C. The self-sorting capacity of double-strands (**1_*n*_**)_2_ makes them promising in the construction of units that are capable of simultaneous multiple molecular assembly within bottom-up nanotechnology applications.

### Engineering discrete stacked π-systems

**Fig. 3:**
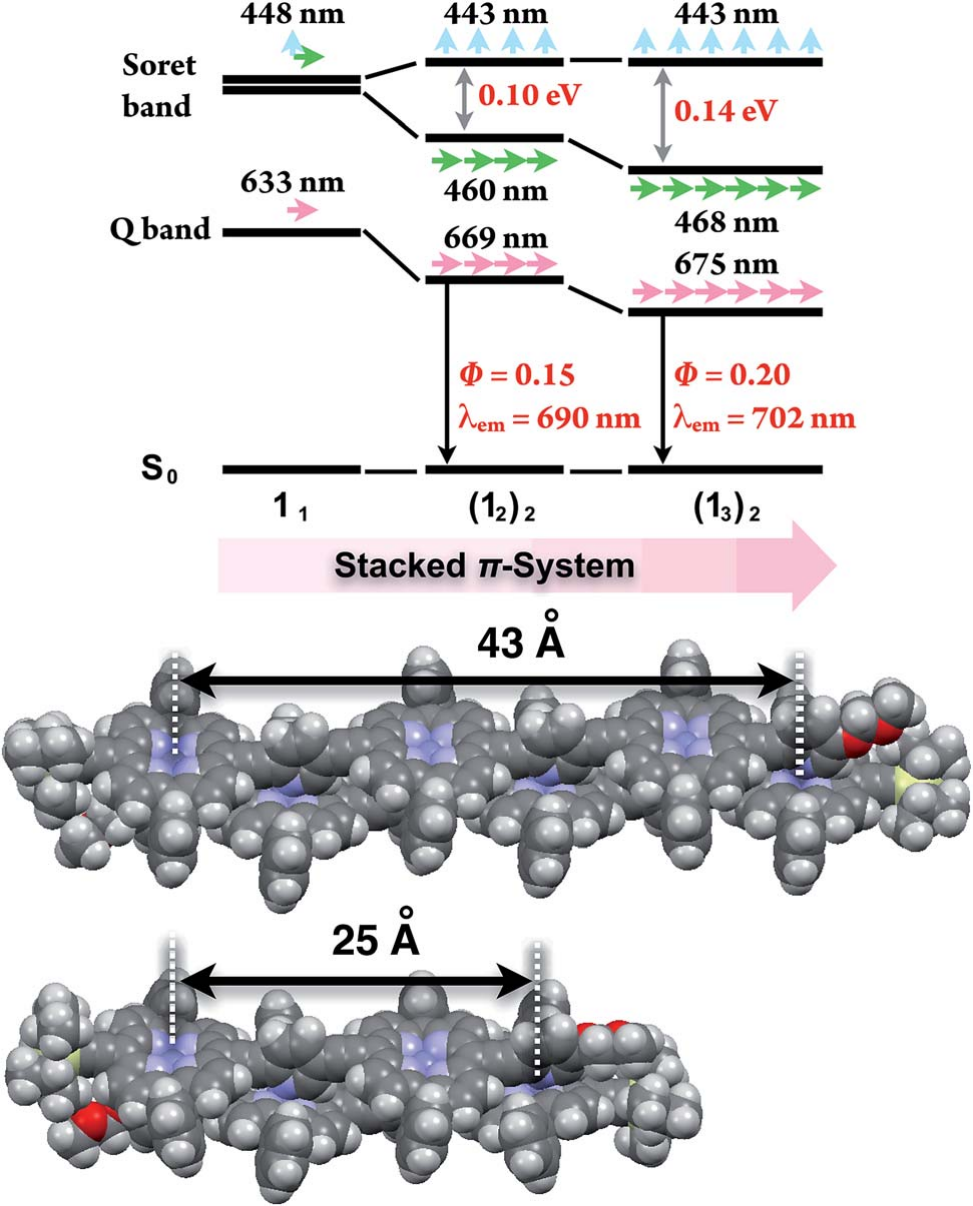
Energy diagrams of the double-strands (**1_*n*_**)_2_, and their geometry-optimized structures calculated using the MM + force field (HyperChem Ver. 8.0 software). The alkoxy side chains are omitted for visual clarity. The wavelengths denote the absorption maxima.

In the last stage, our attention turned to the electronic properties of the double-strands (**1_2_**)_2_ and (**1_3_**)_2_. The double-strand formation of **1_*n*_** resulted in a successively slipped-cofacial stack of porphyrin arrays. The double-strands (**1_*n*_**)_2_ showed a splitting of the Soret band with a bathochromic shift of the lower band due to exciton coupling in the electronic absorption spectra.^10^,^19^ The split width of the Soret band of (**1_3_**)_2_ (0.14 eV) was wider than that of (**1_2_**)_2_ (0.10 eV), where the lowest exciton band was red-shifted and the highest exciton band remained unchanged. At the same time, the Q band of (**1_3_**)_2_ displayed a larger bathochromic shift to 675 nm (1.83 eV) than that of (**1_2_**)_2_ at 669 nm (1.85 eV), and the longest Zn···Zn distances were estimated to be 43 Å for (**1_3_**)_2_ and 25 Å for (**1_2_**)_2_ based on the geometry-optimized structures (Fig. 3). The comparison of the electronic structures of (**1_3_**)_2_ and (**1_2_**)_2_ unveiled that the double-stranded multiporphyrin arrays exhibited exciton coupling due to successive slipped-cofacial stacks, leading to long-range π-electronic interactions.

The emission properties are noteworthy because the double-strands (**1_*n*_**)_2_ did not dissipate a photoexcited singlet. The absolute fluorescence quantum yields (*Φ*) of (**1_2_**)_2_ and of (**1_3_**)_2_ were 0.15 and 0.20, respectively (Table 2). Increasing the number of porphyrin units raised the *Φ* values by extending the π-systems. The relatively high fluorescence efficiency of (**1_*n*_**)_2_ suggested that the double-stranded structure was effective in circumventing nonradiative deactivation pathways, even in the near-infrared wavelength region. The successive slipped-cofacial stacks of the porphyrin planes in the double-strand serve as structural and functional mimics of bacterial light-harvesting antenna complexes which display an efficient capture of sunlight and photoexcited energy transfer.^7^

**Table 2:** Photophysical properties of double-strands (**1_*n*_**)_2_, **1_*n*_** accommodated with pyridine (**1_*n*_**·Py_*n*_) and **2** in toluene

	*λ*_em_ ^*a*^/nm (*Φ*)	*τ*/ns (*α*)	*k*_em_ ^*e*^/s^–1^
(**1_2_**)_2_	690 (0.15)	0.65 (0.51), 1.15 (0.49)^*b*^	1.7 × 10^8^
(**1_3_**)_2_	702 (0.20)	0.56 (0.32), 1.03 (0.68)^*b*^	2.3 × 10^8^
**2_Si_**	635 (0.07)	1.61^*c*^	4.3 × 10^7^
**2_Si_**·Py	650 (0.08)	1.45^*d*^	5.6 × 10^7^

^*a*^Emission maximum (*λ*_em_) and fluorescence quantum yield (*Φ*) obtained using an integration sphere (excitation at 452 nm for (**1_2_**)_2_, 450 nm for (**1_3_**)_2_, and 405 nm for **2_Si_** and **2_Si_**·Py).

^*b*^Fluorescence lifetime (*τ*) and the normalized amplitude (*α*) determined from the fluorescence decay profiles in the range of 623–773 nm upon excitation at 483 nm (Fig. 4).

^*c*^Emission at 635 nm upon excitation at 405 nm.

^*d*^Emission at 650 nm upon excitation at 405 nm.

^*e*^Radiative rate constant defined as *k*_em_ = *Φ*/*τ*. The single-strand **2_Si_**·Py was observed in the presence of excess pyridine (10^4^ equiv.).

**Fig. 4:**
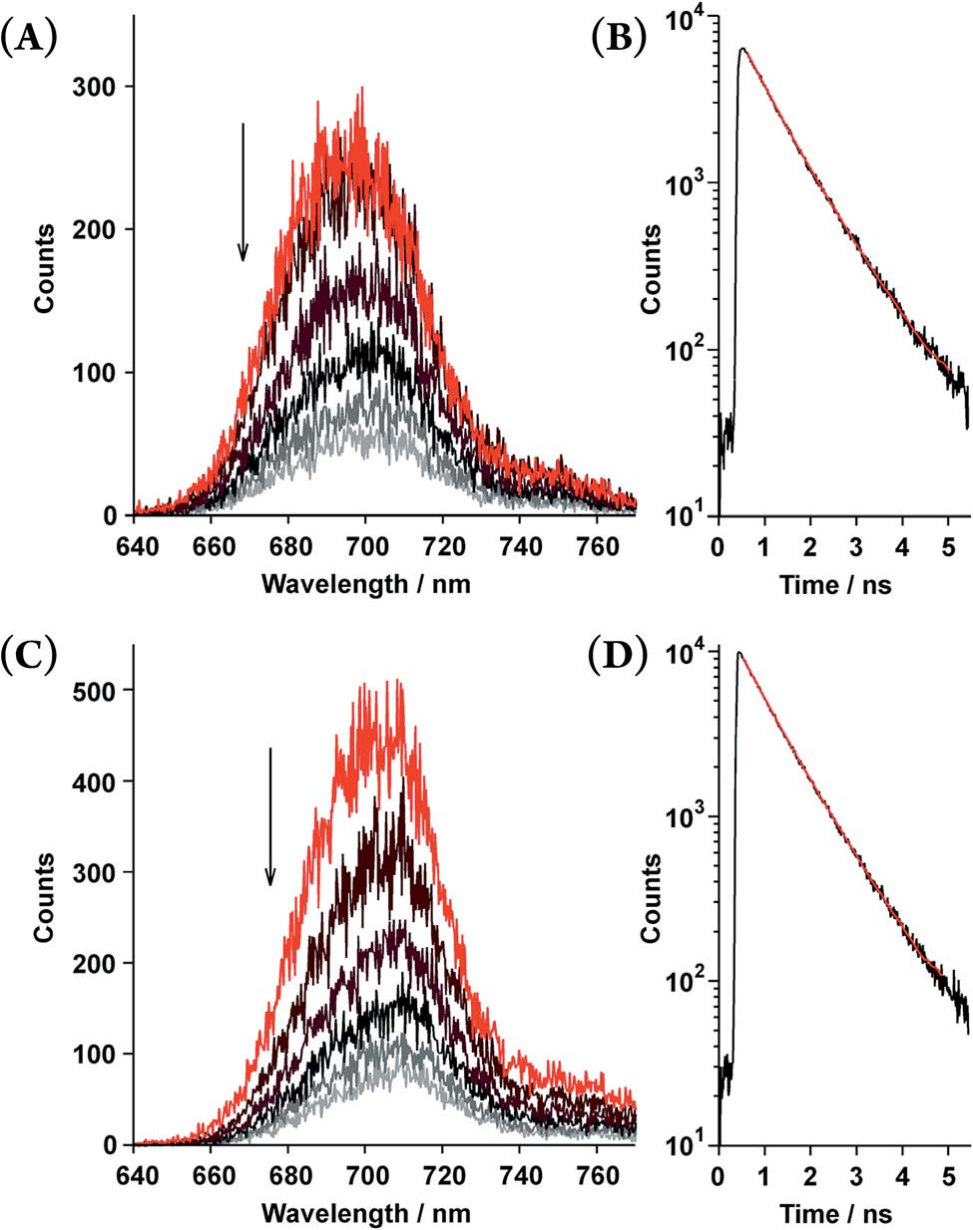
Time-resolved fluorescence spectra recorded every 0.3 ns (upper to lower; 0.45–0.55, 0.75–0.85, 1.05–1.15, 1.35–1.45, 1.65–1.75, and 1.95–2.05 ns) and fluorescence decay profiles of the double-strands (**1_2_**)_2_ at [**1_2_**] = 5.5 × 10^–6^ M (A and B) and (**1_3_**)_2_ at [**1_3_**] = 6.9 × 10^–6^ M (C and D) in toluene. The fluorescence decay profiles (black lines) are shown with fitted curves based on the biexponential decay constants (red lines, *τ* shown in Table 2) in the range of 623–773 nm.

Time-resolved fluorescence spectroscopy gave further insight into the photophysical dynamics of the excited states (Fig. 4). The biexponential fluorescence decay profiles of both double-strands indicated the existence of dual fluorescent states (Table 2). Over time, double-strand (**1_3_**)_2_ showed a small red-shift in its emission wavelength. In contrast, the spectral shift of (**1_2_**)_2_ was smaller than that of (**1_3_**)_2_. It is intriguing to consider that the photophysical properties are relevant to the thermodynamic aspect of the double-strands; (**1_3_**)_2_ was much more strained than (**1_2_**)_2_. Structural relaxation could provide an energy sink that would be capable of trapping photoexcited singlets. For instance, it is known that porphyrin planes are ruffled in the photoexcited singlet state.^20^,^21^ This interpretation is a likely reason as to why the slow fluorescence decay manifests as a red shift.

## Conclusions

In summary, we have synthesized double-stranded porphyrin arrays that yielded successive slipped-cofacial stacks with strong exciton coupling. Titration experiments revealed the thermodynamic aspects underlying the specific double-strand formation, and are used to describe a process in which self-complementary coordination bonds define the discrete structure. The zipper effect dominated the stability of the double-stranded structure. The remarkable selectivity for double-strand formation may serve as a powerful building tool for sophisticated bottom-up molecular assemblies with molecular scale precision, similar to those found in DNA nanotechnology. Moreover, the double-strands extended the π-electron network without any dissipation of the fluorescence properties due to the assembly of successive slipped-cofacial stacks. The shape-persistent double-stranded porphyrin arrays provide new options for artificial photosynthetic systems based on shape-programmable, and bottom-up molecular architectures.

## Supplementary Material

Supplementary information
