# Supplementary material for: Self-complementary double-stranded porphyrin arrays assembled from an alternating pyridyl–porphyrin sequence
Source: Chem Sci. 2015 Aug 5;6(11):6199–206. doi: 10.1039/c5sc01101a (PMC6054142; doi:10.1039/c5sc01101a)
Supplement: Supplementary file 1 [file SC-006-C5SC01101A-s001.pdf]

Electronic Supplementary Material (ESI) for Chemical Science.
This journal is © The Royal Society of Chemistry 2015

Supporting Information for

Self-Complementary Double-Stranded Porphyrin Arrays Assembled from an Alternating Pyridyl–Porphyrin Sequence

Mitsuhiko Morisue,* Yuki Hoshino, Kohei Shimizu, Masaki Shimizu, and Yasuhisa Kuroda

Faculty of Molecular Chemistry and Engineering, Kyoto Institute of Technology, Matsugasaki, Sakyo-ku, Kyoto 606-8585, Japan.

Contents

1. Synthetic Procedures ········· S1
2. Equilibrium Analyses ········· S8
3. Self-Sorting Properties ········· S10
4. 2D NMR Spectra ········· S11
5. NMR Charts of Newly Synthesized Compounds ········· S13

1. General Methods: THF, dichloromethane, and toluene were dried by passing through several desiccant system.[S1] Triethylamine (Et_3N), diethylamine (Et_2NH) and diisopropylamine ((*i*-Pr)$_2$NH) were distilled over calcium hydride under inert atmosphere prior to use. NMR spectra were recorded on Brüker AV-300. Matrix-assisted laser-dissociation ionization time-of-flight (MALDI–TOF) mass spectra were recorded on mass spectrometer (Brüker, Autoflex Speed). UV/vis absorption spectra were recorded on a spectrophotometer (Shimadzu, UV-3100PC and UV-1800) equipped with a Peltier thermoelectric temperature controlling unit (Shimadzu, TCC-240A). Fluorescence spectra and absolute quantum yields were performed by an absolute PL quantum yield measurement system (Hamamatsu Photonics, C9920-02). Optical rotation was recorded at 589 nm (the sodium D-line) at 20 °C on a polarimeter (JASCO, P-2100) by using a cuvette with 10-cm path length. Time-resolved fluorescence measurements were carried out by employing a circularly polarized beam of 200-fs laser pulse at 483 nm, second harmonic generation (Spectra Physics, Model 3980) of a continuous wave (CW) from 200-fs Ti:sapphire laser (Spectra Physics, Mai Tai), and a streak camera (Hamamatsu Photonics, Streak Scope C4334) to detect the fluorescence, wherein the time-resolution was approximately 30 ps.

2. Synthetic Procedures

2.1. Synthesis of methyl 3,4,5-tri[(3*S*)-(3,7-dimethyloctoxy)]benzoate, 3:

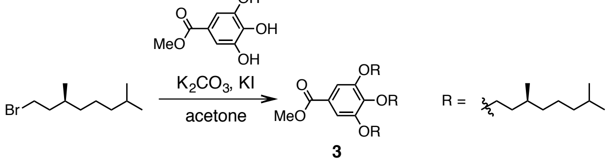

A mixture of methyl gallate (2.6 g, 14 mmol), (3*S*)-3,7-dimethyloctylbromide (derived from *S*-(−)-*β*-citronellol according to the literature method;[S2] 11 g, 48 mmol), potassium carbonate (6.7 g, 48 mmol) and potassium iodide (0.80 g, 4.8 mmol) was refluxed in acetone (110 mL) for 5 days. The reaction mixture dissolved in chloroform was successively washed with saturated aqueous sodium bicarbonate and brine. The crude product extracted with ethyl acetate was purified by silica gel column chromatography with *n*-hexane/ethyl acetate (10/1, v/v) as the eluent. The compound **3** was obtained as colorless turbid liquid (8.2 g, 14 mmol) in 98% yield. ^{1}H NMR (300 MHz, in $CDCl_3$): δ 7.26 (s, 2H; Ar, undistinguishable with the perfectly overlapped signal of residual $CHCl_3$), 4.07–4.00 (m, 6H; ArOCH_2-), 3.89 (s, 3H; Me), 1.92–1.15 (m, 30H; aliphatic chains), 0.94 (d, J = 6.6 Hz, 9H; 3-Me), 0.85 (d, J = 1.5 Hz, 18H; 7-Me); ^{13}C NMR (75 MHz, in $CDCl_3$): δ 166.5, 160.7, 152.7, 142.3, 124.6, 107.8, 76. 8, 71.4, 67.2, 62.1, 51.7, 39.3, 39.2, 39.1, 37.4, 37.31, 37.29, 37.3, 37.0, 36.3, 35.4, 29.7, 29.6, 29.5, 24.7, 24.5, 22.6, 22.54, 22.51, 19.5; specific rotation: $[\alpha]_D^{20}$ = −0.82 (c 1.00 × 10^{-2} g·mL^{-1} in EtOH).

1.3. Synthesis of 3,4,5-tri[(3*S*)-(3,7-dimethyloctoxy)]phenylmethanol, 4:

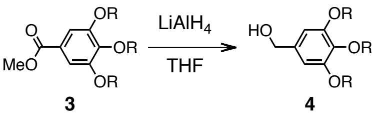

To a suspension of lithium aluminium hydride (1.5 g, 40 mmol) in THF (37 mL) was added dropwise a solution of the benzoate **3** (8.2 g, 14 mmol) in THF (30 mL) at 0 °C under argon atmosphere, followed by stirring at room temperature for 38 h. The reaction was carefully quenched by addition of the knead mixture of sodium sulfate/Celite/water (7/7/9, w/w/w) until hydrogen gas was no longer generated. The reaction mixture was passed through a Celite pad to remove the precipitate. The compound **4** was obtained as colorless turbid liquid

(6.3 g, 11 mmol) in 89% yield. ^{1}H NMR (300 MHz, in $CDCl_3$): δ 6.57 (s, 2H; Ar), 4.60 (d, J = 6.0 Hz, 2H; ArCH_2OH), 4.04–3.92 (m, 6H; ArOCH_2-), 1.89–1.09 (m, 30H; aliphatic chain), 0.93 (d, J = 6.6 Hz, 9H; 3-Me), 0.87 (d, J = 6.6 Hz, 18H; 7-Me); ^{13}C NMR (75 MHz, in $CDCl_3$): δ 153.1, 137.1, 136.5, 105.0, 71.6, 67.2, 65.1, 39.9, 39.4, 39.3, 37.6, 37.40, 37.37, 37.34, 36.5, 29.8, 29.7, 29.5, 28.0, 24.7, 22.7, 22.6, 19.6, 19.6; specific rotation: $[\alpha]_D^{20}$ = –3.06 (c 9.78 × 10^{-3} g·mL^{-1} in EtOH).

1.4. Synthesis of 3,4,5-tri[(3*S*)-(3,7-dimethyloctoxy)]benzaldehyde, 5:

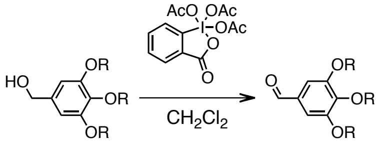

4 5

To a solution of the benzylalcohol **4** (2.5 g, 4.3 mmol) in dichloromethane (25 mL) was added dropwise a solution of the Dess–Martin periodinane (1,1,1-triacetoxy-1,1-dihydro-1,2-benziodoxol-3(1H)-one;[S3] 2.0 g, 4.7 mmol) in dichloromethane (20 mL) at room temperature under nitrogen atmosphere. The mixture was stirred at room temperature for 2 h, and then neutralized with saturated aqueous sodium bicarbonate. The organic layer separated was washed with brine, and dried over anhydrous magnesium sulfate. The titled product **5** was obtained as colorless turbid liquid (2.2 g, 3.9 mmol) in 89% yield. ^{1}H NMR (300 MHz, in $CDCl_3$): δ 9.84 (s, 1H; -CHO), 7.10 (s, 2H; Ar), 4.15–4.02 (m, 6H; ArOCH_2-), 1.94–1.14 (m, 30H; aliphatic chain), 0.97 (d, J = 3.6 Hz, 9H; 3-Me), 0.85 (d, J = 1.5 Hz, 18H; 7-Me); ^{13}C NMR (75 MHz, in $CDCl_3$): δ 201.9, 190.6, 153.4, 143.6, 131.5, 107.5, 76.8, 71.5, 67.2, 39.3, 39.2, 38.9, 37.4, 37.32, 37.27, 37.24, 37.0, 36.2, 29.7, 29.5, 27.9, 24.7, 22.6, 22.5, 19.9, 19.5; specific rotation: $[\alpha]_D^{20}$ = –3.38 (c 9.72 × 10^{-3} g·mL^{-1} in EtOH).

1.5. Synthesis of 5,15-bis[3,4,5-tri{(3*S*)-(3,7-dimethyloctoxy)}phenyl]porphyrinatozinc(II), 6:

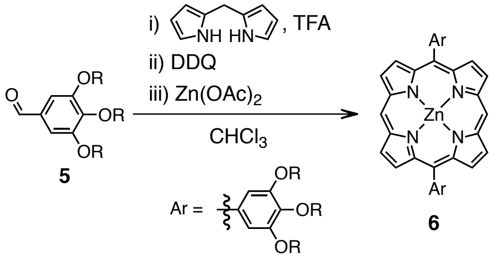

A mixture of dipyrromethane (prepared by the literature method;[S4] 0.66 g, 4.4 mmol) and the benzaldehyde **5** (2.1 g, 4.0 mmol) in chloroform (600 mL) was deaerated by nitrogen bubbling for 5 min. The mixture was added dropwise TFA (trifluoroacetic acid; 0.30 mL, 1.8 mmol) as an acid catalyst and stirred at 25 °C under the dark for 17 h, before addition of DDQ (2,3-dichloro-5,6-dicyanobenzoquinone; 1.7 g, 7.9 mmol) as the oxidant and additional stirring for 1 h. The reaction mixture was neutralized with triethylamine (0.60 mL, 4.0 mmol), and concentrated to approximately one-sixth of the initial volume under the reduced pressure. The crude solution was mixed with slica gel (ca. 100 g), and the resultant slurry was loaded to short silica plug with chloroform. The eluted residue containing the target free-base porphyrin in chloroform was added zinc acetate saturated in methanol (6 mL), and then concentrated with gentle heating under the reduced pressure. The crude product was purified by silica gel column chromatography with toluene, followed by size–exclusion chromatography (Bio-Rad Laboratories, BioBeads® S-X1) with toluene as the eluent. The porphyrin **6** was obtained as viscous reddish purple substance (2.0 g, 1.3 mmol) in 72% yield. ^{1}H NMR (300 MHz, in $CDCl_3$): δ 10.34 (s, 2H; *meso*-porphyrin), 9.45 (d, J = 4.5 Hz, 4H; porphyrin-β), 9.26 (d, J = 4.5 Hz, 4H; porphyrin-β), 7.50 (s, 4H; Ar), 4.42–4.30 (m, 4H; p-ArOCH_2-), 4.19–4.14 (m, 8H; m-ArOCH_2-), 2.12–1.10 (m 60H; aliphatic chains), 0.94 (d, J = 6.3 Hz, 18H; 3-Me), 0.92 (d, J = 6.0 Hz, 12H; 7-Me), 0.82 (d, J = 6.6 Hz, 24H; 7-Me); ^{13}C NMR (75 MHz, in $CDCl_3$): δ 151.2, 150.3, 149.6, 137.9, 137.7, 132.7, 131.8, 120.3, 114.5, 106.3, 72.1, 67.7, 39.6, 39.3, 37.8, 37.7, 37.5, 36.6, 30.0, 30.0, 28.2, 28.1, 25.0, 24.8, 22.9, 22.8, 22.7, 19.9, 19.8, 19.7. MALDI–TOF MS (dithranol): m/z calcd for $C_{92}H_{140}N_4O_6Zn$: 1461.01; found 1461.46 $[M]^+$. λ_{max}/nm (ε/μM^{-1}cm^{-1}, in $CHCl_3$) 415 (0.32), 540 (0.02), 578 (0.04).

1.6. Synthesis of 5,15-dibromo-10,20-bis[3,4,5-tri{(3*S*)-(3,7-dimethyloctoxy)}phenyl]porphyrinatozinc(II), 7:

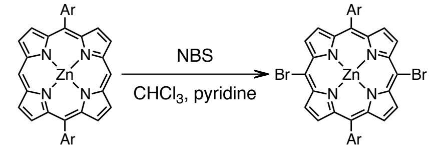

6 7

To a solution of the porphyrin **6** (0.62 g, 0.42 mmol) in chloroform/pyridine (100/1, v/v, 180 mL) was added dropwise a solution of NBS (N-bromosuccinimide; 0.15 g, 0.84 mmol) in chloroform/pyridine (100/1, v/v, 20 mL) with stirring at room temperature. The mixture was stirred at room temperature for 20 min, before the reaction was quenched by addition of acetone. The titled compound was subjected to column chromatography over silica gel with n-hexane/ethyl acetate (10/1, v/v) as the eluent. The porphyrin **7** was obtained as bluish purple substance (0.63 mg, 0.39 mmol) in 92% yield. ^{1}H NMR (300 MHz, in $CDCl_3$): δ 9.72 (d, J = 4.8 Hz, 4H; porphyrin-β), 9.05 (d, J = 4.8 Hz, 4H; porphyrin-β), 7.36 (s, 4H; Ar), 4.31–4.25 (m, 4H; p-ArOCH_2-), 4.17–4.04 (m, 8H; m-ArOCH_2-), 2.07–1.10 (m, 60H; aliphatic chains), 0.94 (d, J = 6.9 Hz, 18H; 3-Me), 0.92 (d, J = 7.2 Hz, 12H; 7-Me), 0.82 (d, J = 6.6 Hz, 24H; 7-Me); ^{13}C NMR (75 MHz, in $CDCl_3$): δ 151.0, 150.7, 150.3, 137.3, 137.2, 133.7, 133.1, 122.3, 114.4, 105.3, 71.8, 67.5, 39.6, 39.3, 37.7, 37.4, 36.3, 28.2, 28.0, 29.9, 24.8, 22.9, 22.8,

22.7, 19.8, 19.6, 19.6. MALDI–TOF MS (dithranol): *m/z* calcd for $C_{92}H_{138}N_4O_6Br_2Zn$: 1616.83; found 1617.86 $[M+H]^+$. λ_{max}/nm ($\varepsilon/\mu M^{-1}cm^{-1}$, in $CHCl_3$) 430 (0.70), 565 (0.03), 606 (0.02).

1.7. Synthesis of 2-bromo-5-(methoxymethoxy)pyridine, 8:

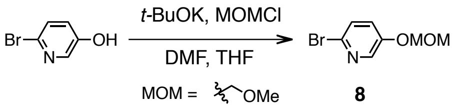

To a solution of 2-bromo-5-hydroxypyridine (1.1 g, 6.2 mmol) in DMF (30 mL) and THF (15 mL) was added potassium *tert*-butoxide (0.84 g, 7.5 mmol) at –15 °C with stirring for 10 min. Then, chloromethyl methylether (0.57 mL, 7.5 mmol) was dropwisely added to the mixture at –15 °C, followed by additional stirring at room temperature for 40 min. The mixture was diluted with chloroform and washed with water. The organic layer separated was dried over anhydrous magnesium sulfate. The target compound was eluted from silica gel column chromatography with dichloromethane as the eluent. The compound **8** was obtained as pale yellow viscous liquid (1.2 g, 6.1 mmol) in 98% yield. ^{1}H NMR (300 MHz, in $CDCl_3$): δ 8.18 (d, *J* = 3.0 Hz, 1H; pyridyl), 7.38 (d, *J* = 8.7 Hz, 1H; pyridyl), 7.27 (dd, *J* = 7.28, 3.0 Hz, 1H; pyridyl), 5.18 (s, 2H; -OCH_2OMe), 3.48 (s, 3H; -OCH$_2$O*Me*); ^{13}C NMR (75 MHz, in $CDCl_3$): δ 153.02, 138.99, 132.80, 127.90, 126.13, 94.47, 55.95.

1.8. Synthesis of 3-(methoxymethoxy)-6-(trimethylsilylethynyl)pyridine, 9:

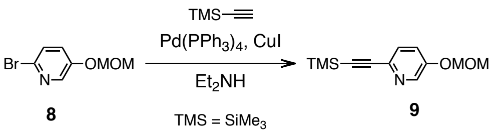

In a Schlenk flask, a mixture of **8** (0.20 g, 1.0 mmol), CuI (9.8 mg, 52 μmol, 5 mol%) in Et_2NH (6 mL) was degassed by successive freeze–pump–thaw cycles, before purging with argon and adding $Pd(PPh_3)_4$ (60 mg, 52 μmol, 5 mol%). The mixture was stirred at 50 °C for 78 h. The mixture was diluted with dichloromethane, and washed with water. The organic layer separated was dried over anhydrous magnesium sulfate. The target compound was eluted from silica gel column chromatography with dichloromethane as the eluent. The compound **9** was obtained as pale yellow viscous liquid (0.15 g, 0.60 mmol) in 59% yield. ^{1}H NMR (300 MHz, in $CDCl_3$): δ 8.34 (dd, *J* = 2.8, 0.5, 1H; pyridyl), 7.40 (dd, *J* = 8.6, 0.5 Hz, 1H; pyridyl), 7.31 (dd, *J* = 8.6, 2.8 Hz, 1H; pyridyl), 5.21 (s, 2H; -OCH_2OMe), 3.48 (s, 3H; -OCH$_2$O*Me*), 0.26 (s, 9H; TMS); ^{13}C NMR (75 MHz, in $CDCl_3$): δ 153.14, 139.99, 136.37, 128.01, 122.74, 103.76, 94.67, 93.32, 56.45, 0.00.

1.9. Synthesis of 5-bromo-15-{5-(methoxymethoxy)pyrid-2-yl}ethynyl-10,20-bis[3,4,5-tri{(3*S*)-(3,7-dimethyloctoxy)}phenyl]porphyrinato-zinc(II), 11: A solution of 9 (0.20 g, 0.81 mmol) in chloroform (20 mL) was treated with TBAF (tetra-*n*-butylammonium fluoride, 1.0 M in THF; 1 mL, 1.0 mmol) for 15 min. To the mixture was added MeOH, brine, and chloroform. The organic layer separated was dried over anhydrous magnesium sulfate. The crude material was immediately used in the following synthetic step without further purification, otherwise the product 10 was decomposed even under ambient conditions.

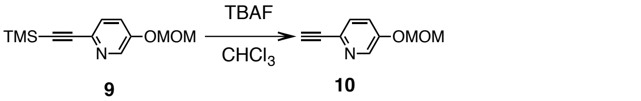

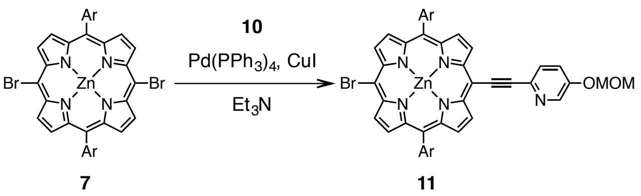

A mixture of the porphyrin **7** (2.5 g, 1.6 mmol), **10** (crude material), $Pd(PPh_3)_4$ (47 mg, 40 μmol) and CuI (7.7 mg, 40 μmol) in triethylamine (20 mL) was deaeratetd by successive freeze–pump–thaw cycles in a Schlenk flask, before purging with argon. The mixture was stirred at 50 °C for 1.5 h. The mixture diluted with chloroform (50 mL × 3) was washed with brine, and the organic layer was dried over anhydrous magnesium sulfate. The crude mixture was subjected to chromatographic separation over silica gel (pretreated with Et_3N) with *n*-hexane/ethyl acetate (8/1, v/v) as the eluent. The product was further purified by preparative GPC with chloroform as the eluent. The porphyrin **11** was obtained as a bluish green substance (0.50 g, 0.29 mmol) in 37% yield over 2 steps from **9**. The unreacted porphyrin **7** (2.0 g, 1.2 mmol) was recovered. ^{1}H NMR (300 MHz, in $CDCl_3$): δ 9.72 (d, *J* = 4.3 Hz, 2H; porphyrin-β), 9.02 (d, *J* = 4.3 Hz, 2H; porphyrin-β), 8.95 (d, *J* = 4.3 Hz, 2H; porphyrin-β), 8.56 (brs, 2H; porphyrin-β), 7.51 (s, 4H; Ar), 7.4–7.3 (brd, 1H; pyridyl), 6.45 (brd, *J* = 8.4 Hz, 1H; pyridyl), 6.36 (brd, *J* = 8.1 Hz, 1H; pyridyl), 4.56 (s, 2H; -PyOCH_2OMe), 4.43–4.39 (m, 4H; *p*-ArOCH_2-), 4.25 (m, 8H; *m*-ArOCH_2-), 3.21 (s, 3H; -OCH$_2$O*Me*), 2.3–0.7 (m, 114H; aliphatic chains) (At the concentrations around 10^{-3} M in $CDCl_3$, the monomeric species coexisted with the dimeric species assembled by self-complementary pyridyl-to-zinc coordination. Therefore, the signals of the protons of the pyridyl group were broadened and shifted into the upfield region); ^{13}C NMR (75 MHz, in $CDCl_3$): δ 153.7, 151.5, 151.3, 150.7, 149.6, 138.1, 137.9, 133.1, 122.6, 114.9, 106.5, 94.2, 72.1, 67.9, 56.3, 39.7, 39.4, 38.0, 37.7, 37.6, 36.8, 30.1, 30.0, 28.3, 28.1, 25.1, 24.9, 23.0, 22.9, 22.8, 22.7, 20.0, 19.9, 19.80. MALDI–TOF MS (dithranol): *m/z* calcd for $C_{101}H_{146}BrN_5O_8Zn$: 1699.96; found 1701.03 $[M+H]^+$. λ_{max}/nm ($\varepsilon/\mu M^{-1}cm^{-1}$ at 5.3×10^{-6} M in toluene with 1% pyridine) 447 (0.44), 585 (0.02), 638 (0.04).

1.10. Synthesis of 5-(triisopropylsilylethynyl)-15-{5-(methoxymethoxy)pyrid-2-yl}ethynyl-10,20-bis[3,4,5-tri{(3*S*)-(3,7-dimethyloctoxy)}phenyl]porphyrinatozinc(II), 1_1: A mixture of the porphyrin 11 (0.50 g, 0.30

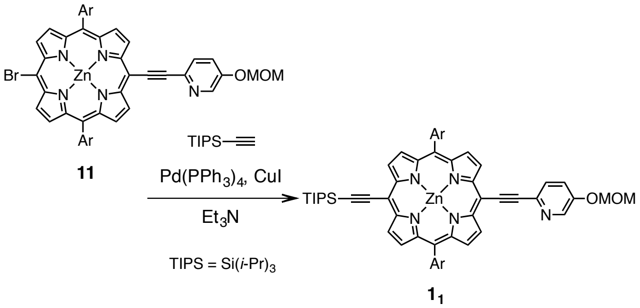

mmol), triisopropylsilylacetylene (330 μL, 1.5 mmol), $Pd(PPh_3)_4$ (17 mg, 15 μmol) and CuI (2.9 mg, 15 μmol) in Et_3N (10 mL) was deaeratetd by successive freeze–pump–thaw cycles in a Schlenk flask before purging with argon. The mixture was stirred at 50 °C for 16 h. The mixture diluted with chloroform was washed with brine, and the organic layer separated was dried over anhydrous sodium sulfate. The crude product was eluted from Et_3N-pretreated silica gel with *n*-hexane/ethyl acetate (gradient from 10/1 to 5/1, v/v) as the eluent. The monomeric porphyrin 1_1 was obtained as bluish green substance (0.38 g, 0.21 mmol) in 72% yield. ^{1}H NMR (300 MHz, in pyridine-d_5): δ 10.29 (d, J = 4.8 Hz, 2H; porphyrin-β), 10.19 (d, J = 4.5 Hz, 2H; porphyrin-β), 9.45 (d, J = 4.8 Hz, 2H; porphyrin-β), 9.37 (d, J = 4.5 Hz, 2H; porphyrin-β), 8.99 (d, J = 3.0 Hz, 1H; pyridyl), 8.21 (d, J = 8.7 Hz, 1H; pyridyl), 7.90 (s, 4H; Ar), 7.77 (dd, J = 8.7, 3.0 Hz, 1H; pyridyl), 5.42 (s, 2H; PyOCH_2OMe), 4.84–4.70 (m, 4H; *p*-ArOCH_2-), 4.35–4.34 (m, 8H; *m*-ArOCH_2-), 3.51 (s, 3H; PyOCH$_2$O*Me*), 2.4–0.8 (m, 135H; aliphatic chain *i*-Pr); ^{13}C NMR (75 MHz, in $CDCl_3$): δ 151.7, 151.4, 150.1, 150.0, 149.7, 149.5, 149.4, 136.8, 132.6, 131.6, 131.1, 130.8, 129.7, 127.1, 122.9, 121.6, 113.4, 109.0, 100.3, 99.4, 96.2, 92.9, 92.5, 70.9, 66.7, 66.5, 55.1, 38.5, 38.4, 38.19, 38.17, 36.8, 36.7, 36.5, 36.4, 36.3, 35.6, 28.9, 28.8, 27.1, 26.9, 23.9, 23.7, 21.8, 21.7, 21.6, 21.5, 18.8, 18.69, 18.65, 18.62, 18.57, 18.1, 10.9, 0.0. MALDI–TOF MS (dithranol): *m/z* calcd for $C_{112}H_{167}N_5O_8SiZn$: 1802.19; found 1803.23 $[M+H]^+$. λ_{max}/nm ($\varepsilon/\mu M^{-1}cm^{-1}$ at 9.7 × 10^{-7} M in toluene) 448 (0.39), 580 (0.01), 633 (0.04).

1.11. Synthesis of 5,15-bis(triisopropylsilylethynyl)-10,20-bis[3,4,5-tri{(3*S*)-(3,7-dimethyloctoxy)}phenyl]porphyrinatozinc(II), 2_{Si}, as the model porphyrin:

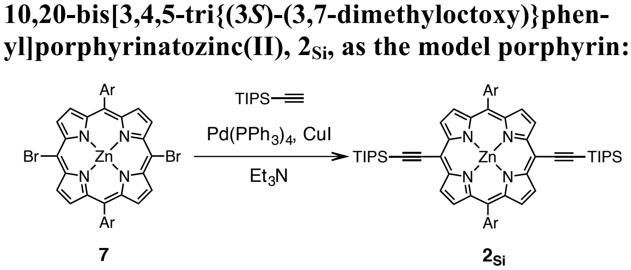

A solution of the porphyrin **7** (0.16 g, 1.0 mmol), triisopropylsilylacetylene (0.81 g, 4.4 mmol), $Pd(PPh_3)_4$ (6 mg, 5 μmol), CuI (1.9 mg, 10 μmol) in Et_3N (3 mL) was deoxygenated by successive freeze–pump–thaw cycles in a Schlenk flask, before purging with argon. The mixture was stirred at 50 °C for 14 h. The solvent was removed under the reduced pressure. The residue dissolved in toluene (100 mL) was successively washed with water and brine. The organic layer separated was dried over anhydrous magnesium sulfate. The target product was eluted from silica gel with toluene as the eluent. The porphyrin $\mathbf{2_{Si}}$ was obtained as bluish green substance (0.17 g, 0.93 mmol) in 94% yield. ^{1}H NMR (300 MHz, in $CDCl_3$): δ 9.76 (d, J = 4.7 Hz, 4H; porphyrin-β), 9.04 (d, J = 4.7 Hz, 4H; porphyrin-β), 7.38 (s, 4H; Ar), 4.33–4.26 (m, 4H; *p*-ArOCH_2-), 4.15–4.10 (m, 8H; *m*-ArOCH_2-), 2.2–0.8 (m, 156H; aliphatic chains and *i*-Pr of TIPS group); ^{13}C NMR (75 MHz, in $CDCl_3$): δ 152.5, 151.0, 150.2, 137.7, 137.1, 132.9, 131.2, 128.9, 128.1, 122.7, 114.0, 109.3, 101.8, 98.6, 71.9, 67.5, 39.5, 39.2, 37.7, 37.5, 37.3, 36.4, 30.1, 29.8, 28.1, 27.9, 27.7, 24.9, 24.7, 22.8, 22.73, 22.69, 22.6, 19.8, 19.7, 19.6, 19.3, 19.1, 18.8, 12.3, 11.9, 11.5, 1.1. MALDI–TOF MS (dithranol): *m/z* calcd for $C_{114}H_{180}N_4O_6Si_2Zn$: 1821.27; found 1822.34 $[M+H]^+$. λ_{max}/nm ($\varepsilon/\mu M^{-1}cm^{-1}$ in toluene) 443 (0.39), 580 (0.01), 627 (0.03).

1.12. Synthesis of 5,15-bis[(4-cyanophenyl)ethynyl]-10,20-bis[3,4,5-tri{(3*S*)-(3,7-dimethyloctoxy)}phenyl]porphyrinatozinc(II), 2_{Ar}:

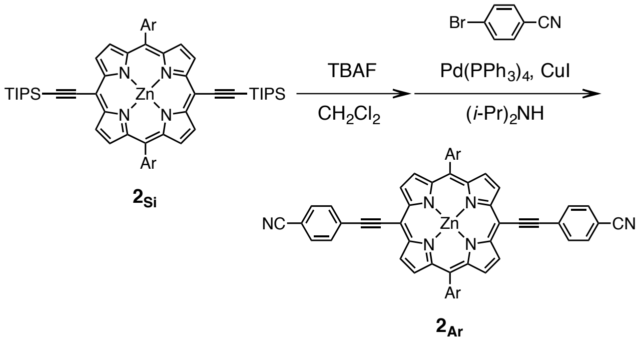

A solution of $\mathbf{2_{Si}}$ (122 mg, 66 μmol) in THF (5 mL) was treated with TBAF (1.0 M in THF; 0.1 mL, 0.1 mmol) for 15 min. To the mixture was added brine and chloroform. The organic layer separated was dried over anhydrous magnesium sulfate. The target material was passed through short silica gel plug with *n*-hexane/ethyl acetate (10/1, v/v) as the eluent. The product was used for the following without further purification. ^{1}H NMR (300 MHz, in $CDCl_3$): δ 9.73 (d, J = 4.8 Hz, 4H; porphyrin-β), 9.06 (d, J = 4.8 Hz, 4H; porphyrin-β), 7.38 (s, 4H; Ar), 4.29 (q, J = 6.1 Hz, 4H; *p*-ArOCH_2-), 4.12 (s, 2H; acetylene), 4.10 (brm, 8H; *m*-ArOCH_2-), 2.1–0.8 (m, 114H; aliphatic chains).

A solution of the desilylated porphyrin, 4-bromobenzonitrile (0.15 g, 0.82 mmol), $Pd(PPh_3)_4$ (6 mg, 5 μmol), CuI (1.9 mg, 10 μmol) in THF (3 mL) and (i-Pr)$_2$NH (5 mL) was gently refluxed under inert atmosphere for 14 h. To the mixture was added brine and tol-

uene. The organic layer separated was dried over anhydrous magnesium sulfate. The target material was passed through short silica gel plug with toluene as the eluent, followed by size–exclusion chromatography (BioBeads® S-X1) with toluene as the eluent. The porphyrin $\mathbf{2_{Ar}}$ precipitated from methanol was obtained as greenish blue solid (82 mg, 48 μmol) in 73% yield over two steps. ^{1}H NMR (300 MHz, in $CDCl_3$): δ 9.64 (d, J = 4.2 Hz, 4H; porphyrin-β), 9.07 (d, J = 4.2 Hz, 4H; porphyrin-β), 7.98 (d, J = 8.1 Hz, 4H; 4-cyanophenyl), 7.75 (d, J = 8.1 Hz, 4H; 4-cyanophenyl), 7.40 (s, 4H; Ar), 4.27–4.21 (m, 4H; *p*-ArOCH_2-), 4.12 (brm, 8H; *m*-ArOCH_2-), 2.1–0.8 (m, 114H; aliphatic chains). ^{13}C NMR (75 MHz, in $CDCl_3$): δ 152.0, 151.2, 150.5, 138.0, 136.8, 133.2, 132.3, 131.8, 130.8, 128,7, 123.6, 118.5, 114.3, 111.4, 100.4, 97.0, 95.1, 72.0, 67.7, 39.5, 39.2, 37.7, 37.6, 37.4, 36.5, 29.9, 28.1, 27.9, 24.9, 24.7, 22.8, 22.72, 22.68, 22.6, 19.8, 19.6. MALDI–TOF MS (dithranol): *m/z* calcd for $C_{110}H_{146}N_6O_6Zn$: 1711.06; found 1711.81 $[M+H]^+$.

1.13. Synthesis of 5-bromo-15-(triisopropylsilylethynyl)-10,20-bis[3,4,5-tri{(3*S*)-(3,7-dimethyloctoxy)}phenyl]porphyrinatozinc(II), 12:

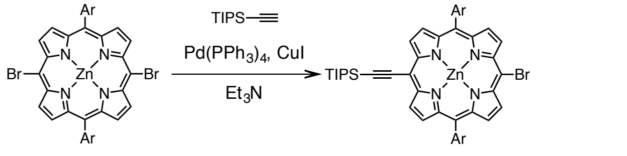

7 **12**

The porphyrin **13** was prepared by stepwise introduction of triisopropylsilylacetylene and then trimethylsilylacetylene from **7**, since the one-pot Sonogashira–Hagihara coupling of **7** with triisopropylsilylacetylene together with trimethylsilylacetylene mainly furnished the symmetrically coupled product with two TMS terminals presumably due to the higher reactivity of trimethylsilylacetylene.

A solution of the porphyrin **7** (0.50 g, 0.31 mmol), triisopropylsilylacetylene (56 mg, 0.31 mmol), $Pd(PPh_3)_4$ (18 mg, 15 μmol), CuI (5.8 mg, 31 μmol) in Et_3N (10 mL) was deaeratetd by successive freeze–pump–thaw cycles in a Schlenk flask, before purging with argon. The mixture was stirred at 50 °C for 16 h. The reaction mixture was diluted with chloroform and washed with water. The organic layer separated was dried over anhydrous magnesium sulfate. The crude material was eluted from silica gel column chromatography with *n*-hexane/ethyl acetate (10/1, v/v) as the eluent, followed by purification by preparative GPC with chloroform as the eluent. The target porphyrin **12** was obtained as bluish green substance (0.14 g, 81 μmol) in 26% yield. The unconsumed porphyrin **7** (84 mg, 52 μmol) was recovered in 17% yield, and the porphyrin **2** (0.20 g, 0.11 mmol) was also obtained in 35% yield. The porphyrin **12:** ^{1}H NMR (300 MHz, in $CDCl_3$): δ 9.75 (d, J = 4.5 Hz, 2H; porphyrin-β), 9.70 (d, J = 4.8 Hz, 2H; porphyrin-β), 9.05 (d, J = 4.5 Hz, 2H; porphyrin-β), 9.01 (d, J = 4.8 Hz, 2H; porphyrin-β), 7.37 (s, 4H; Ar), 4.33–4.26 (*p*-ArOCH_2-), 4.14–4.10 (m, 8H; *m*-ArOCH_2-), 2.1–0.8 (m, 135H; aliphatic chains and *i*-Pr); ^{13}C NMR (75 MHz, in $CDCl_3$): δ 153.3, 152.5, 151.0, 150.3, 149.6, 137.8, 137.1, 133.4, 133.2, 133.0, 131.4, 131.2, 122.6, 114.2, 106.3, 101.0, 98.4, 77.2, 72.0, 67.6, 39.5, 39.3, 37.7, 37.5, 37.4, 36.5, 31.6, 29.9, 28.1, 28.0, 24.9, 24.7, 22.8, 22.72, 22.68, 22.6, 19.8, 19.7, 19.1, 14.1, 11.9. MALDI–TOF MS (dithranol): *m/z* calcd for $C_{103}H_{159}BrN_4O_6SiZn$: 1719.05; found 1720.04 $[M+H]^+$. λ_{max}/nm ($\varepsilon/\mu M^{-1}cm^{-1}$ at 3.3 × 10^{-6} M in toluene) 439 (0.31), 570 (0.01), 613 (0.01), 625 (0.01).

1.14. Synthesis of 5-(triisopropylsilylethynyl)-15-(trimethylsilylethynyl)-10,20-bis[3,4,5-tri{(3*S*)-(3,7-dimethyloctoxy)}phenyl]porphyrinatozinc(II), 13:

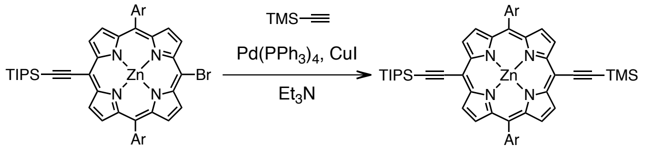

12 **13**

A solution of the porphyrin **12** (0.14 g, 80 μmol), trimethylsilylacetylene (16 mg, 0.16 mmol), $Pd(PPh_3)_4$ (4.6 mg, 4.0 μmol), CuI (2.0 mg, 10 μmol) in Et_3N (10 mL) was deaeratetd by successive freeze–pump–thaw cycles in a Schlenk flask, before purging with argon. The mixture was stirred at 50 °C for 3 h. The reaction mixture was diluted with chloroform and washed with water. The organic layer separated was dried over anhydrous magnesium sulfate. The crude material was eluted from silica gel column chromatography with *n*-hexane/ethyl acetate (10/1, v/v) as the eluent. The target porphyrin **13** was obtained as bluish green substance (0.14 g, 80 μmol) in quantitative yield. ^{1}H NMR (300 MHz, in $CDCl_3$): δ 9.75 (d, J = 4.5 Hz, 2H; porphyrin-β), 9.70 (d, J = 4.5 Hz, 2H; porphyrin-β), 9.05–9.01 (m, 4H; porphyrin-β), 7.39 (s, 4H; Ar), 4.35–4.28 (*p*-ArOCH_2-), 4.13 (brs, 8H; *m*-ArOCH_2-), 2.1–0.8 (m, 135H; aliphatic chains and *i*-Pr), 0.60 (s, 9H; TMS); ^{13}C NMR (75 MHz, in $CDCl_3$): δ 152.9, 152.8, 151.4, 150.71, 150.68, 137.9, 137.7, 133.3, 131.6, 123.1, 114.5, 110.0, 102.3, 102.1, 101.7, 98.8, 77.7, 72.3, 67.9, 40.0, 39.7, 38.2, 37.9, 37.8, 36.9, 30.3, 28.6, 28.5, 25.4, 25.2, 23.3, 23.2, 23.1, 20.2, 20.1, 19.6, 12.4, 1.6, 1.1, 0.9. MALDI–TOF MS (dithranol): *m/z* calcd for $C_{108}H_{168}N_4O_6Si_2Zn$: 1737.18; found 1738.13 $[M+H]^+$. λ_{max}/nm ($\varepsilon/\mu M^{-1}cm^{-1}$ at 3.2 × 10^{-6} M in toluene) 443 (0.34), 579 (0.01), 625 (0.02), 642 (0.02).

1.15. Synthesis of 5-[(5-bromopyrid-2-yl)ethynyl]-15-(triisopropylsilylethynyl)-10,20-bis[3,4,5-tri{(3*S*)-(3,7-dimethyloctoxy)}phenyl]porphyrinatozinc(II), 15:

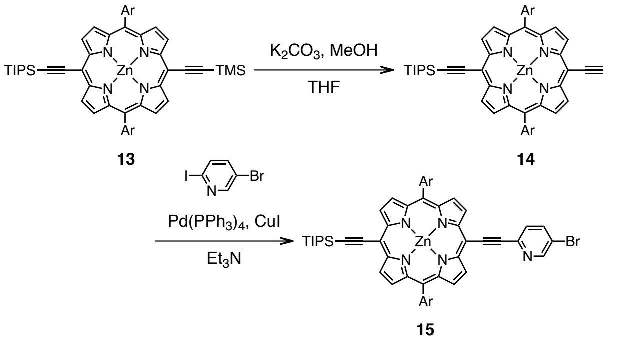

The terminal TMS group of the porphyrin **13** was selectively deprotected by methanolysis. A solution of the porphyrin **13** (61 mg, 35 μmol) in THF (5 mL) and MeOH (10 mL) was stirred with potassium carbonate (9.5 mg, 69 μmol) at room temperature for 3 h. The mixture was diluted with chloroform, and washed with water. The organic layer separated was dried over anhydrous magnesium sulfate. The target material **14** was eluted from silica gel column chromatography with *n*-hexane/ethyl acetate (10/1, v/v) as the eluent. The product **14** was quantitatively obtained, which was used for the following step immediately after purification. The porphyrin **14:** ^{1}H NMR (300 MHz, in $CDCl_3$): δ 9.77 (d, J = 4.8 Hz, 2H; porphyrin-β), 9.73 (d, J = 4.6 Hz, 2H; porphyrin-β), 9.06 (d, J = 4.8 Hz, 2H; porphyrin-β), 9.05 (d, J = 4.6 Hz, 2H; porphyrin-β), 7.39 (s, 4H; Ar), 4.34–4.27 (m, 4H; *p*-ArOCH_2-), 4.18–4.10 (m, 8H; *m*-ArOCH_2-), 2.1–0.8 (m, 156H; aliphatic chains and *i*-Pr).

A solution of the porphyrin **14** (58 mg, 35 μmol), 5-bromo-2-iodopyridine (16 mg, 0.16 mmol), $Pd(PPh_3)_4$ (2 mg, 1 μmol), CuI (1 mg, 4 μmol) in Et_3N (5 mL) was deaeratetd by successive freeze–pump–thaw cycles in a Schlenk flask, before purging with argon. The mixture was stirred at 50 °C for 18 h. The reaction mixture was diluted with chloroform and washed with brine. The organic layer separated was dried over anhydrous magnesium sulfate. The crude material was eluted from silica gel column chromatography with *n*-hexane/ethyl acetate (10/1, v/v) as the eluent. The target porphyrin **15** (55 mg, 30 μmol) was obtained as bluish green substance in 86% yield. ^{1}H NMR (300 MHz, in $CDCl_3$): δ 9.77 (d, J = 4.8 Hz, 2H; porphyrin-β), 9.22 (d, J = 2.4 Hz, 2H; porphyrin-β), 9.0 (d, J = 4.8 Hz, 2H; porphyrin-β), 7.52 (brd, J = 7.8 Hz, 1H; pyridyl), 7.40 (Ar, s, 4H; Ar), 7.22–7.21 (br, 1H; pyridyl), 6.50 (brs, 1H; pyridyl), 4.36–4.30 (m, 4H; *p*-ArOCH_2-), 4.15 (brs, 8H; *m*-ArOCH_2-), 2.2–0.8 (m, 135H; aliphatic chains and *i*-Pr) (At the concentrations around 10^{-3} M in $CDCl_3$, the dimeric species was dominant, as the signals of pyridyl group broadened and shifted into the upfield region); ^{13}C NMR (75 MHz, in $CDCl_3$): δ 152.6, 152.4, 151.13, 151.08, 150.7, 150.4, 148.9, 139.5, 138.3, 138.0, 137.5, 132.7, 131.4, 131.2, 127.7, 122.9, 117.8, 114.6, 109.7, 102.3, 99.2, 98.2, 95.9, 89.5, 72.0, 67.8, 67.7, 39.6, 39.28, 39.26, 37.8, 37.7, 37.5, 37.4, 36.6, 30.0, 29.9, 28.1, 28.0, 25.0, 24.8, 22.9, 22.8, 22.7, 22.6, 19.83, 19.77, 19.74, 19.70, 19.66, 19.2, 12.0. MALDI–TOF MS (dithranol): *m/z* calcd for $C_{110}H_{162}BrN_5O_6SiZn$: 1820.08; found 1820.96 $[M+H]^+$. λ_{max}/nm ($\varepsilon/\mu M^{-1}cm^{-1}$ at 1.7×10^{-6} M in toluene) 451 (0.38), 602 (0.01), 655 (0.05).

1.16. Synthesis of dimeric porphyrin array 1_2:

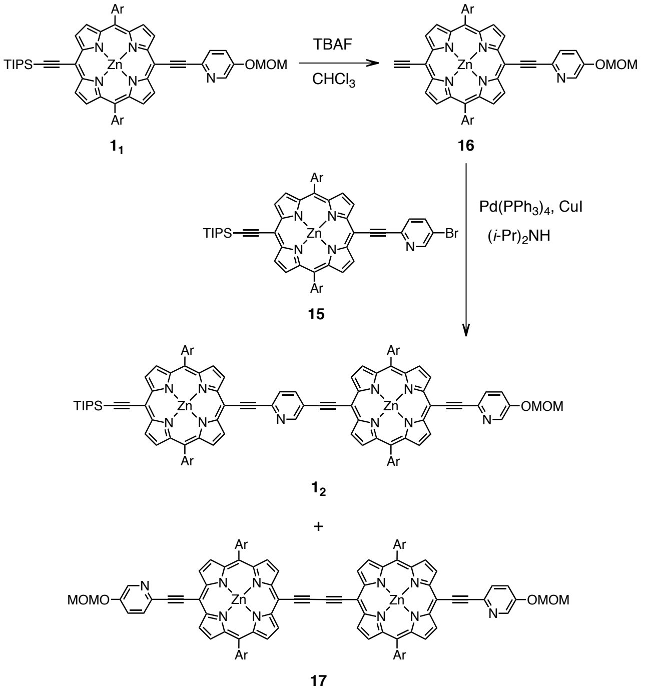

A solution of $\mathbf{1_1}$ (0.16 g, 89 μmol) in THF (5 mL) was treated with TBAF (1.0 M in THF; 0.1 mL, 0.1 mmol) for 15 min. To the mixture was added brine and chloroform. The organic layer separated was dried over anhydrous magnesium sulfate. The target material **16** was passed through short silica gel plug with *n*-hexane/ethyl acetate (10/1, v/v) as the eluent. The product **16** was used for the following step immediately after purification.

A solution of the porphyrins **14** (0.17 g, 91 μmol), **16** (0.15 g, 88 μmol), $Pd(PPh_3)_4$ (5.2 mg, 4.5 μmol), and CuI (1 mg, 4 μmol) in $(i\text{-Pr})_2NH$ (10 mL) was deaeratetd by successive freeze–pump–thaw cycles in a Schlenk flask, before purging with argon. The mixture was stirred at 50 °C for 60 h. The reaction mixture was diluted with chloroform and washed with brine. The organic layer separated was dried over anhydrous magnesium sulfate. The crude material was eluted from Et_3N-pretreated silica gel column chromatography with *n*-hexane/ethyl acetate (15/1, v/v) as the eluent. The target porphyrin was isolated by successive size-exclusion chromatography (Bio-Rad Laboratories, BioBeads® S-X1) with toluene and toluene/pyridine (9/1, v/v) as the eluent. The target porphyrin $\mathbf{1_2}$ (0.20 g, 60 μmol) was obtained as bluish green substance in 69% yield. The excess porphyrin **15** was recovered in 19% yield (32 mg, 17 μmol). The dimeric porphyrin array $\mathbf{1_2}$: ^{1}H NMR (300 MHz, in toluene-d_8): δ 10.16 (d, J = 4.5 Hz, 2H;

porphyrin-β), 9.93 (brd, 2H; porphyrin-β), 9.55 (brd, J = 4.2 Hz, 2H; porphyrin-β), 9.46 (brd, J = 3.6 Hz, 2H; porphyrin-β), 9.44 (brd, J = 3.6 Hz, 2H; porphyrin-β), 9.27 (brd, J = 4.2 Hz, 2H; porphyrin-β), 9.09 (d, J = 4.5 Hz, 2H; porphyrin-β), 9.05 (d, J = 4.2 Hz, 2H; porphyrin-β), 8.14 (brs, 2H; Ar), 8.1–7.2 (brs, 4H; Ar), 7.48 (brs, 2H; Ar), 6.09 (d, J = 7.8 Hz, 1H; pyridyl), 5.69 (d, J = 7.8 Hz, 1H; pyridyl), 5.59 (d, J = 8.7 Hz, 1H; pyridyl), 5.38 (d, J = 8.7 Hz, 1H; pyridyl), 4.64–4.62 (m, 8H, p-ArOCH_2-), 4.23 (s, 2H; PyOCH_2OMe), 4.2–3.8 (brm, 16H, m-ArOCH_2-), 3.62 (s, 1H; pyridyl), 2.98 (s, 3H; PyOCH$_2$OMe), 2.59 (s, 1H; pyridyl), 2.5–0.7 (m, 249H; aliphatic chains and i-Pr); ^{13}C NMR (75 MHz, in CDCl$_3$): δ 153.0, 152.9, 152.4, 152.1, 151.4, 151.3, 150.9, 150.8, 150.5, 138.2, 138.1, 137.9, 137.8, 132.9, 132.6, 132.1, 130.9, 128.1, 123.1, 123.0, 114.7, 94.2, 72.1, 67.9, 56.3, 39.7, 39.6, 39.3, 38.0, 37.9, 37.6, 37.5, 36.8, 30.1, 30.0, 28.2, 27.99, 27.96, 25.0, 24.8, 22.9, 22.8, 22.73, 22.71, 22.6, 19.9, 19.8, 19.7, 19.2, 12.0, 1.1. MALDI–TOF MS (dithranol): m/z calcd for $C_{213}H_{308}N_{10}O_{14}SiZn_2$: 3386.20; found 3387.359 $[M+H]^+$ and 6772.55 $[M_2 + H]^+$. λ_{max}/nm ($\varepsilon/\mu M^{-1}cm^{-1}$ at 10^{-7}–10^{-4} M in toluene) 443 (0.33), 461 (0.33), 589 (0.02), 670 (0.10).

In this reaction conditions for the Sonogashira-Hagihara coupling, the Glaser coupling also proceeded as the side reaction even though the reaction mixture was thoroughly deoxygenated. Thus, the precursor **16** was consumed by this side reaction, and the brown compound **17** was isolated. ^{1}H NMR (300 MHz, in pyridine-d_5): δ 10.33 (d, J = 4.5 Hz, 8H; porphyrin-β), 9.54 (d, J = 4.5 Hz, 4H; porphyrin-β), 9.42 (d, J = 4.5 Hz, 4H; porphyrin-β), 8.99 (d, J = 2.8 Hz, 2H; pyridyl), 8.22 (d, J = 8.7 Hz, 2H; pyridyl), 7.91 (s, 8H; Ar), 7.77 (dd, J = 8.7, 2.8 Hz, 2H; pyridyl), 5.42 (s, 4H; PyOCH_2OMe), 4.45–4.65 (m, 8H; p-ArOCH_2-), 4.34–4.33 (br, 16H; m-ArOCH_2-), 3.50 (s, 6H; PyOCH$_2$OMe), 2.4–0.8 (m, 228H; aliphatic chains). ^{13}C NMR (75 MHz, in CDCl$_3$): δ 155.8, 155.6, 154.8, 154.0, 153.3, 153.0, 143.0, 140.1, 140.0, 139.7, 136.0, 135.6, 133.7, 133.3, 130.5, 126.5, 126.1, 117.0, 103.9, 101.9, 99.5, 97.0, 94.0, 92.5, 85.4, 74.1, 69.8, 58.3, 41.7, 41.5, 40.2, 40.0, 39.7, 39.0, 32.3, 32.2, 30.3, 30.2, 27.3, 27.1, 25.0, 24.9, 24.8, 22.1, 21.8, 21.3. MALDI-TOF MS (dithranol): m/z calcd for $C_{206}H_{292}N_{10}O_{16}Zn_2$: 3290.09; found 3291.194 $[M+H]^+$.

1.17. Synthesis of trimeric porphyrin array $\mathbf{1_3}$:

A solution of $\mathbf{1_2}$ (65 mg, 19 μmol) in THF (5 mL) was treated with TBAF (1.0 M in THF; 20 μL, 20 μmol) for 15 min. The mixture was diluted with chloroform, and washed with brine. The organic layer separated was dried over anhydrous magnesium sulfate. The target material **17** was eluted from short silica gel plug with n-hexane/ethyl acetate (10/1, v/v) as the eluent. This material was used for the following step immediately after purification.

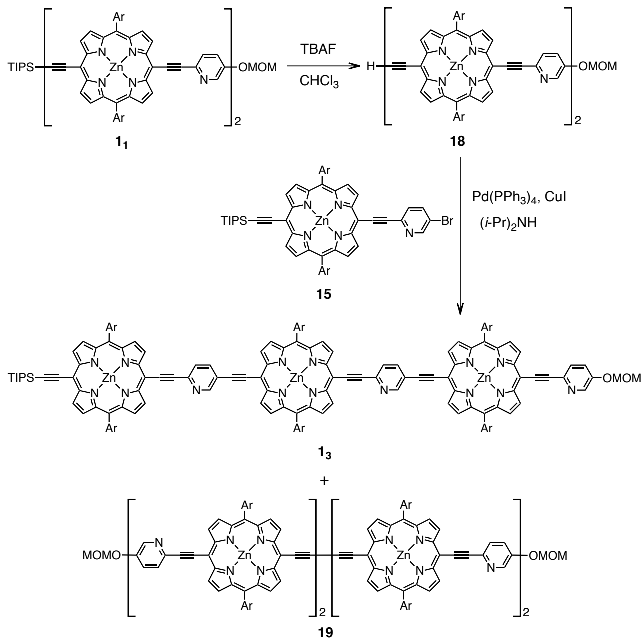

A solution of the porphyrins **15** (85 mg, 47 μmol), **17** (61 mg, 19 μmol), Pd(PPh$_3$)$_4$ (1 mg, 1 μmol), CuI (1 mg, 4 μmol) in (i-Pr)$_2$NH (10 mL) was deaeratetd by successive freeze–pump–thaw cycles in a Schlenk flask, before purging with argon. The mixture was stirred at 40 °C for 40 h. The reaction mixture was diluted with chloroform and washed with brine. The organic layer separated was dried over anhydrous magnesium sulfate. The crude material was eluted from Et$_3$N-pretreated silica gel column chromatography with n-hexane/ethyl acetate (10/1, v/v) as the eluent. The target porphyrin was isolated by successive size-exclusion chromatography (BioBeads® S-X1) with toluene, toluene/pyridine (4/1, v/v) and pyridine as the eluent. The target porphyrin $\mathbf{1_3}$ (23 mg, 4.6 μmol) was obtained as bluish green substance in 24% yield. The excess porphyrin **15** was recovered in 79% (67 mg, 37 μmol). The trimeric porphyrin array $\mathbf{1_3}$: ^{1}NMR (300 MHz, in toluene-d_8): δ 10.16 (d, J = 4.5 Hz, 2H; porphyrin-β), 9.97–9.96 (brm, 4H; porphyrin-β), 9.57–9.46, (brm, 10H; porphyrin-β), 9.28–9.06 (brm, 8H; porphyrin-β), 8.20–8.13 (brm, 4H; Ar), 8.1–7.51 (brs, 4H; Ar), 7.51–7.48 (brm, 4H; Ar), 6.34 (brd, J = 7.5 Hz, 1H; pyridyl), 6.11 (brd, J = 8.4 Hz, 1H; pyridyl), 6.00 (brd, J = 6.0 Hz, 1H; pyridyl), 5.74 (d, J = 7.8 Hz, 1H; pyridyl), 5.59 (d, J = 8.7 Hz, 1H; pyridyl), 5.37 (d, J = 9.9 Hz, 1H; pyridyl), 4.8–4.5 (m, 12H, p-ArOCH_2-), 4.22 (s, 2H; PyOCH_2OMe), 4.1–3.7 (brm, 24H, m-ArOCH_2-), 3.84 (s, 1H; pyridyl), 3.48 (s, 1H; pyridyl), 3.00 (s, 3H; PyOCH$_2$OMe), 2.56 (s, 1H; pyridyl), 2.5–0.7 (m, 363H; aliphatic chains and i-Pr); ^{13}C NMR (75 MHz, in pyridine-d_5): δ 154.6, 154.4, 154.3, 153.70, 153.65, 152.9, 152.8, 152.74, 152.68, 152,2, 150.7, 140.34, 140.26, 139.8, 137.8, 136.5, 135.3, 133.3, 130.2, 126.2, 125.86, 125.78, 124.4, 116.7, 96.5, 73.8, 69.5, 57.8, 41.3, 41.1, 39.9, 39.7, 39.3, 38.6, 32.0, 31.8, 31.6, 30.0, 29.8, 26.9, 26.7, 24.6, 24.5, 24.4, 21.8, 21.4, 20.9, 13.8, 3.0. MALDI–TOF MS (dithranol): m/z calcd for

$C_{314}H_{449}N_{15}O_{20}SiZn_3$: 4970.22; found 4972.51 $[M+H]^+$ and 9954.43 $[M_2 + H]^+$. λ_{max}/nm ($\varepsilon/\mu M^{-1}cm^{-1}$ at 10^{-7}–10^{-4} M in toluene) 443 (0.42), 468 (0.40), 589 (0.03), 676 (0.14).

In this reaction step, the Glaser coupling mainly gave the unexpected bluish black porphyrin **19** as the sole byproduct (47 mg, 7.2 μmol) in 76% yield. The compound is to be described elsewhere.[S5]

1.18. Synthesis of 2-(phenylethynyl)pyridine:

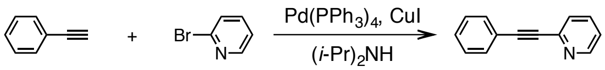

A solution of phenylacetylene (1.4 g, 14 mmol) in *i*-Pr_2NH (10 mL) was degassed by freeze-pump-thaw cycles. To the solution was added 2-bromopyridine (1.3 g, 8.0 mmol) and $Pd(PPh_3)_4$ (0.46 g, 0.40 mmol) and CuI (77 mg, 0.40 mmol) under argon atmosphere. Heating of the mixture at 50 °C only for ca. 5 sec provided precipitation. The mixture dissolved in toluene was washed with brine before drying over anhydrous magnesium sulfate. The product was purified by silica gel column chromatography with *n*-hexane/ethyl acetate (10/1, v/v) and then chloroform. The product was obtained as orange oil (1.3 g, 7.6 mmol) in 95% yield. ^{1}H and ^{13}C NMR spectra of the product agreed with the authentic profiles.[S6]

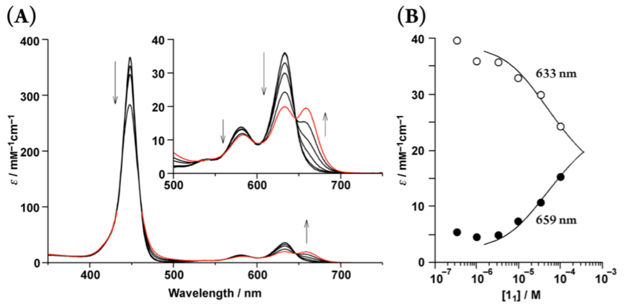

Figure S1. (A) Concentration variation (9.7×10^{-8}–3.3×10^{-4} M) of $\mathbf{1_1}$ at 25 °C in toluene. The inset magnifies the region of Q band. Red line shows the electronic absorption of $\mathbf{1_1}$ at 3.3×10^{-4} M omitted the Soret band because of spectral saturation. (B) Plot of extinction coefficient (ε) of $\mathbf{1_1}$ at 633 (open circle) and 659 (filled circle) nm against concentration. Solid lines represent theoretical curves, assuming $K_{ds}(1) = (1.3 \pm 0.2) \times 10^4$ M^{-1}.

2. Equilibrium Analyses

2.1. Dimer formation of $(\mathbf{1_1})_2$:

In the electronic absorption spectra (Figure S1A), $\mathbf{1_1}$ exhibited large spectral change depending on concentra-

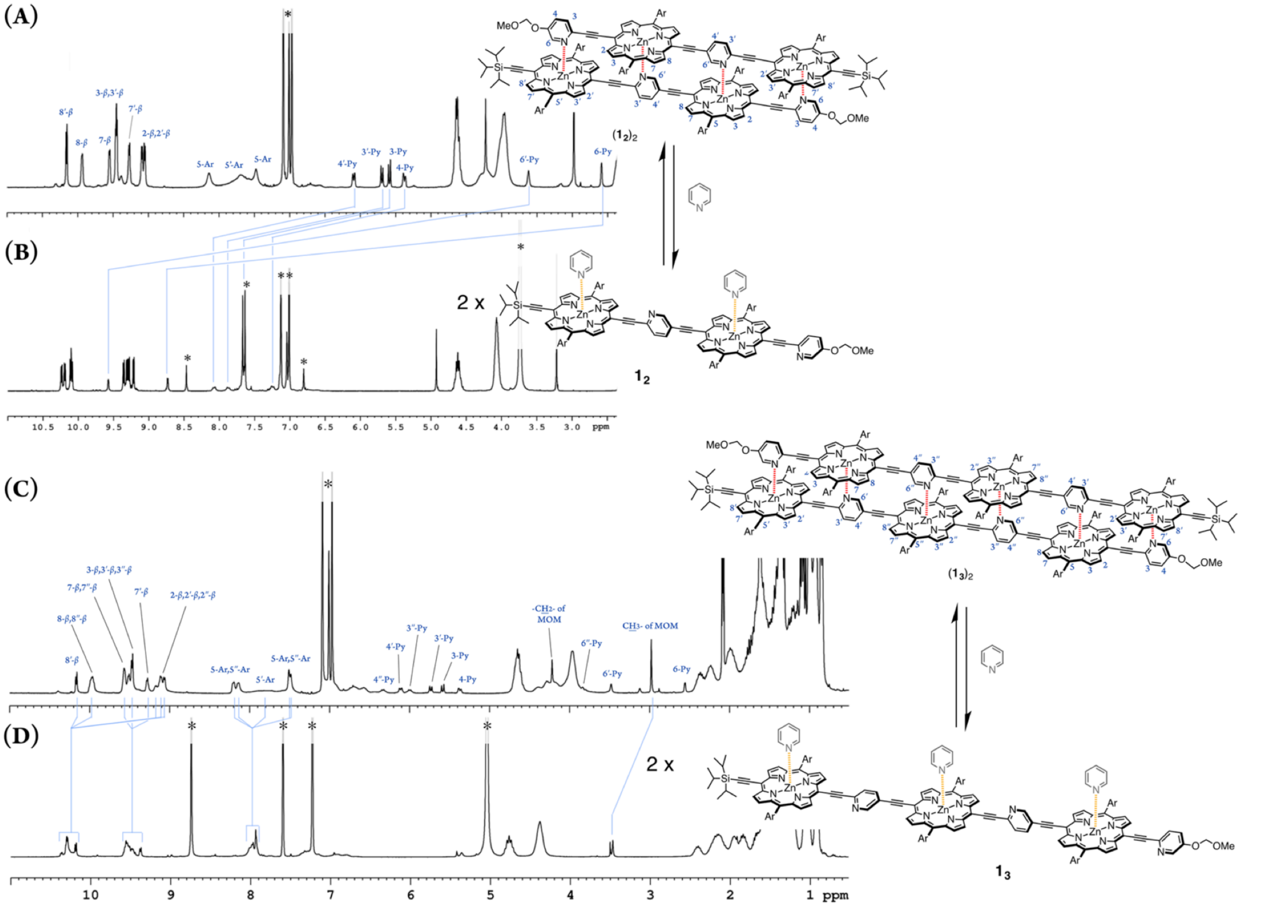

Figure S2. ^{1}H NMR spectra of $(\mathbf{1_2})_2$ in toluene-d_8 (A) and $\mathbf{1_2}$ in toluene-d_8 including 5% pyridine-d_5 (B). ^{1}H NMR spectra of $(\mathbf{1_3})_2$ in toluene-d_8 (C) and $\mathbf{1_3}$ in pyridine-d_5 (D). The asterisk indicates residual solvent and water.

tion variation (10^{-7}–10^{-4} M), unlike $\mathbf{1_2}$ and $\mathbf{1_3}$. The spectral change showed several isosbestic points in the course of concentration variation, indicating the two-state equilibrium between two stationary states, *i.e.*, monomeric $\mathbf{1_1}$ and double-strand $(\mathbf{1_1})_2$. Based on the spectral change, the association constants of $(\mathbf{1_1})_2$ ($K_{ds}(1)$) was estimated according to the following equation (Equation S1).

$$\varepsilon_{obs} = \varepsilon(\mathbf{1_1}) + \{K_{ds}(1)/(1 + K_{ds}(1))\}\cdot\{\varepsilon((\mathbf{1_1})_2) - \varepsilon(\mathbf{1_1})\} \quad \text{(S1)}$$

A nonlinear regression analysis gave $(1.3 \pm 0.2) \times 10^4$ M^{-1} of $K_{ds}(1)$ ($\Delta G_{ds}(1)° = -23 \pm 1$ kJ·mol^{-1}) at 25 °C in toluene (Figure S1B).

2.2. Competitive titration experiments of $(\mathbf{1_2})_2$ and $(\mathbf{1_3})_2$: The double-strands $(\mathbf{1_n})_2$ were readily unzipped by the addition of excess amount of pyridine as a competitive ligand. The ^{1}H NMR spectra of $(\mathbf{1_n})_2$ showed significant upfield shift of the protons of pyridyl groups, while the upfield shift disappeared in the presence of the pyridine-d_5 (Figure S2). The results indicated that self-complementary coordination bonds played crucial role in the formation of the double-strands $(\mathbf{1_n})_2$.

The double-strand $(\mathbf{1_2})_2$ obeyed the Beer's law at more than 10^{-7} M. In spectrometric titration experiments, we observed the spectral change along unzipping of the double-strand. The electronic absorption spectra of $(\mathbf{1_2})_2$ showed several pseudo-isosbestic points in the course of addition of pyridine (Figure S3). Similar unzipping behaviors were observed for $(\mathbf{1_3})_2$ (Figure S4). The entire unzipping equilibria were analyzed by employing the simplified model, although we did not draw conclusive model at this moment. In this model, we assumed that $2n$ ligands simultaneously bound to the double-strand $(\mathbf{1_n})_2$ and then two unzipped single-strands formed. Ac-

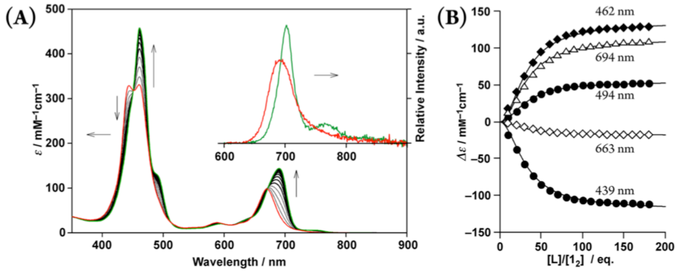

Figure S3. Spectrometric titration of $(\mathbf{1_2})_2$ ($[\mathbf{1_2}]_0 = 3.2 \times 10^{-6}$ M) with pyridine (up to 180 equiv., red to green) at 25 °C in toluene. The inset shows fluorescence spectra of $(\mathbf{1_2})_2$ and $\mathbf{1_2}$ with excess pyridine (red and green, respectively) in toluene ($\lambda_{ex} = 452$ nm, a qseudo-isosbestic point). (B) Plots of spectral change in the absorption spectra, shown in A, with the theoretical curves based on non-linear least-squares fitting assuming $K_{uz}(2) = (4.1 \pm 0.2) \times 10^8$ M^{-3}.

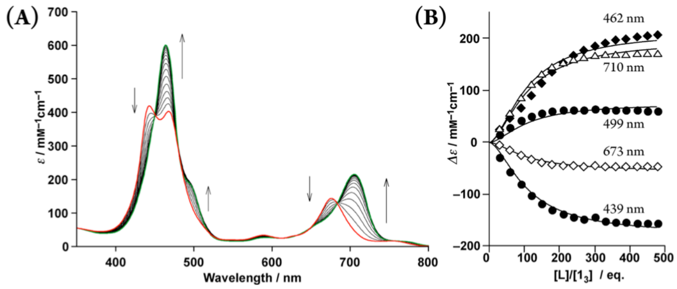

Figure S4. Spectrometric titration of $(\mathbf{1_3})_2$ ($[\mathbf{1_3}]_0 = 2.9 \times 10^{-6}$ M) with pyridine (up to 480 equiv., red to green) at 25 °C in toluene. (B) Plots of spectral change in the absorption spectra with the theoretical curves based on non-linear least-squares fitting assuming $K_{uz}(2) = (6.5 \pm 1.2) \times 10^{15}$ M^{-5}.

cording

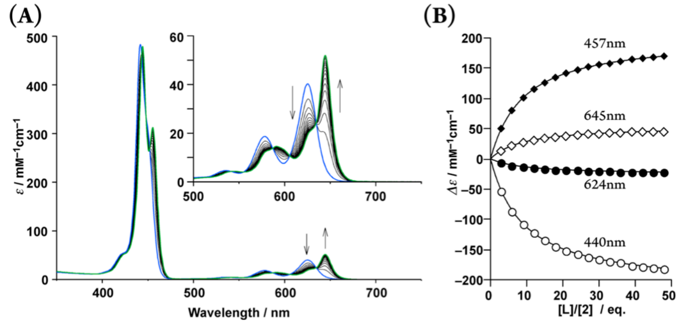

Figure S5. (A) Spectrometric titration of $\mathbf{2_{Si}}$ ($[\mathbf{2_{Si}}]_0 = 3.5 \times 10^{-6}$ M) with 2-(phenylethynyl)pyridine (up to 48 equiv., blue to green) at 25 °C in toluene. The inset magnifies the Q band. (B) Plot of extinction coefficient (ε) of $\mathbf{2_{Si}}$ at various wavelengths and theoretical curves (solid lines) assuming $K_\mu = (3.2 \pm 0.1) \times 10^4$ M^{-1}.

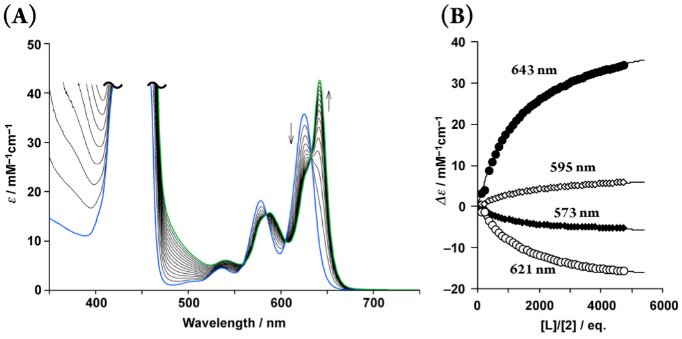

Figure S6. (A) Spectrometric titration of $\mathbf{2_{Si}}$ ($[\mathbf{2_{Si}}]_0 = 7.8 \times 10^{-5}$ M) with 2-(phenylethynyl)pyridine (up to 4700 equiv., blue to green) at 25 °C in toluene. The Soret bands exceeded the detection limit of our spectrometer. (B) Plot of extinction coefficient (ε) of $\mathbf{2_{Si}}$ and theoretical regression curve (solid line), assuming $K_\mu = (7.8 \pm 1.1)$ M^{-1}.

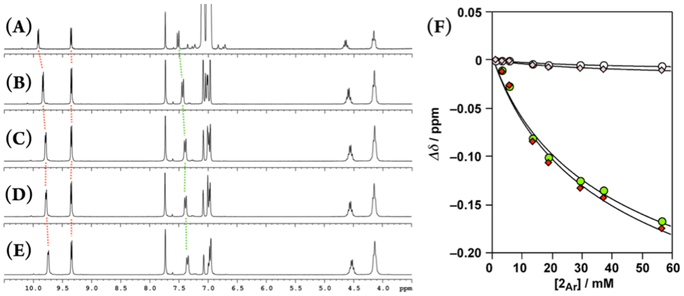

Figure S7. Representative NMR spectra of $\mathbf{2_{Ar}}$ at 1.0×10^{-3} M (A), 1.4×10^{-2} M (B), 2.9×10^{-2} M (C), 3.7×10^{-2} M (D), and 5.7×10^{-2} M (E). (F) Plot of the signals of the porphyrin-β protons (squares), 4-cyanophenyl protons (green circles), and trialkoxy aryl protons (open circles) and theoretical regression curve (solid lines), assuming K_μ = (11 ± 1) M^{-1}.

curate thermodynamic investigations of the double-strand $(\mathbf{1}_n)_2$ by employing isothermal titration carolimetry is currently underway.

2.3. Microscopic binding constant: The microscopic binding of pyridyl groups to zinc sites in the double-strands $(\mathbf{1}_n)_2$ is essential in the double-strand formation. However, it is difficult to directly estimate the microscopic binding constant (K_μ). We employed model compounds, *i.e.*, porphyrin **2** and 2-(phenylethynyl)pyridine, to estimate K_μ based on titration experiment (Figure S6). A nonlinear regression analyses determined the K_μ value to be (3.2 ± 0.1) × 10^4 M^{-1} and 7.8 ± 1.1 M^{-1} for pyridine and 2-(phenylethynyl)pyridine, respectively (Figure S5 and S6). The former was used for estimation of $K_{ds}(n)$.

To estimate reliable microscopic constant, we evaluated the strength of the π-stacked interaction by using model porphyrin $\mathbf{2}_{Ar}$ by ^{1}H NMR in toluene-d_8 (Figure S7). As increasing the concentration of $\mathbf{2}_{Ar}$, the signals of the porphyrin-β protons and the aryl protons shifted to the upfield. The π-stacked microscopic binding constant was then determined to be 11 ± 1 M^{-1}.

3. Self-Sorting Properties

3.1. Self-sorting from dissociated binary mixture: Temperature variation of the double-strand $(\mathbf{1}_n)_2$ was independently examined by heated from 25 °C up to 70 °C and then cooled to 25 °C. Each spectral change showed no hysteresis in the course of temperature variation (Figure S8). Upon elevating the temperature, the split Soret band of $(\mathbf{1}_2)_2$ was converged to single degenerated band, suggesting thermal unzipping of the double-strand $(\mathbf{1}_2)_2$ despite of it being incomplete (Figure S8A). On the other hand, the split Soret band of $(\mathbf{1}_3)_2$ was only broadened at 70°C (Figure S8B), presumably attributable to conformational disordering of the double-stranded structure. Although the double-strand were too stable to be thermally unzipped, the structure of the double-strand $(\mathbf{1}_n)_2$ was somewhat thermally disturbed.

With these results in mind, temperature variation of

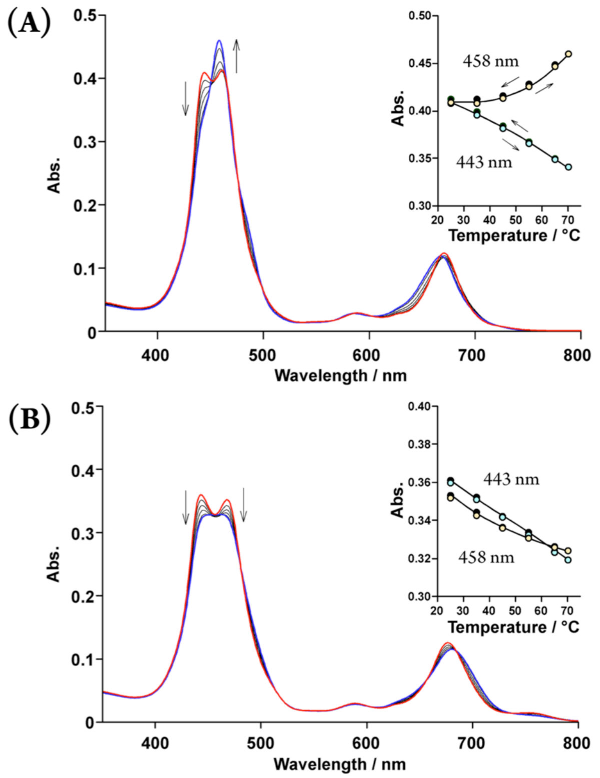

Figure S8. Absorption spectra of $(\mathbf{1}_2)_2$ ($[\mathbf{1}_2]$ = 2.3 × 10^{-6} M) (A) and $(\mathbf{1}_3)_2$ ($[\mathbf{1}_3]$ = 1.9 × 10^{-6} M) (B) in the course of temperature variation from 25 to 70 °C (red-to-blue) in toluene. The inset shows plots of the absorbance at various temperatures (upward variation; colored circle, downward variation; black circle).

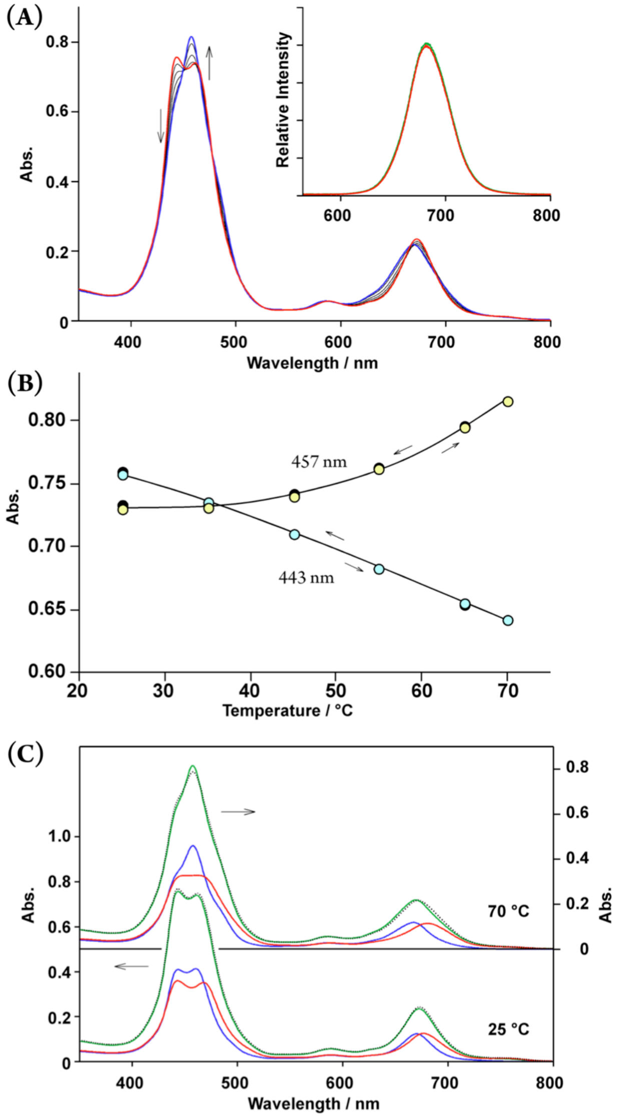

Figure S9. (A) Electronic absorption spectra of binary mixture of $(\mathbf{1}_2)_2$ and $(\mathbf{1}_3)_2$ in the course of temperature variation from 25 to 70 °C (red–blue) in toluene. The inset shows fluorescence spectra of the binary mixture at 25 °C before and after heating (l_{ex} = 450 nm). (B) Plots of the absorbance at various temperatures (upward variation; colored circle, downward variation; black circle). (C) Electronic absorption spectra of $(\mathbf{1}_2)_2$ (blue line), $(\mathbf{1}_3)_2$ (red line), their binary mixture (green line), and the summation of the spectra of $(\mathbf{1}_2)_2$ and $(\mathbf{1}_3)_2$ (dotted line) at 25 °C (lower panel) and 70 °C (upper panel).

the binary mixture of $(\mathbf{1_2})_2$ and $(\mathbf{1_3})_2$ was examined. Spectral changes of $(\mathbf{1_2})_2$ and $(\mathbf{1_3})_2$ showed no hysteresis (Figure S9), suggesting that double-strands were regenerated from the thermally disturbed mixture. At 70 °C, the spectral shape of the binary mixture did not agree with the summation of those of $(\mathbf{1_2})_2$ and $(\mathbf{1_3})_2$ (Figure S9C, upper panel), suggesting that $\mathbf{1_2}$ and $\mathbf{1_3}$ were partially interacted under thermal fluctuated conditions. On the other hand, the spectrum of the binary mixture overlapped with the summation of them at 25 °C (Figure S9C, lower panel), indicating no substantial interaction between $(\mathbf{1_2})_2$ and $(\mathbf{1_3})_2$. Moreover, the fluorescence properties perfectly restored the initial properties even after thermal treatment (Figure S9A). The result clearly indicated that thermally dissociated $\mathbf{1_2}$ and $\mathbf{1_3}$ were orthogonally assembled into the individual double-strands. Self-complementary coordination bonds, which were significantly stabilized by the synergetic effect, discriminates the difference of the length of the double-strands $(\mathbf{1_3})_2$ and $(\mathbf{1_2})_2$.

The results of the self-sorting experiments are compatible with the fact that the porphyrin array $\mathbf{1}_n$ formed exclusively the double-strand $(\mathbf{1}_n)_2$ even though the several possible self-assembled patterns via multiple coordination bonds, as shown in Scheme 4 in the main text.

4. 2D NMR Spectra

4.1. Double-strand $(\mathbf{1_1})_2$: The double-strand $(\mathbf{1_1})_2$ was identified by NMR in toluene-d_8 (Figure S10). The spectral properties of $\mathbf{1_1}$ showed no substantial change in the order of 10^{-3}–10^{-2} M in NMR spectra, while the spectral shape altered due to the concentration in the electronic absorption spectra (Figure S1). The ^{1}H NMR spectrum of $\mathbf{1_1}$ in toluene-d_8 showed large upfield shift of the protons of pyridyl group shielded by the porphyrin ring at 5×10^{-2} M in toluene-d_8, indicating self-association of $\mathbf{1_1}$. Two porphyrin rings of the dimer were in identical environment, suggesting that double-strand $(\mathbf{1_1})_2$ adopted mutually stacked conformation of two porphyrin rings in a symmetric manner. All the protons were determined by the TOCSY and NOE spectra (Figures S10). The protons showing NOE correlations with the methylene protons of the MOM-group were assigned to the porphyrin-β 8-positions, whose vicinal protons at the 7-positions were then assignable due to the TOCSY correlations with the 8-positions and the *meso*-aryl groups. The protons of the porphyrin-β 3-positions were also assigned based on the TOCSY correlations with the *meso*-aryl protons. Then, the protons of the porphyrin-β 2-positions were assigned, agreeing with the NOE correlation with the 6-pyridyl proton.

The signals of the shielded protons of the pyridyl group that shifted to upfield above 5.5 ppm disappeared when $\mathbf{1_1}$ dissolved in pyridine-d_5 (Figure S24A). The comparison inferred that the double-strand $(\mathbf{1_1})_2$ formed through self-complementary coordination bonds. Thus, the structure of the double-strand $(\mathbf{1_1})_2$ was firmly established.

4.2. Double-strand $(\mathbf{1_2})_2$: The dimeric porphyrin array $\mathbf{1_2}$ includes two non-identical porphyrin rings whose environments were different as identified by TOCSY and NOE spectra (Figure S11). The protons of the porphyrin-β 8′-positions displayed the NOE correlations with the protons of the MOM and 6-pyridyl groups. The protons of porphyrin-β 7′-positions neighboring to the 8′-positions were assigned based on the vicinal TOCSY correlations. On the other hand, the TOCSY correlation with the 6-pyridyl proton determined the 3- and 4-pyridyl protons. The 3-pyridyl proton showed the NOE correlations with the protons of the porphyrin-β 2′-positions. The protons of the porphyrin-β 3′-positions neighboring to 2′-positions were determined based on the vicinal TOCSY correlations. This assignment was compatible with the NOE correlations between the *meso*-aryl protons at the 5′-position and the protons of the porphyrin-β 3′- and 7′-positions. The broad singlet of the aryl proton at the 5′-position indicates the fast rotation of the *meso*-aryl groups, suggesting large distortion of one of the porphyrin rings, because it is known that reduced steric confliction of the *meso*-aryl protons with the nearest protons of the distorted porphyrin-β positions induces the fast rotation of the *meso*-aryl groups.[S7]

In the same way, the 2′-, 3′- and 6′-pyridyl protons allowed full assignment of the protons of the porphyrin-β 2-, 3-, 7- and 8-positions. Eventually, the NOE spectrum displayed two sets of the NOE correlations between the protons of two porphyrin-β positions and the pyridyl groups and the porphyrin-β positions, as marked in Figure S10B. The entire assignment was compatible with the fact that the no multiplied signals were observed.

4.3. Double-strand $(\mathbf{1_3})_2$: Discrete double-strand $(\mathbf{1_3})_2$ was identified by TOSY and NOE spectra (Figure S12). The NOE correlation with the protons of the MOM group determined the protons of the porphyrin-β 7′- and 8′-positions. The protons of the porphyrin-β 8′-positions exhibited the NOE correlations with the 6-pyridyl proton. The TOCSY correlations with the 6-pyridyl proton determined the 3- and 4-pyridyl protons. The protons of the porphyrin-β 2′-positions were assignable based on the NOE correlations with the 3-pyridyl proton. The vicinal TOCSY correlations with the porphyrin-β 2′-positions determined the protons of the 3′-positions. The broad singlet of the *meso*-aryl group at the 5′-positions showed the NOE correlations with the protons of the porphyrin-β 3′- and 7′-positions, in line with the above assignment. The broad signal of the *meso*-aryl group suggests the distortion of the terminal porphyrin ring, as described for $(\mathbf{1_2})_2$. Therefore, the MOM-substituted pyridyl group formed a pair with one of three porphyrin rings. The signals of the porphyrin rings ac-

commodated with the MOM-substituted pyridyl group were not multiplied, indicating that these protons were in identical environment. Considering full upfield shift of the pyridyl protons, the double-strand ($\mathbf{1_3}$)$_2$ adopted mutually stacked conformation of two porphyrin arrays in a symmetric manner, excluding possible self-assembled patterns other than the antiparallel arrangement (Scheme 4 in the main text).

This was supported by the following assignment. The TOCSY correlations determined two sets of signals of 2-, 3- and 6-pyridyl and 2″-, 3″- and 6″-pyridyl protons, even though the alternating assignment was possible. These pyridyl protons showed the NOE correlations with porphyrin rings. Tediously overlapped signals of

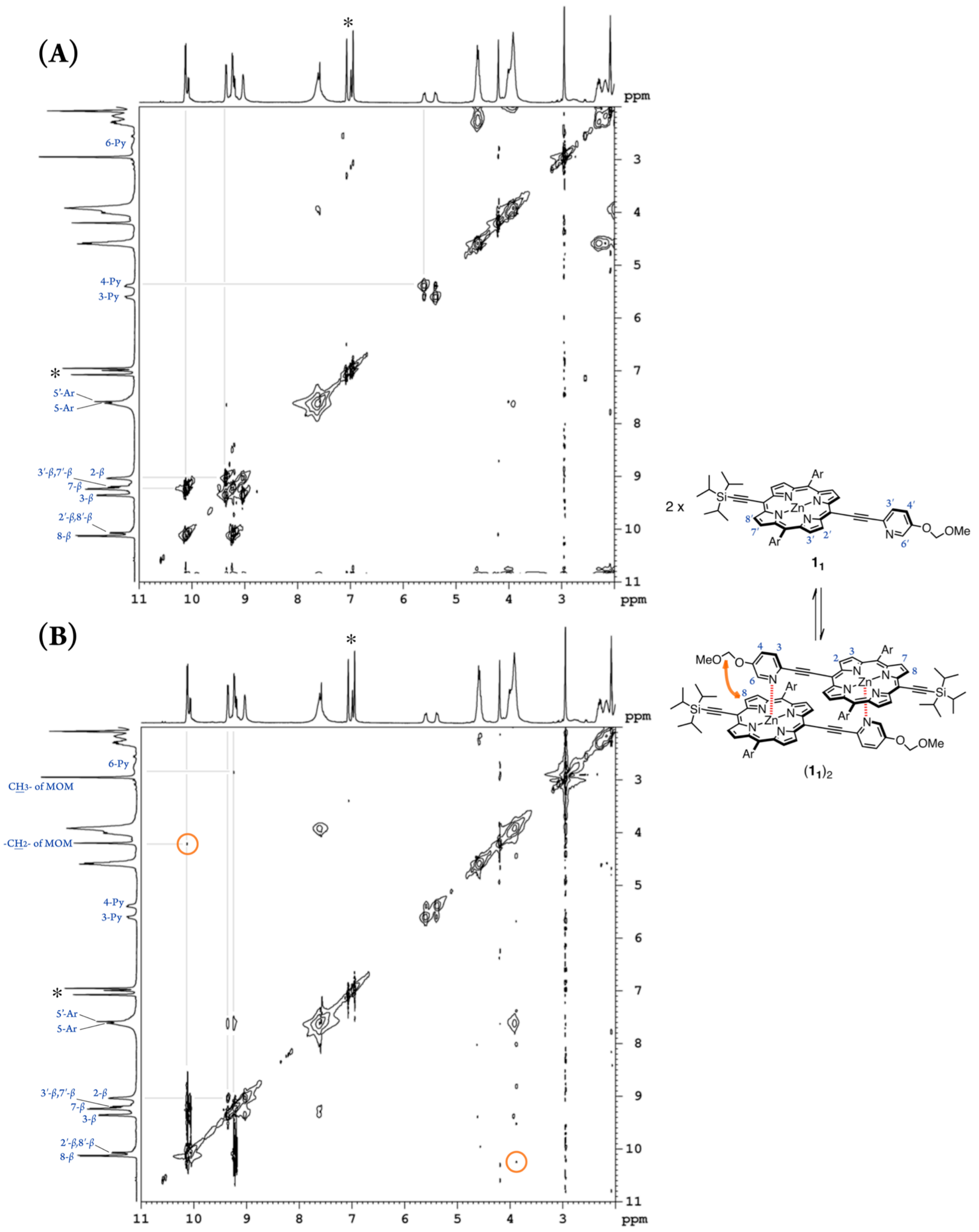

Figure S10. (A) ^{1}H–^{1}H TOCSY and (B) ^{1}H–^{1}H NOE spectra of ($\mathbf{1_1}$)$_2$ at 5 × 10^{-2} M in toluene-d_8. The asterisk indicates residual solvent.

two porphyrin-β rings disturbed the accurate assignment of these pyridyl–porphyrin pairs. In conclusion, only three sets of the porphyrin rings and pyridyl groups showed the NOE correlations. Only this assignment is satisfactory to the entire NMR observations. Alternative assignments are possible for the double-strand $(\mathbf{1_3})_2$ as shown in Figure S11, although tentative one is shown in the main text.

5. NMR Charts of Newly Synthesized Compounds

The ^{1}H and ^{13}C NMR (300 and 75 MHz, respectively) charts of newly synthesized compounds are listed below in the order described in the Synthetic Procedures. For the porphyrin derivatives, MALDI-TOF MS spectra are also shown together with the NMR charts (Figure S13–S41).

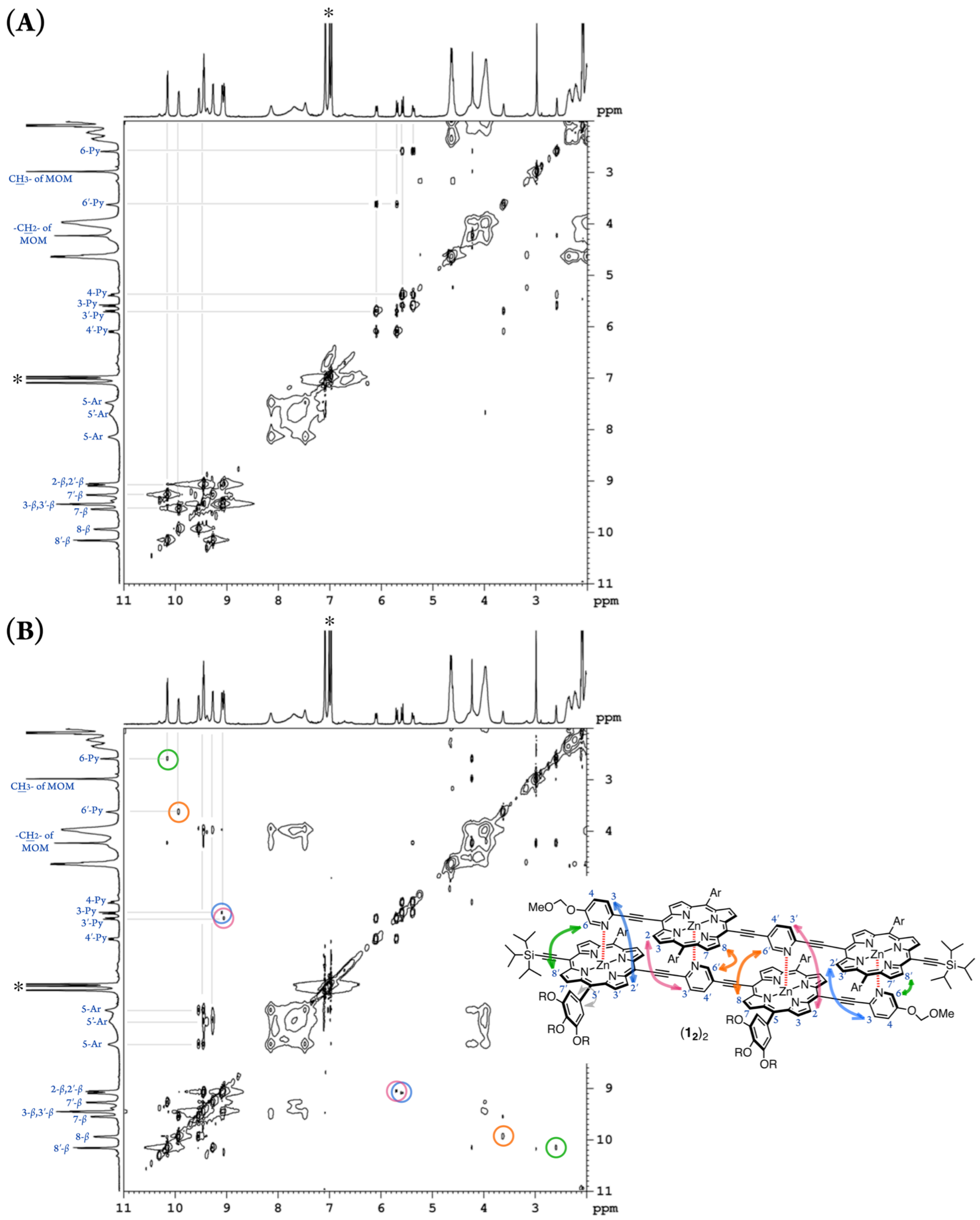

Figure S11. (A) ^{1}H–^{1}H TOCSY and (B) ^{1}H–^{1}H NOE spectra of $(\mathbf{1_2})_2$ in toluene-d_8. The asterisk indicates residual solvent.

REFERENCES

S1 D. B. Williams, M. Lawton, *J. Org. Chem.* 2010, **75**, 8351–8354.

S2 R. van Hameren, A. M. van Buul, M. A. Castriciano, V. Villari, N. Micali, P. Schön, S. Speller, L. M. Scolaro, A. E. Rowan, J. A. A. W. Elemans, R. J. M. Nolte, *Nano Lett.* 2008, **8**, 253–259.

S3 S. D. Meyer, S. L. Schreiber, *J. Org. Chem.* 1994, **59**, 7549–7552.

S4 (*a*) B. J. Littler, M. A. Miller, C.-H. Hung, R. W. Wagner, D. F. O'Shea, P. D. Boyle, J. S. Lindsey, *J. Org. Chem.* 1999, ***64***, 1391–1396.; (*b*) J. S. Manka, D. S. Lawrence, 1989, **30**, 6989–6922.

S5 M. Morisue, Y. Hoshino, M. Shimizu, M. A. Hossain, J. Hoshiba, S. Sakurai, S. Sasaki, S. S. Uemura, J. Matsui, *submitted.*

S6 B. E. Moulton, A. C. Whitwood, A. K. Duhme-Klair, J. M. Lynam, J. S. Fairlamb, *J. Org. Chem.* 2011, **76**, 5320–5334.

S7 (*a*) S. S. Eaton, G. R. Eaton, *J. Am. Chem. Soc.* 1977, **99**, 6594–6599; (*b*) Z. Zhou, X. Zhang, Q. Liu, Z. Yan, C. Lv, G. Long, *Inorg. Chem.* 2013, **52**, 10258–10263.

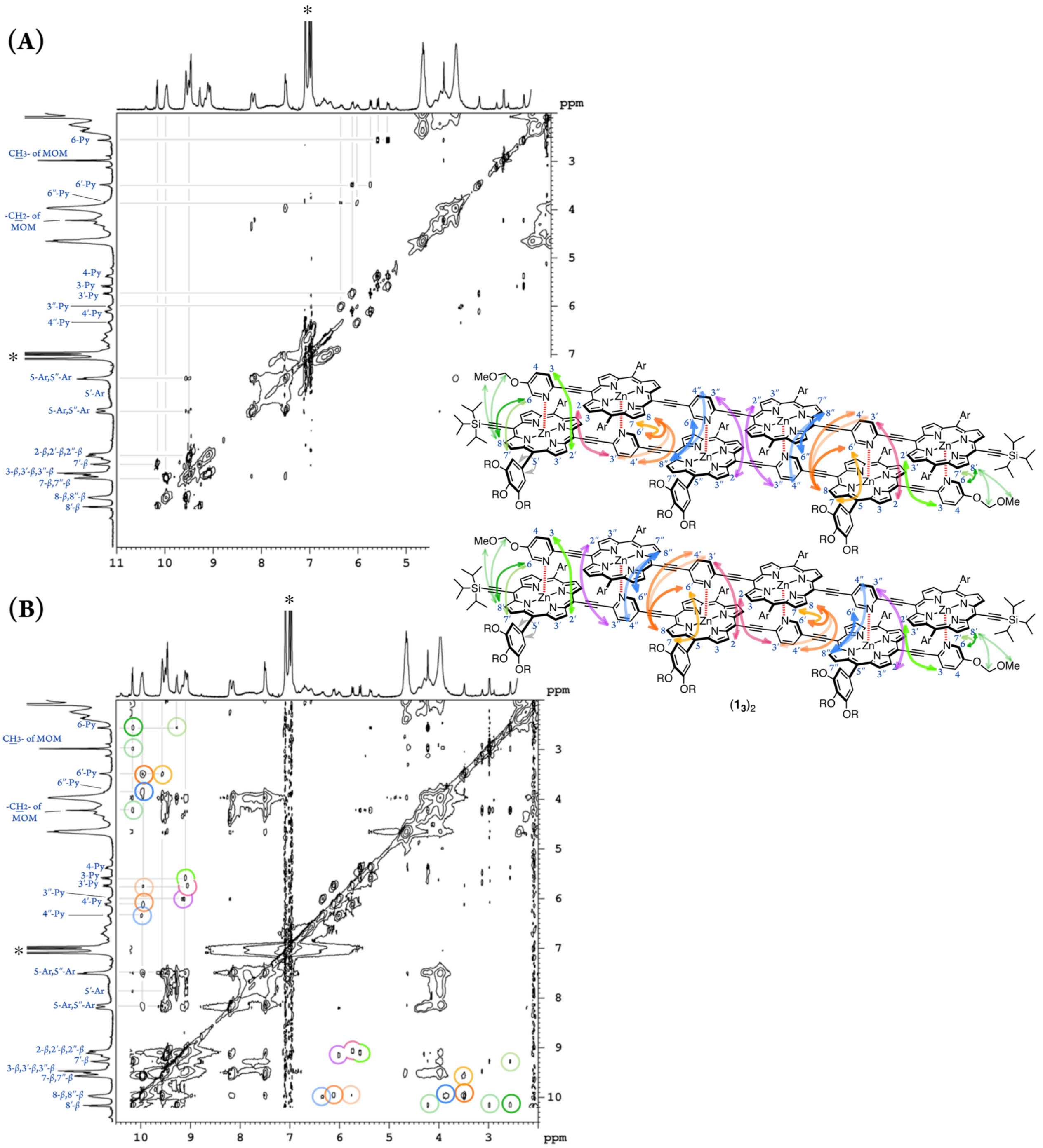

Figure S12. (A) ^{1}H–^{1}H TOCSY and (B) ^{1}H–^{1}H NOE spectra of $(\mathbf{1_3})_2$ in toluene-d_8. The asterisk indicates residual solvent.

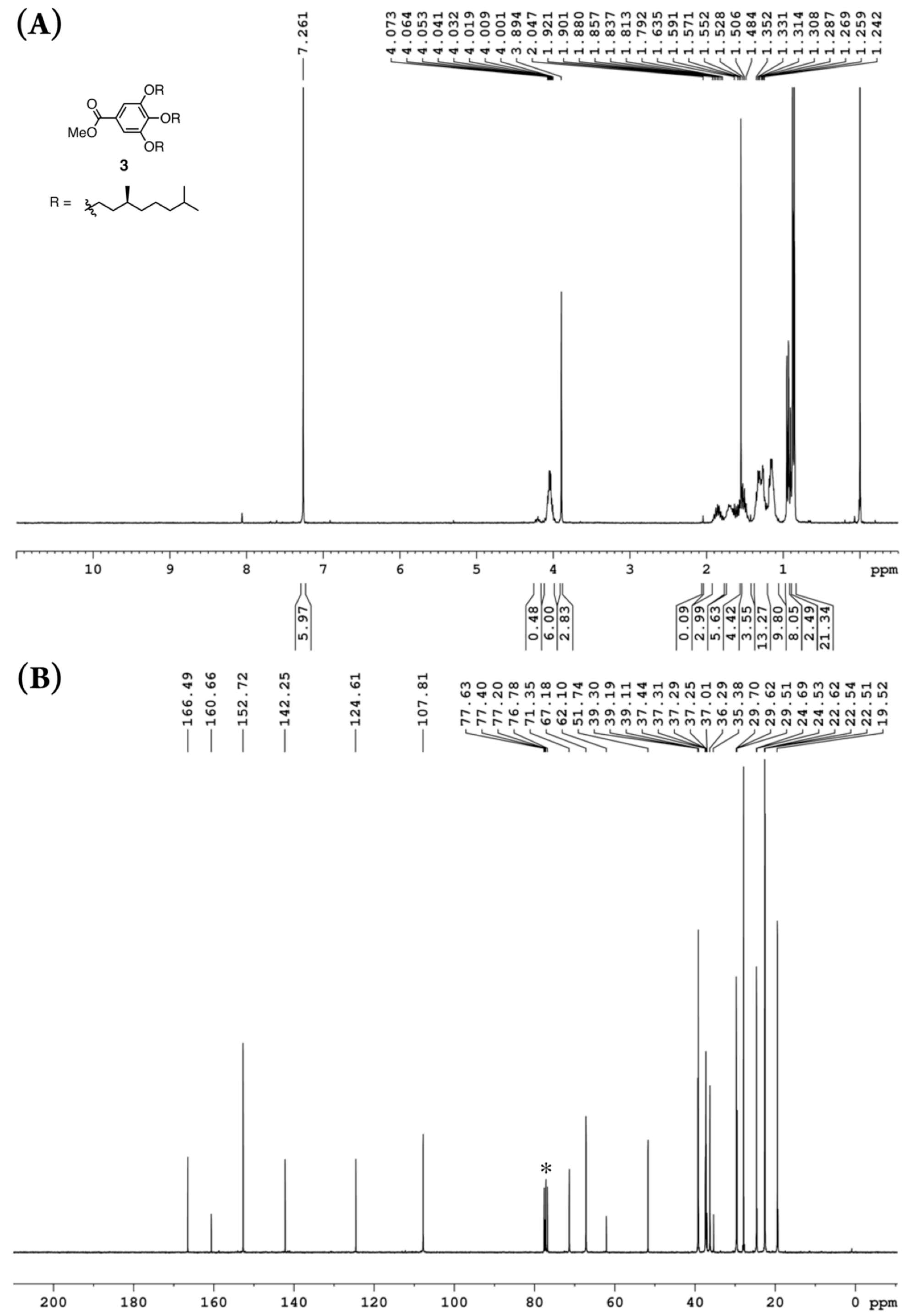

Figure S13. (A) ^{1}H and (B) ^{13}C NMR spectra of **3** in $CDCl_3$. The asterisk indicates residual solvent.

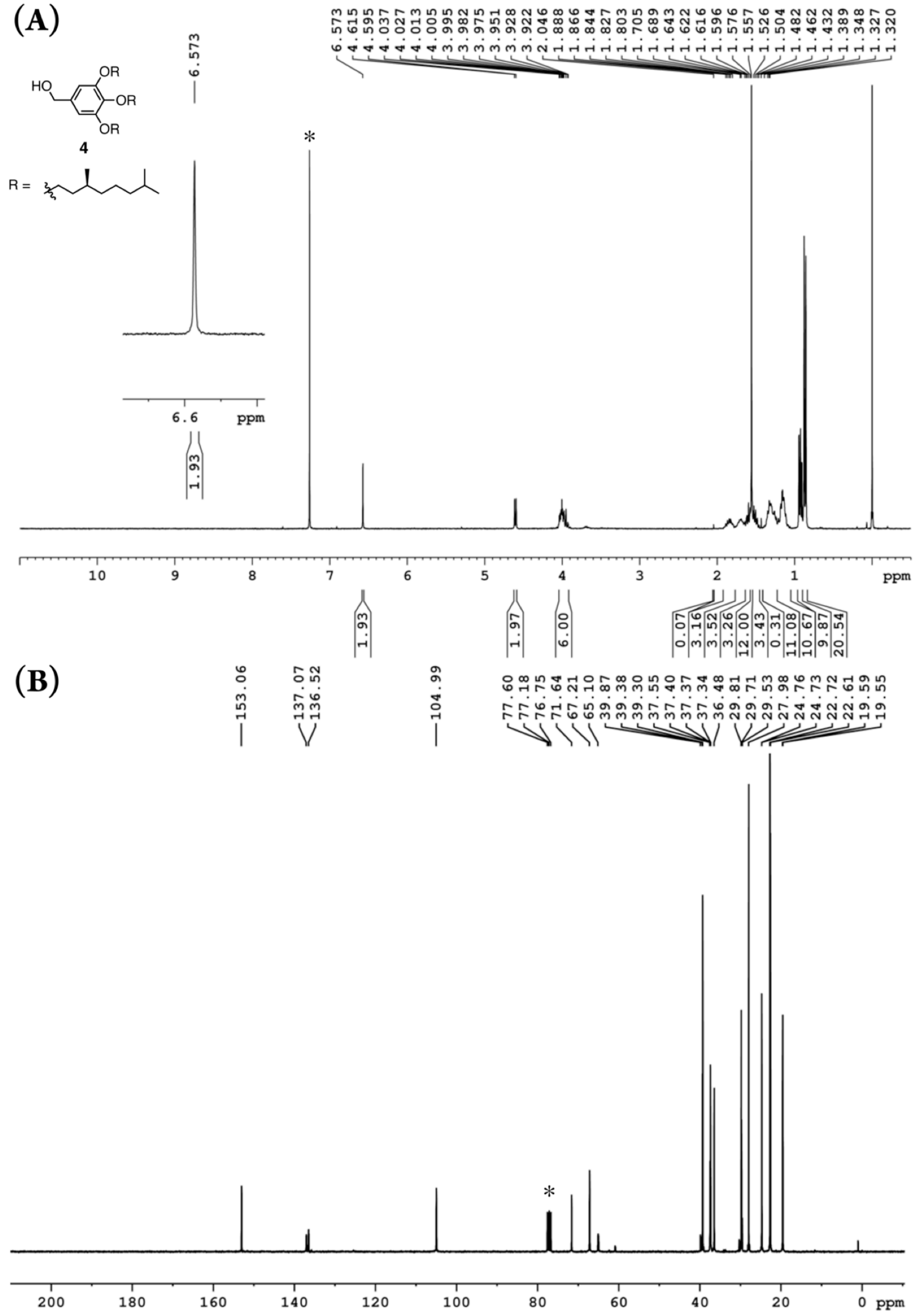

Figure S14. (A) ^{1}H and (B) ^{13}C NMR spectra of **4** in $CDCl_3$. The asterisk indicates residual solvent.

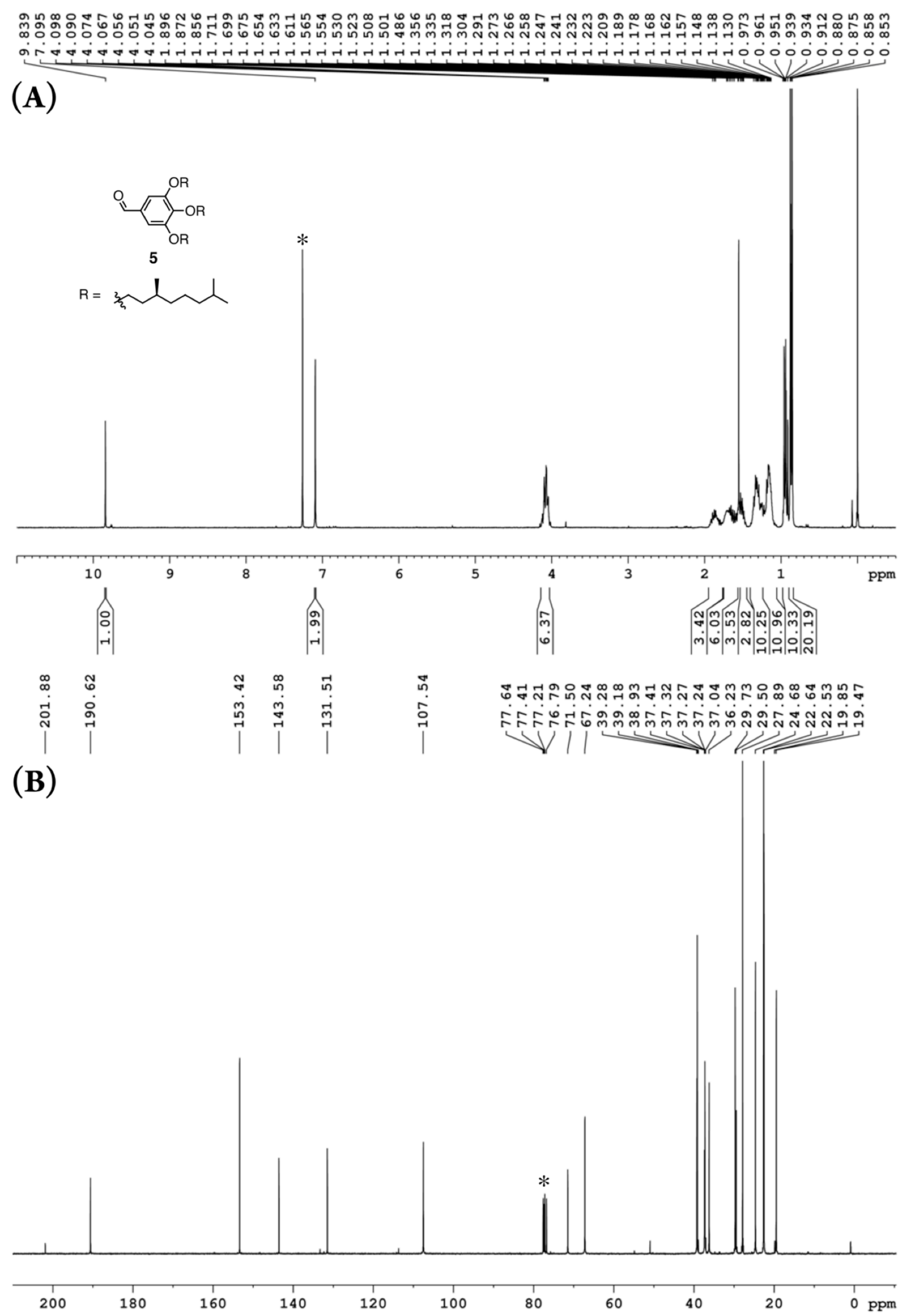

Figure S15. (A) ^{1}H and (B) ^{13}C NMR spectra of **5** in $CDCl_3$. The asterisk indicates residual solvent.

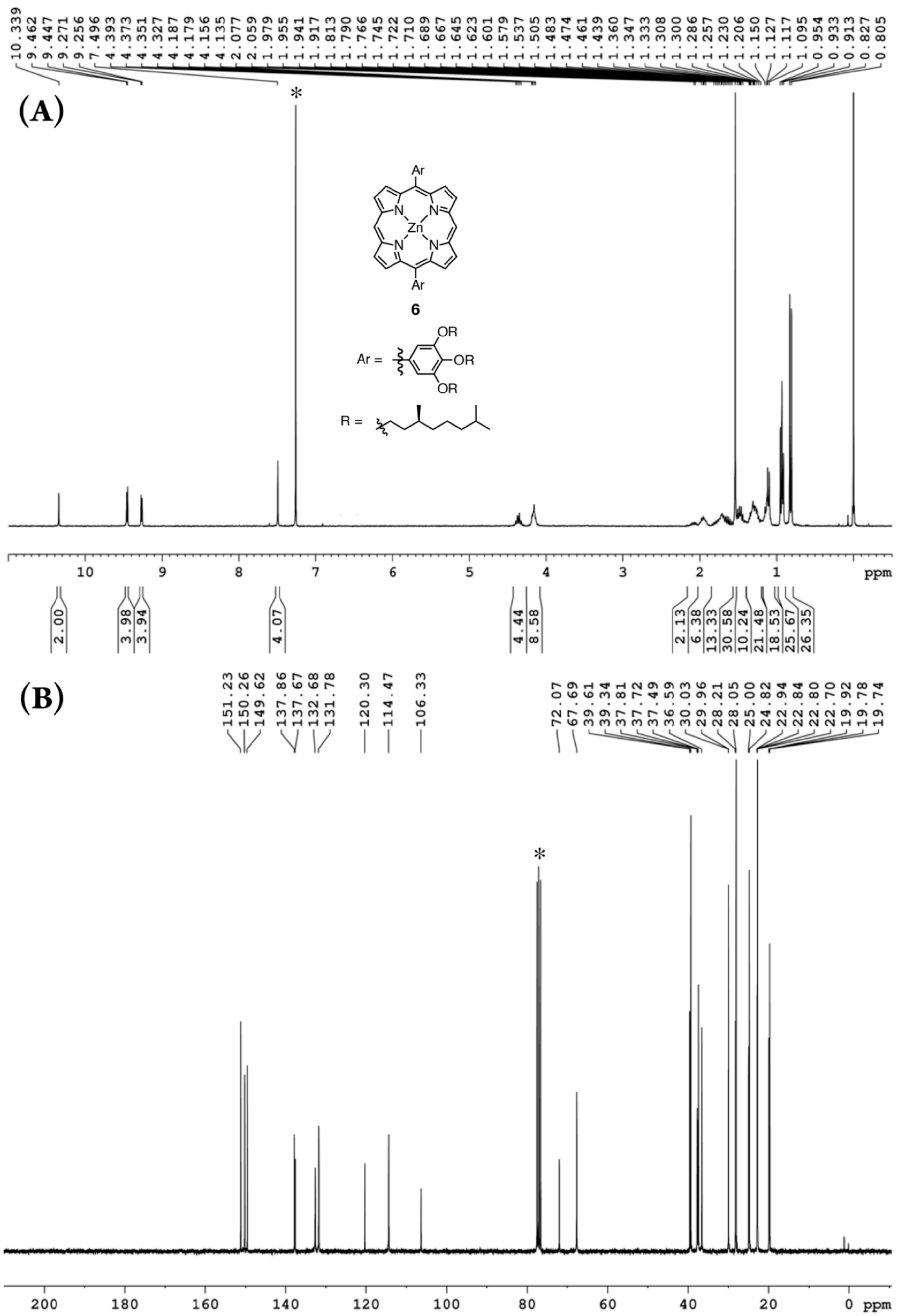

Figure S16. (A) ^{1}H and (B) ^{13}C NMR spectra of **6** in $CDCl_3$. The asterisk indicates residual solvent.

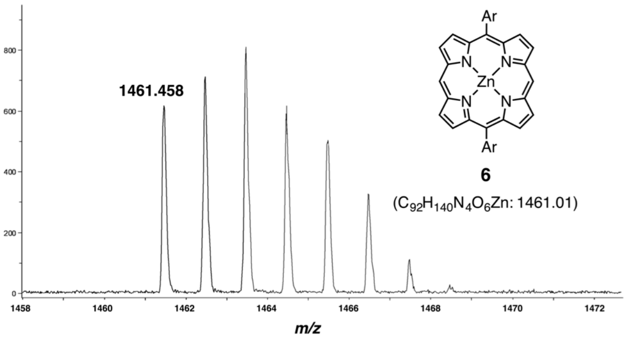

Figure S17. MALDI-TOF MS spectrum of **6**.

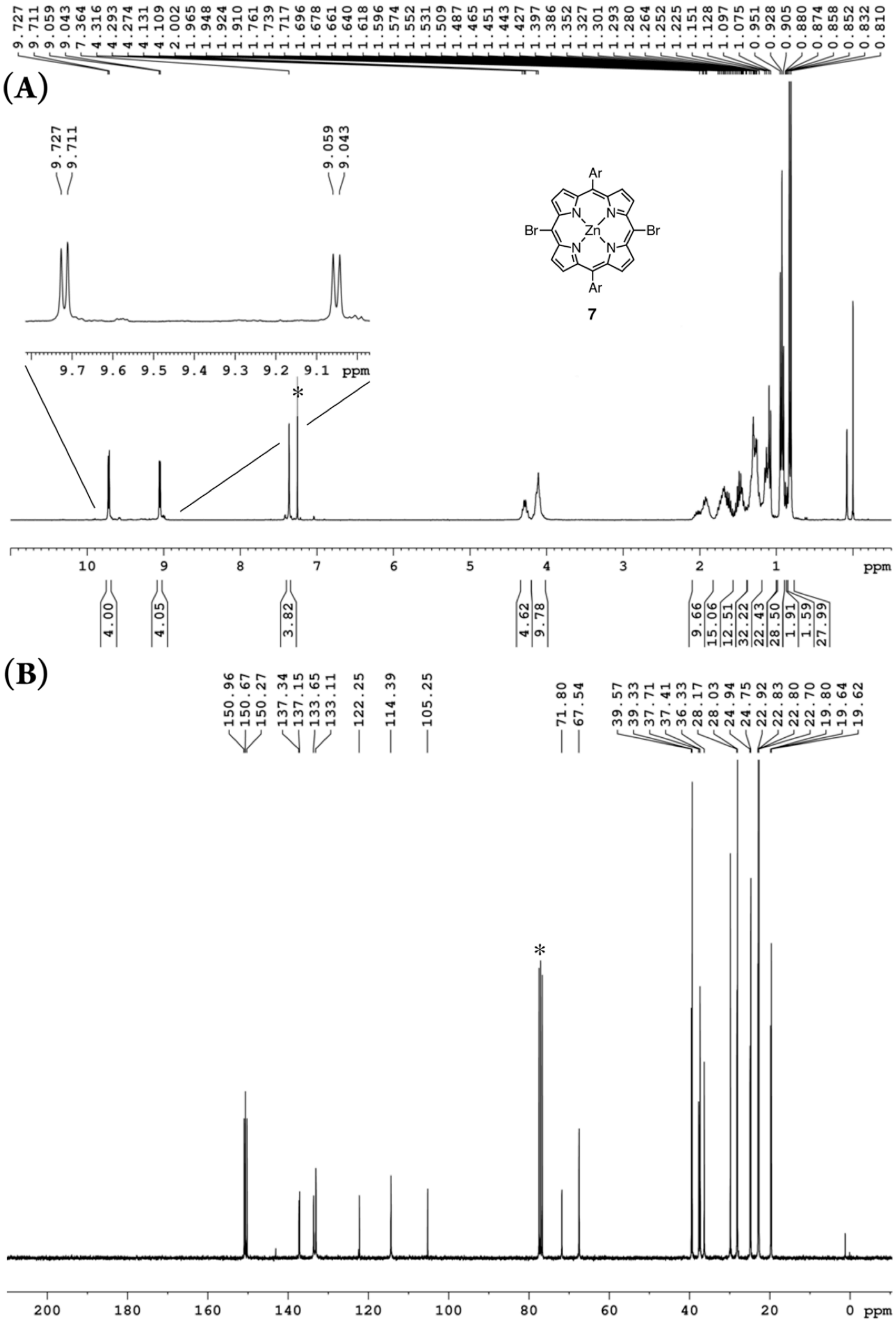

Figure S18. (A) ^{1}H and (B) ^{13}C NMR spectra of **7** in $CDCl_3$. The asterisk indicates residual solvent.

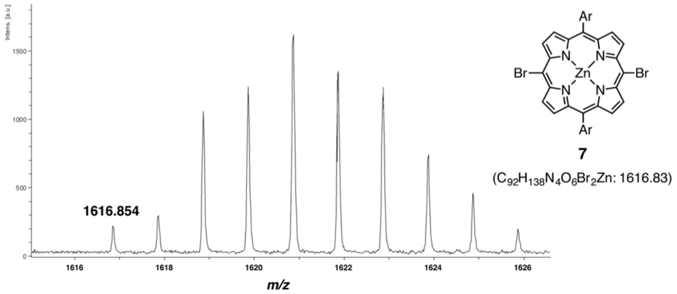

Figure S19. MALDI-TOF MS spectrum of **7**.

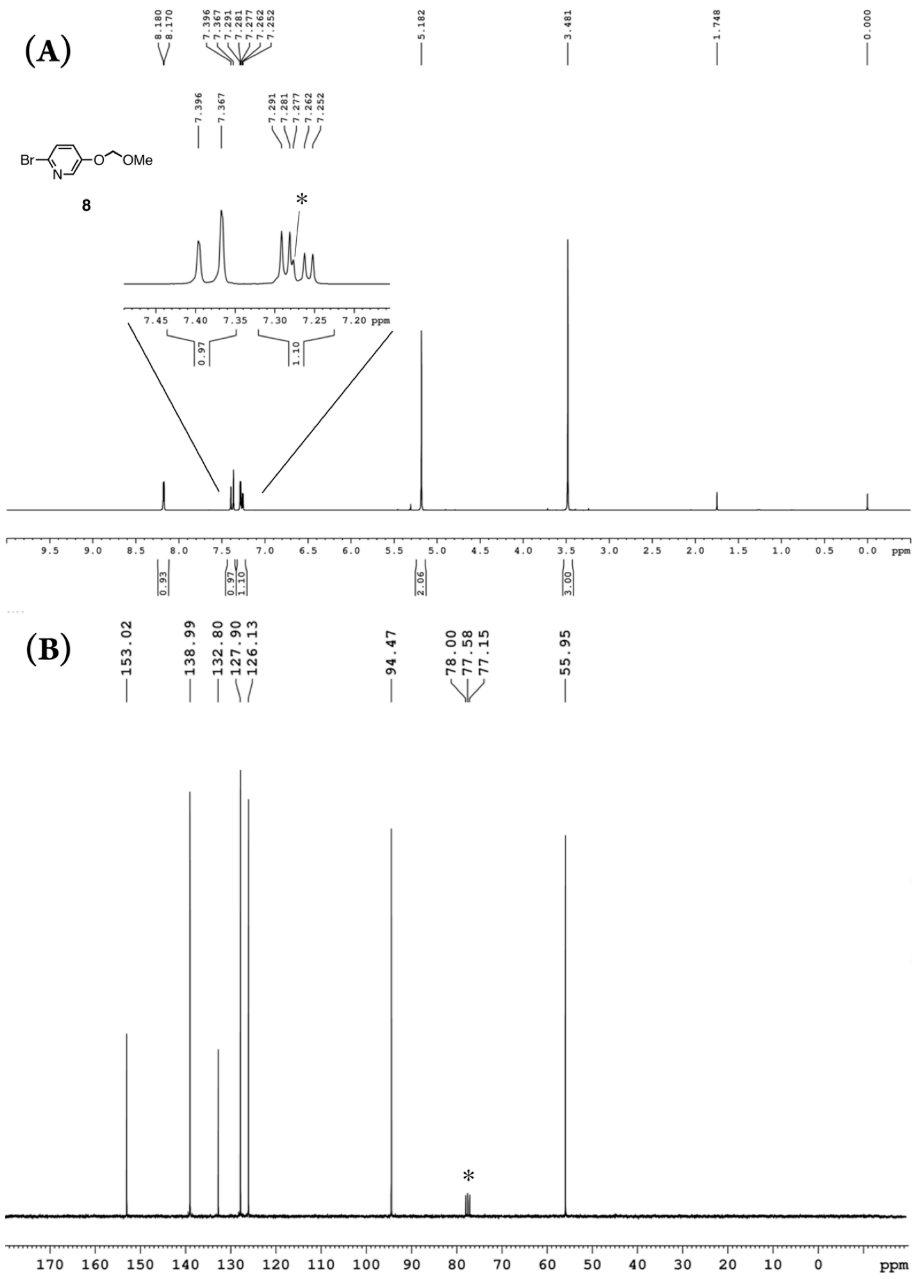

Figure S20. (A) ^{1}H and (B) ^{13}C NMR spectra of **8** in $CDCl_3$. The asterisk indicates residual solvent.

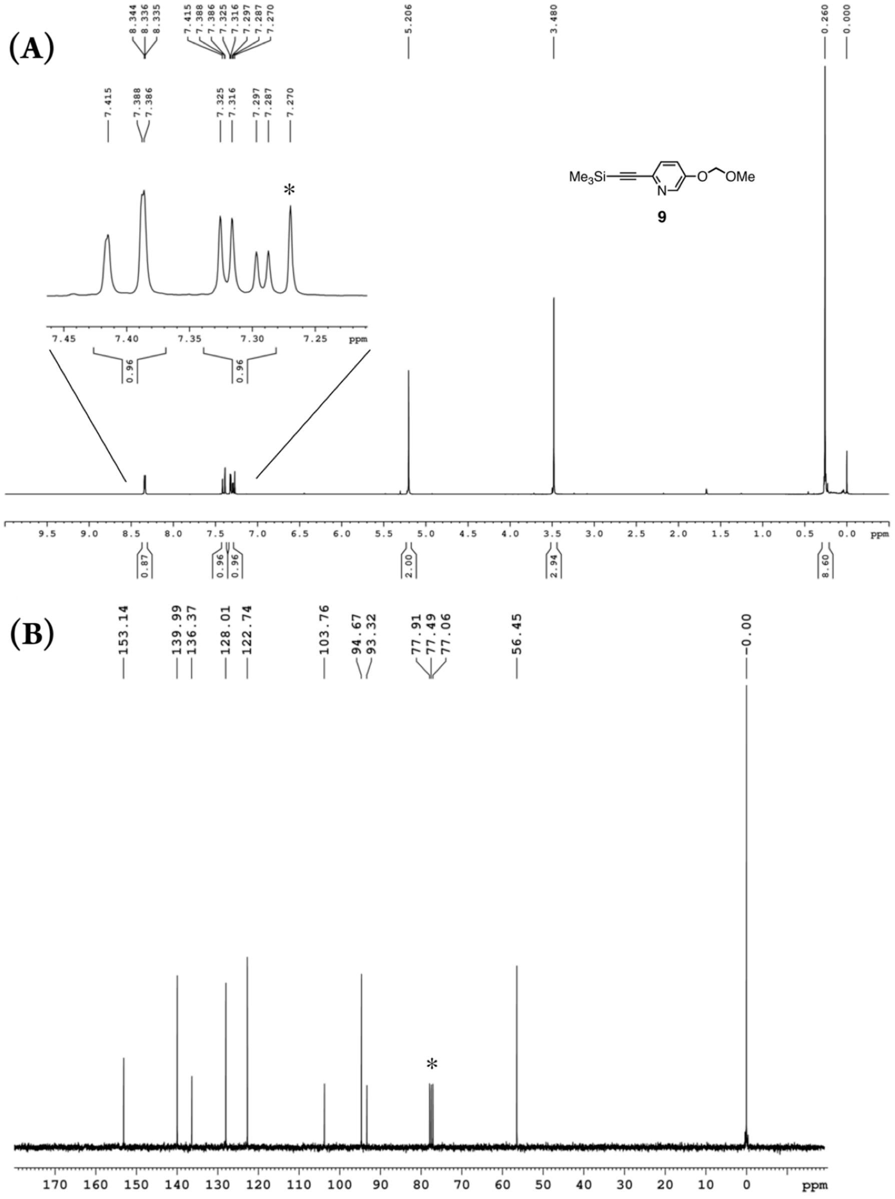

Figure S21. (A) ^{1}H and (B) ^{13}C NMR spectra of **9** in $CDCl_3$. The asterisk indicates residual solvent.

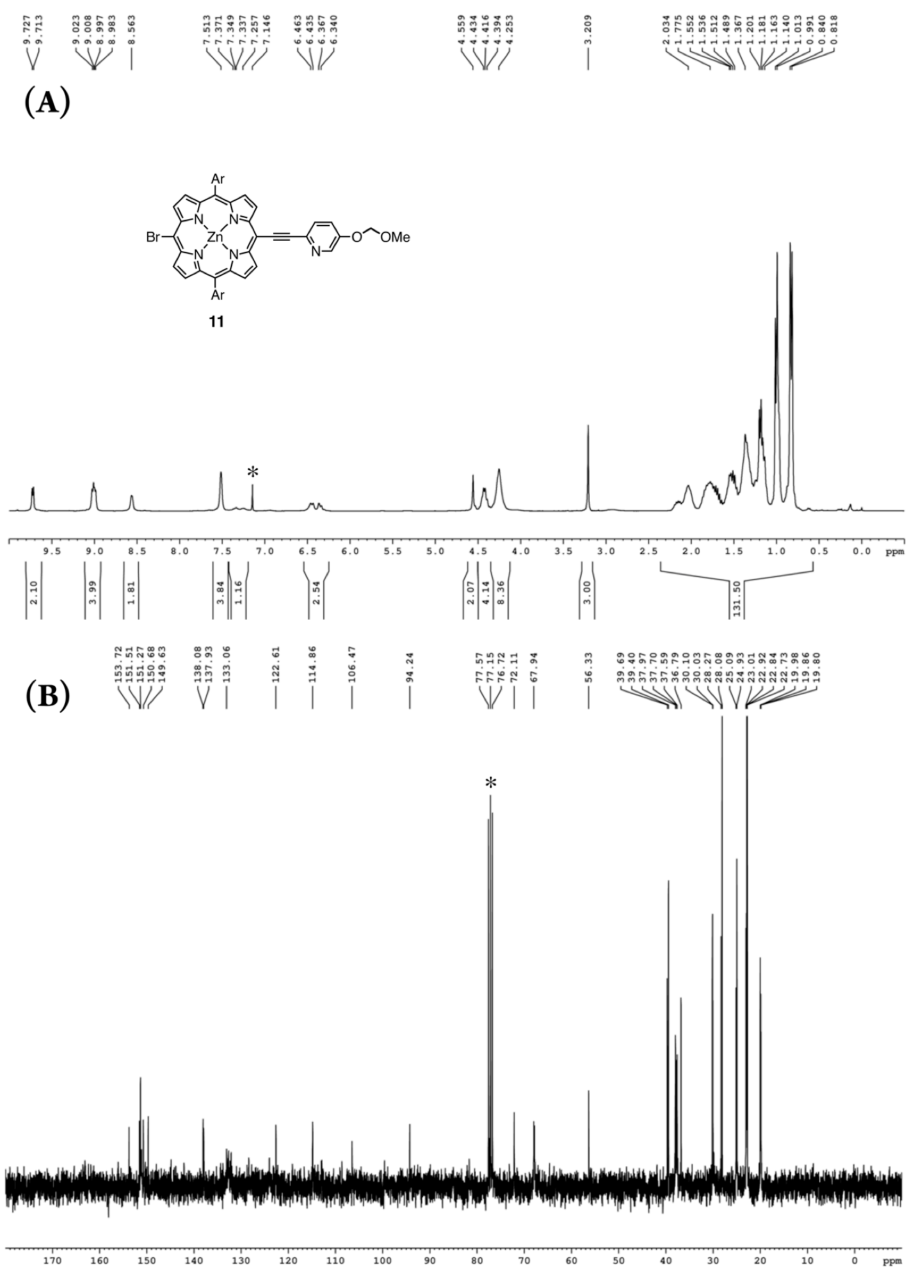

Figure S22. (A) ^{1}H and (B) ^{13}C NMR spectra of **11** in $CDCl_3$. The asterisk indicates residual solvent.

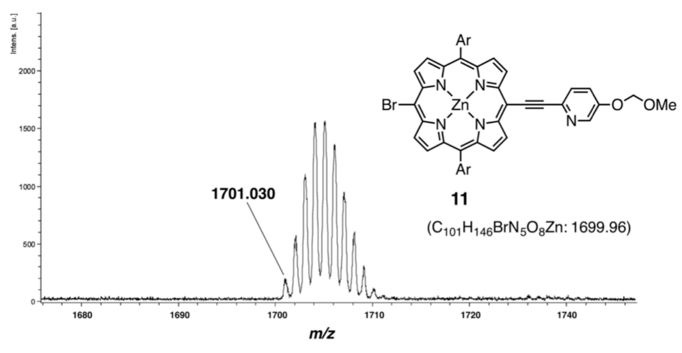

Figure S23. MALDI-TOF MS spectrum of **11**.

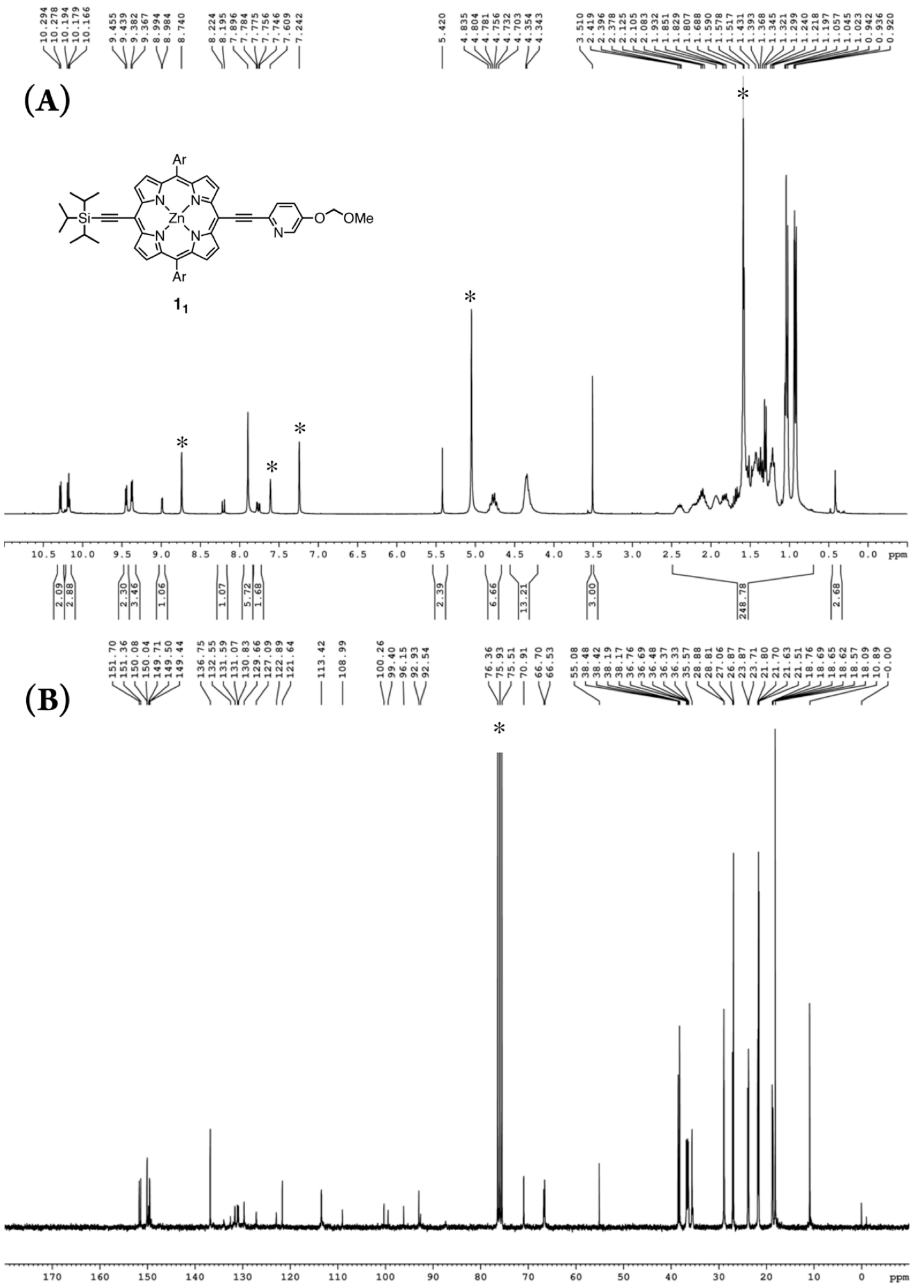

Figure S24. (A) ^{1}H NMR spectrum of $\mathbf{1_1}$ in pyridine-d_5. (B) ^{13}C NMR spectrum of $\mathbf{1_1}$ in $CDCl_3$. The asterisk indicates residual solvent.

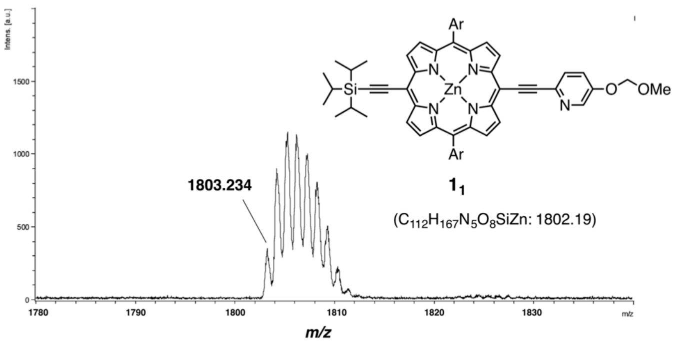

Figure S25. MALDI-TOF MS spectrum of $\mathbf{1_1}$.

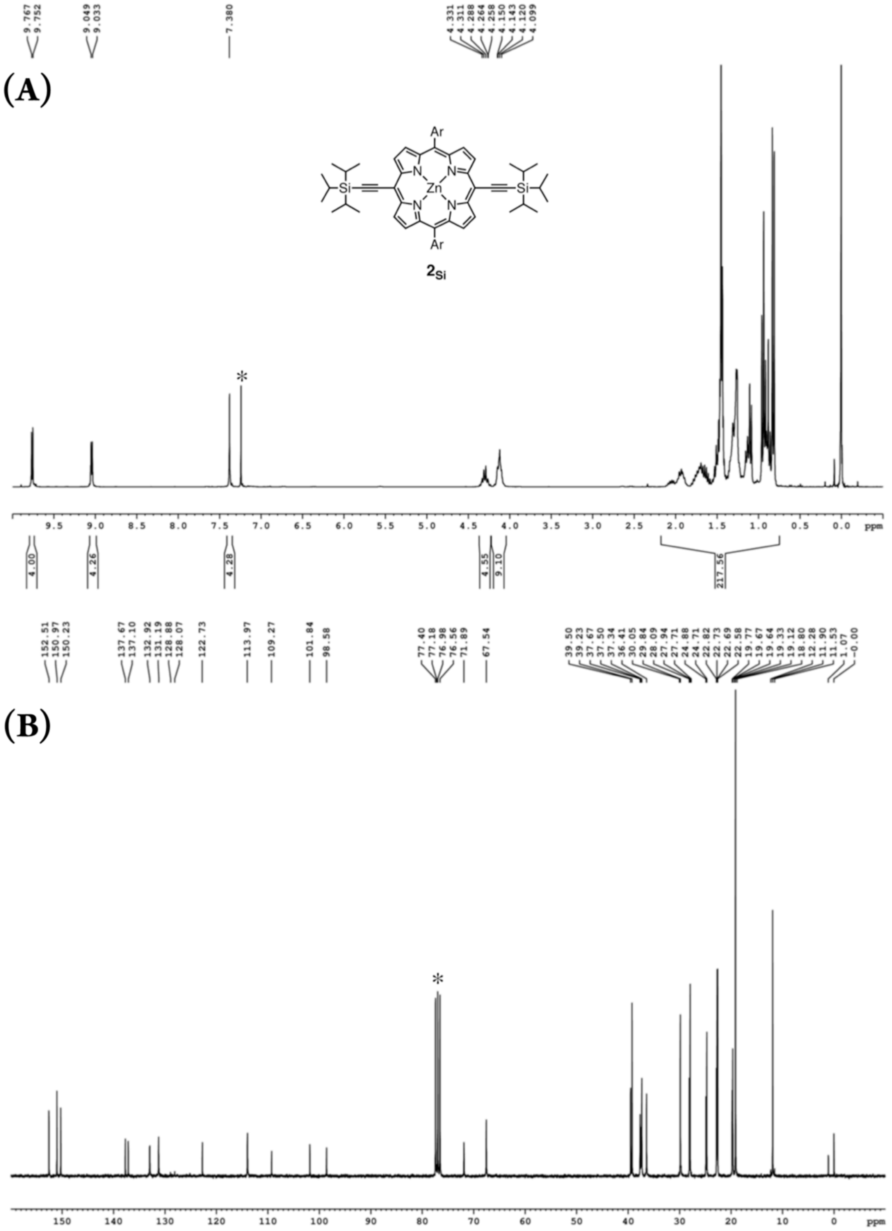

Figure S26. (A) ^{1}H and (B) ^{13}C NMR spectra of $\mathbf{2_{Si}}$ in $CDCl_3$. The asterisk indicates residual solvent.

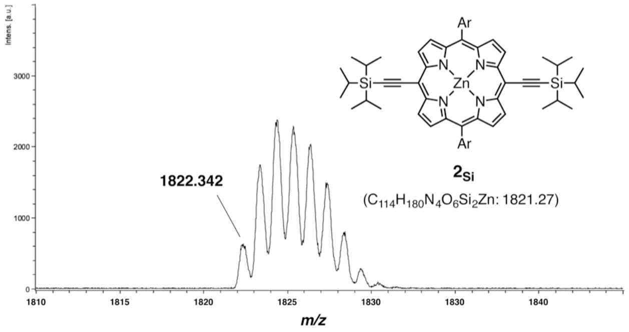

Figure S27. MALDI-TOF MS spectrum of $\mathbf{2_{Si}}$.

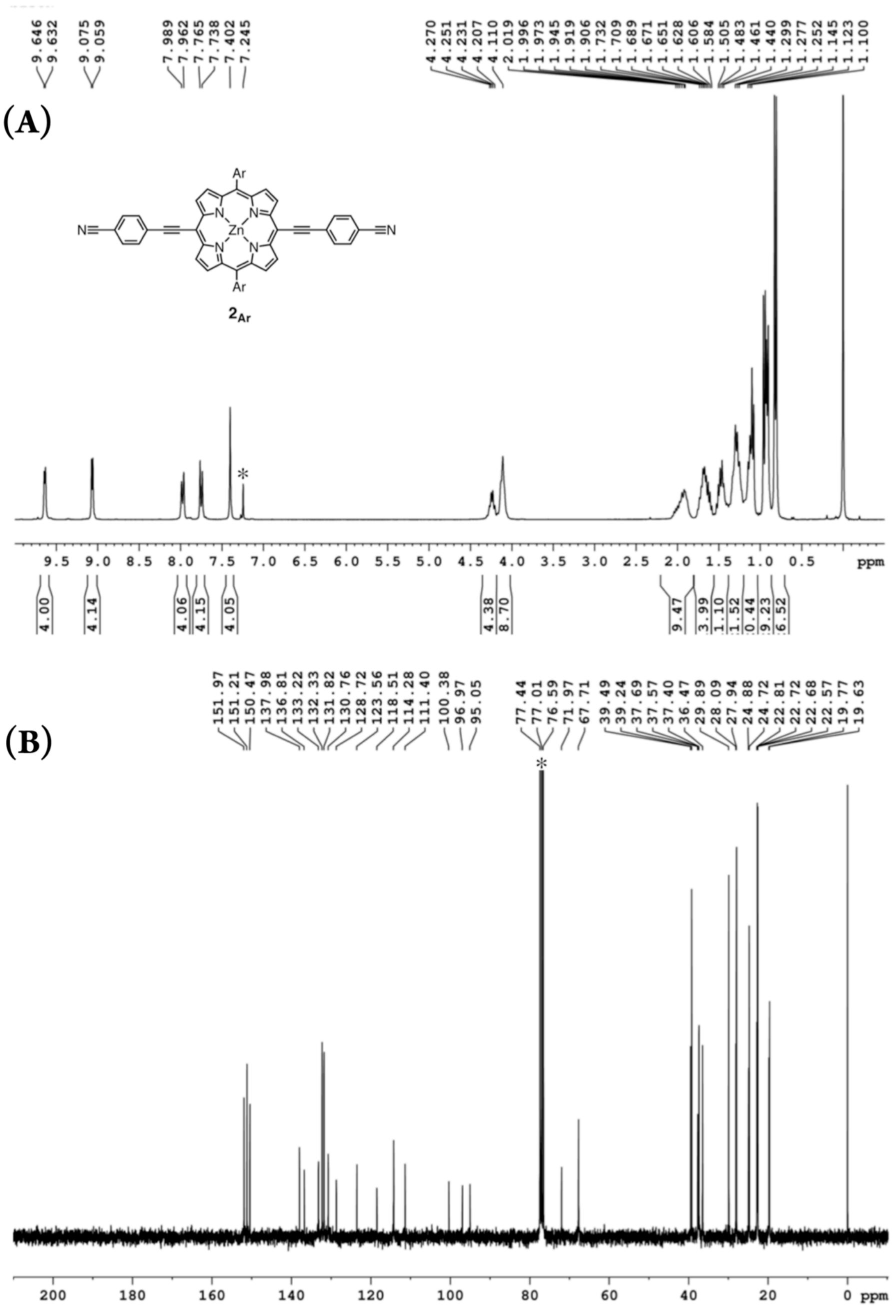

Figure S28. (A) ^{1}H and (B) ^{13}C NMR spectra of $\mathbf{2_{Ar}}$ in $CDCl_3$. The asterisk indicates residual solvent.

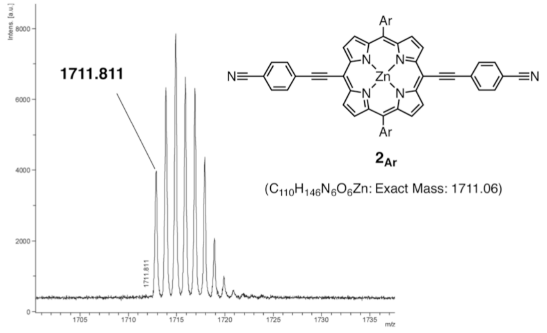

Figure S29. MALDI-TOF MS spectrum of $\mathbf{2_{Ar}}$.

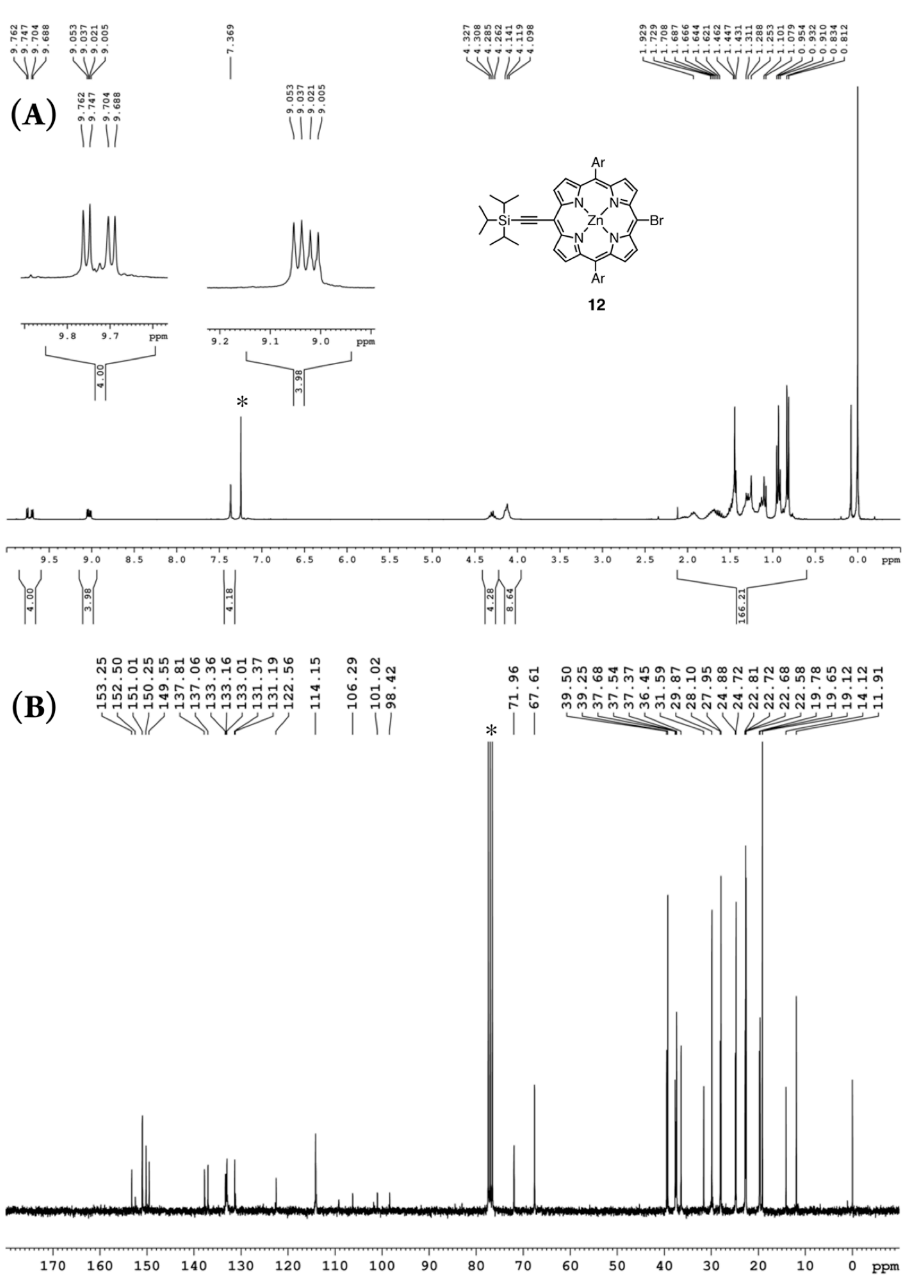

Figure S30. (A) ^{1}H and (B) ^{13}C NMR spectra of **12** in $CDCl_3$. The asterisk indicates residual solvent.

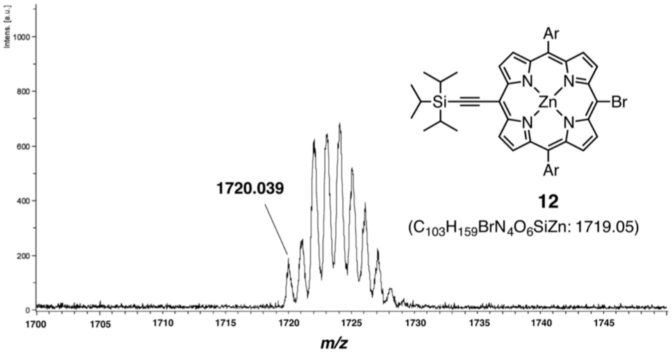

Figure S31. MALDI-TOF MS spectrum of **12**.

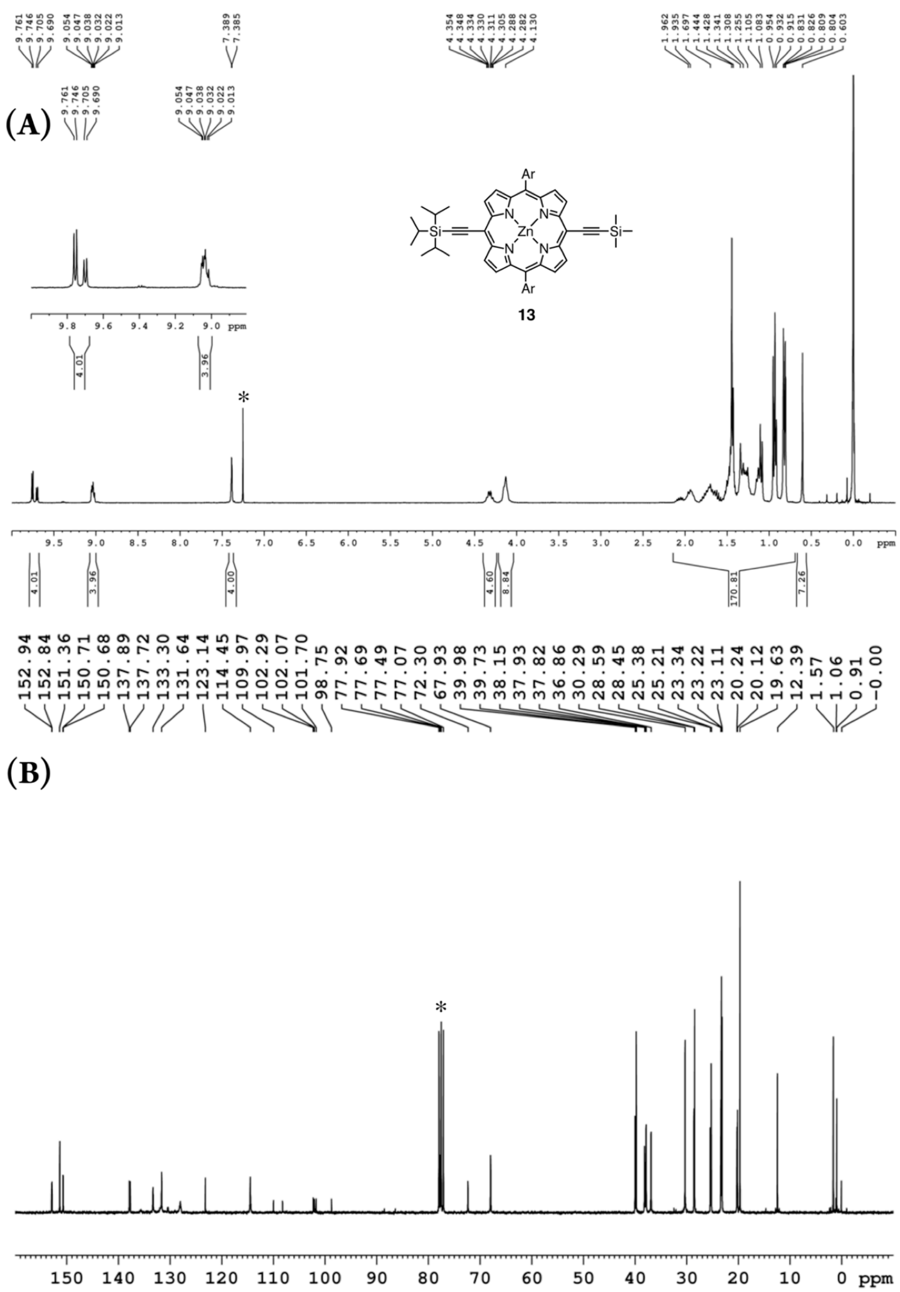

Figure S32. (A) ^{1}H and (B) ^{13}C NMR spectra of **13** in $CDCl_3$. The asterisk indicates residual solvent.

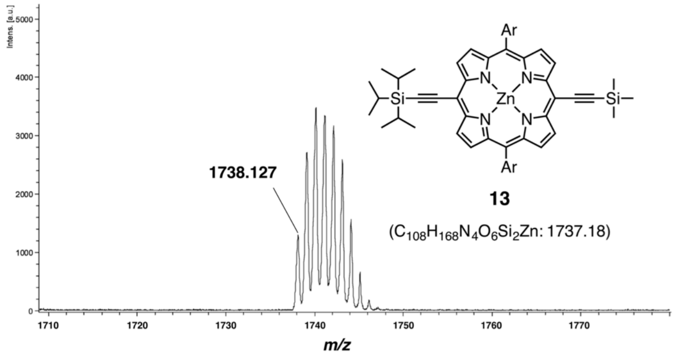

Figure S33. MALDI-TOF MS spectrum of **13**.

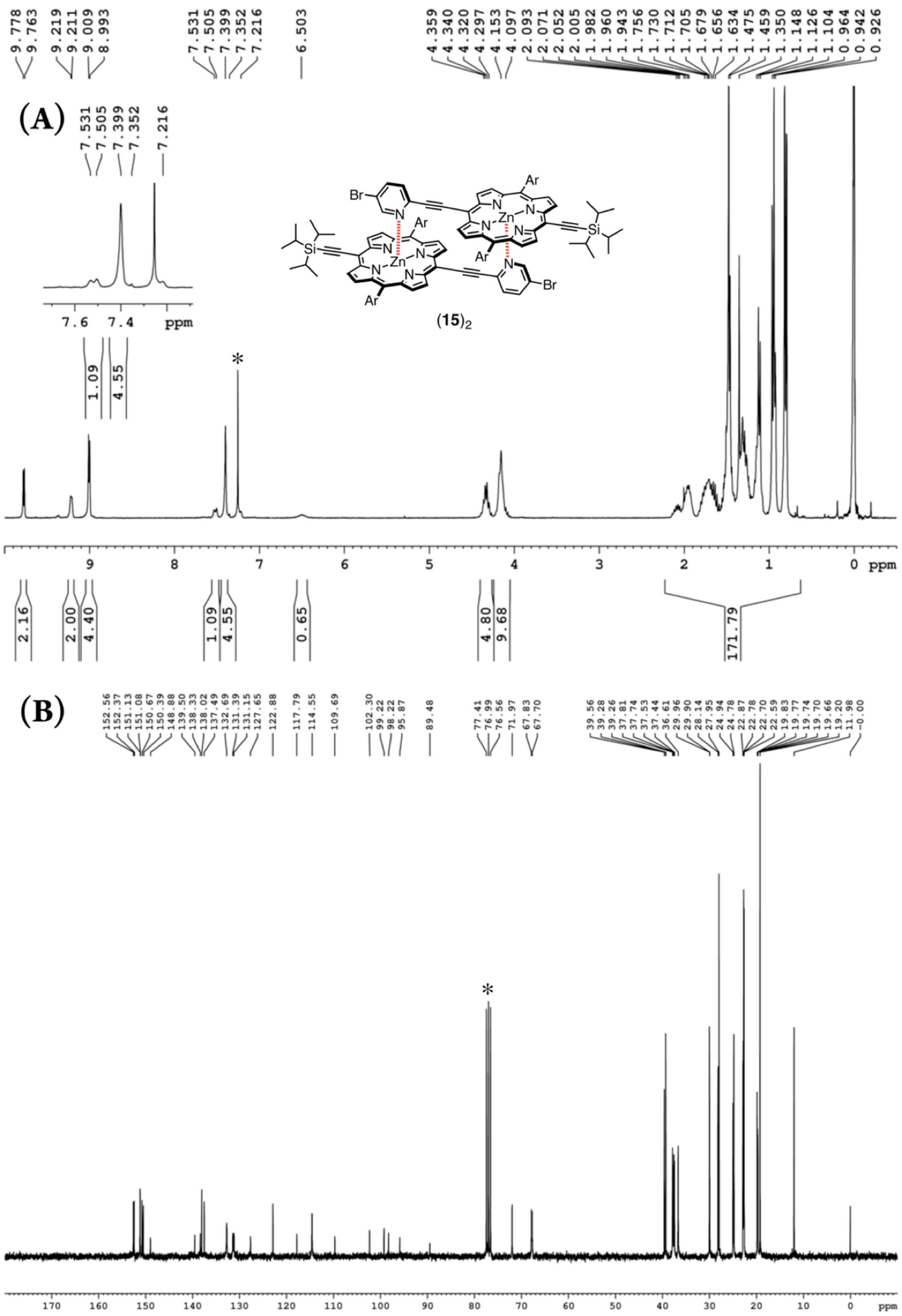

Figure S34. (A) ^{1}H and (B) ^{13}C NMR spectra of **15** in $CDCl_3$. The asterisk indicates residual solvent.

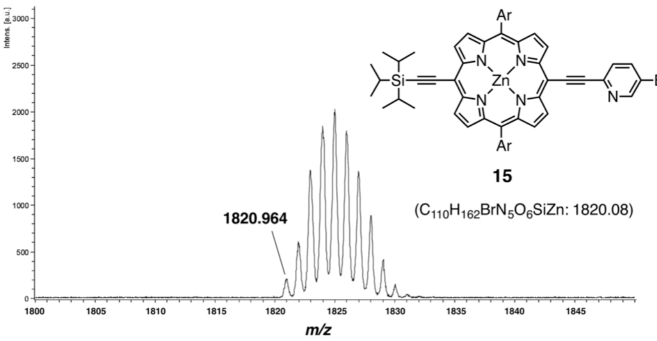

Figure S35. MALDI-TOF MS spectrum of **15**.

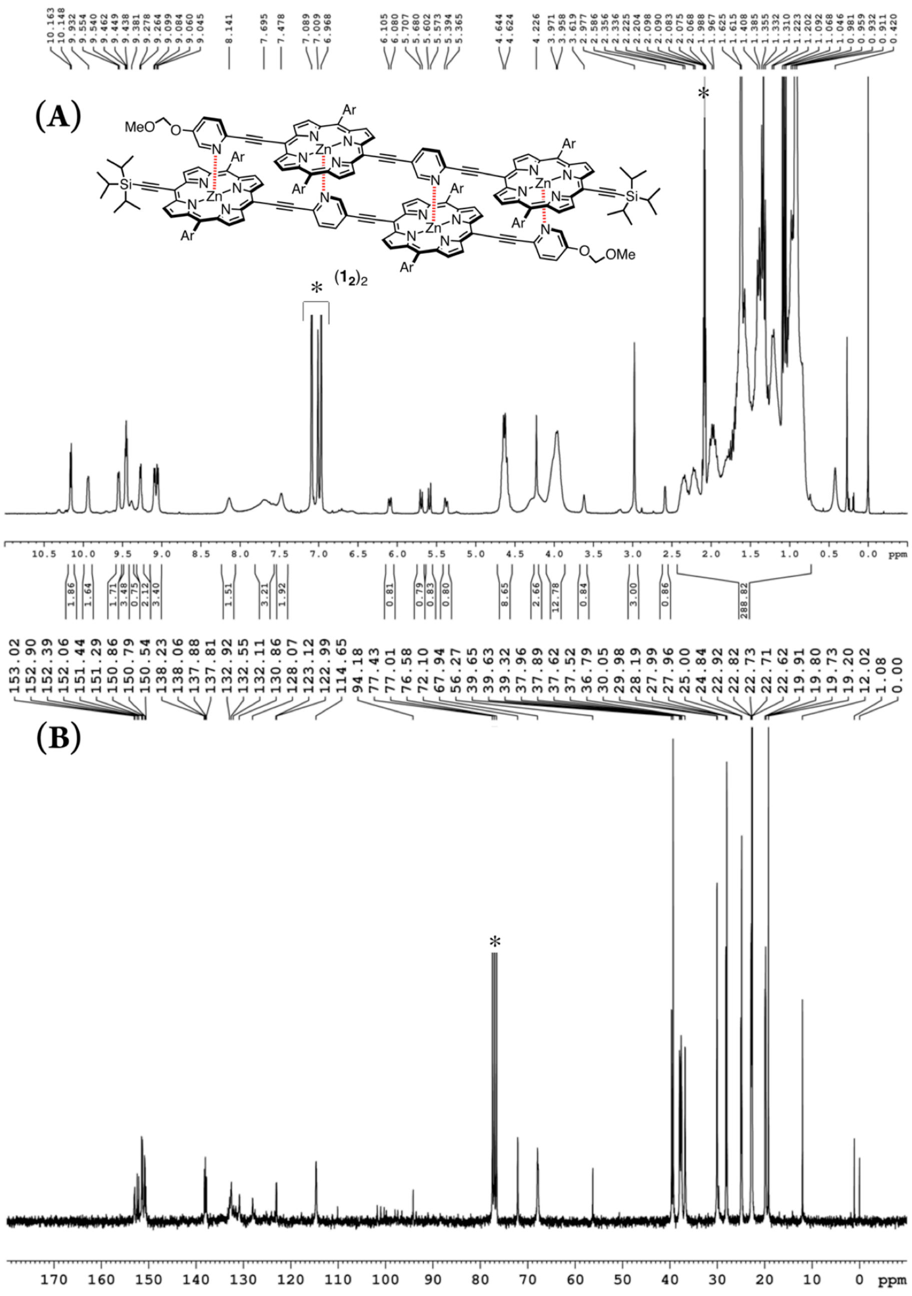

Figure S36. (A) ^{1}H NMR spectrum of $(\mathbf{1_2})_2$ in toluene-d_8. (B) ^{13}C NMR spectrum of $\mathbf{1_2}$ in $CDCl_3$. The asterisk indicates residual solvent.

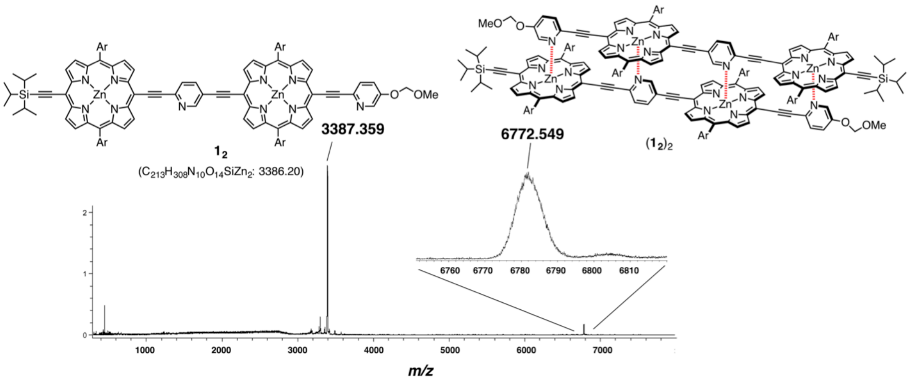

Figure S37. MALDI-TOF MS spectrum of $(\mathbf{1_2})_2$.

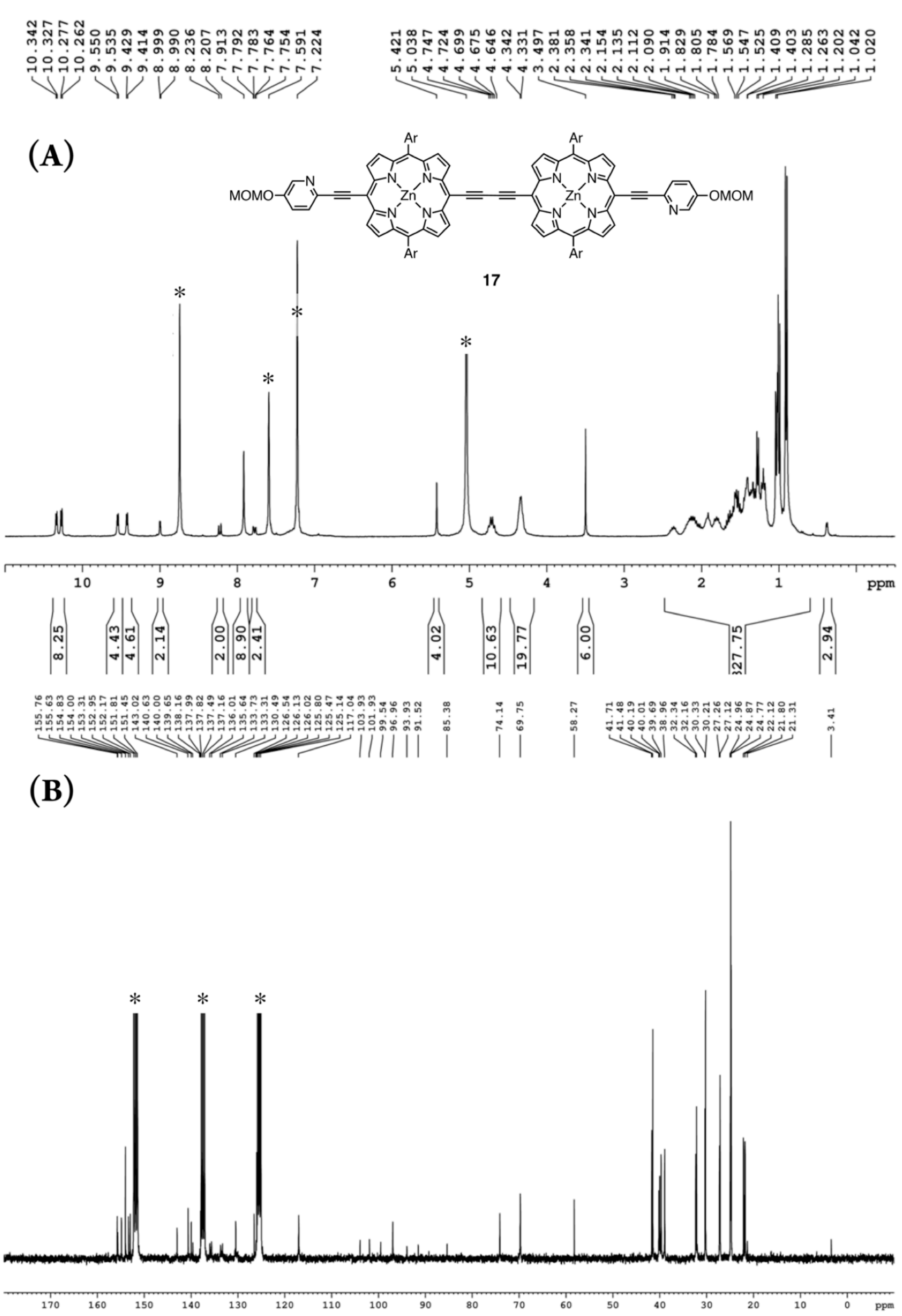

Figure S38. (A) ^{1}H and (B) ^{13}C NMR spectra of **17** in pyridine-d_5. The asterisk indicates residual solvent.

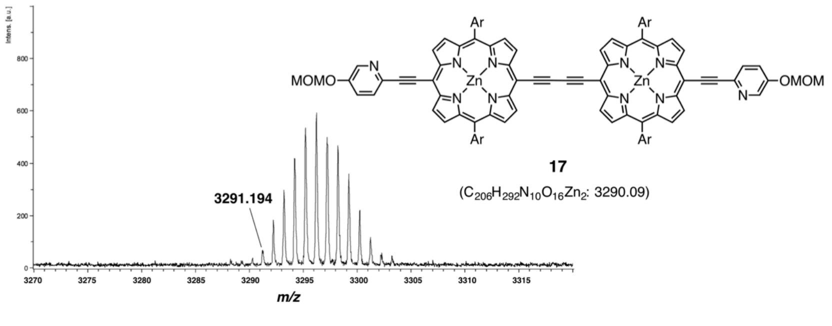

Figure S39. MALDI-TOF MS spectrum of **17**.

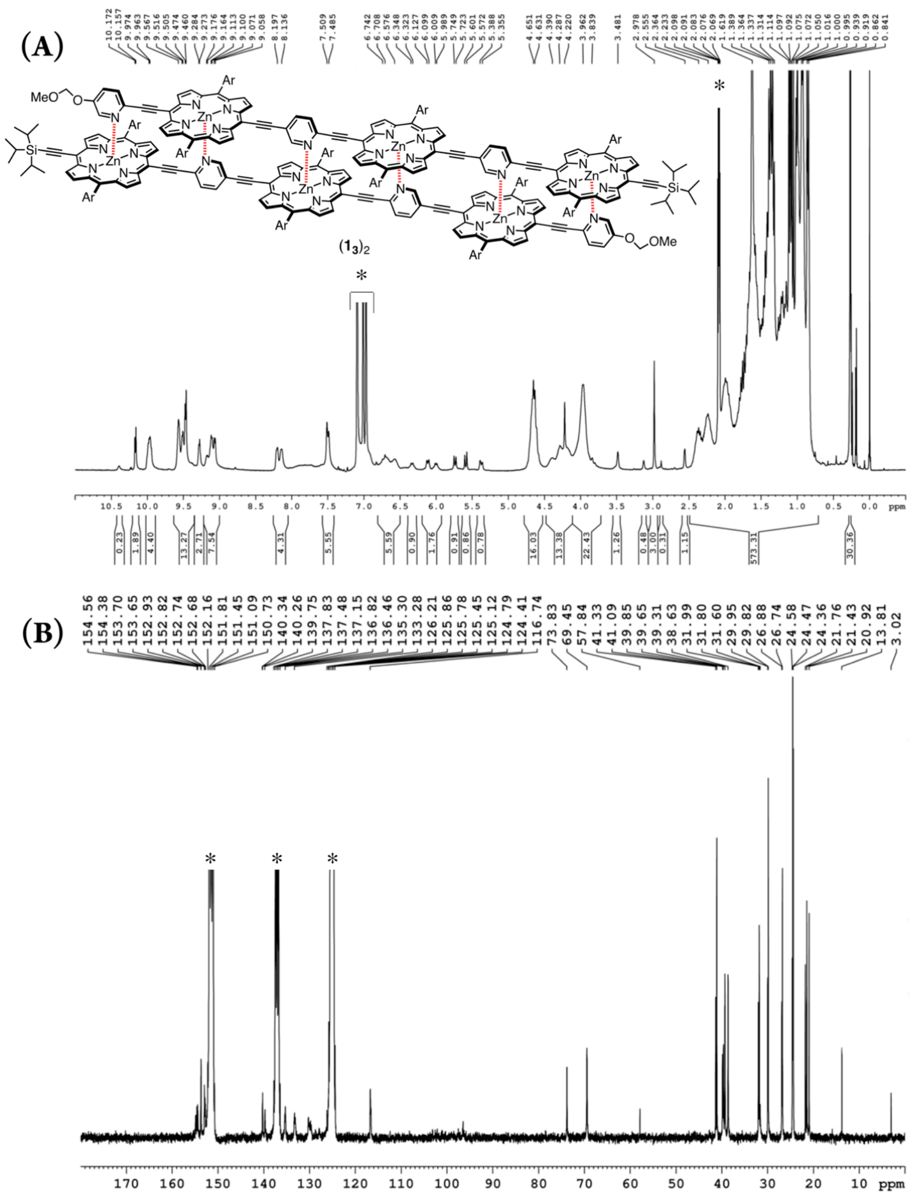

Figure S40. (A) ^{1}H NMR spectrum of $(\mathbf{1_3})_2$ in toluene-d_8. (B) ^{13}C NMR spectrum of $\mathbf{1_3}$ in pyridine-d_5. The asterisk indicates residual solvent.

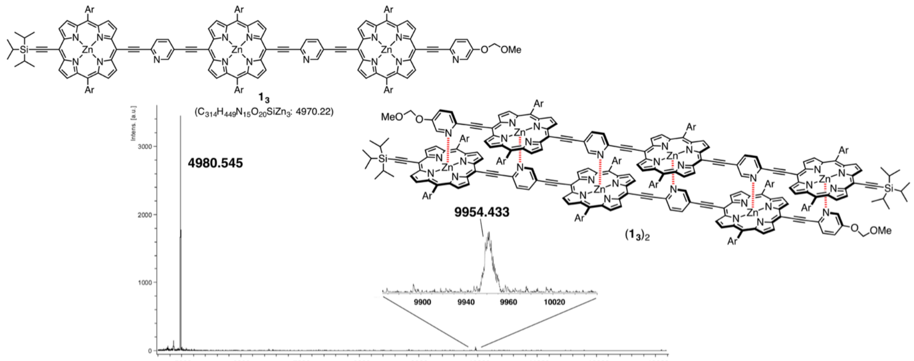

Figure S41. MALDI-TOF MS spectrum of $(\mathbf{1_3})_2$.
